# The sRNAome mining revealed existence of unique signature small RNAs derived from 5.8SrRNA from *Piper nigrum* and other plant lineages

**DOI:** 10.1038/srep41052

**Published:** 2017-02-01

**Authors:** Srinivasan Asha, E. V. Soniya

**Affiliations:** 1 Plant Molecular Biology, Rajiv Gandhi Centre for Biotechnology, Thiruvananthapuram, Kerala, 695014, India

## Abstract

Small RNAs derived from ribosomal RNAs (srRNAs) are rarely explored in the high-throughput data of plant systems. Here, we analyzed srRNAs from the deep-sequenced small RNA libraries of *Piper nigrum*, a unique magnoliid plant. The 5′ end of the putative long form of 5.8S rRNA (5.8S_L_rRNA) was identified as the site for biogenesis of highly abundant srRNAs that are unique among the *Piperaceae* family of plants. A subsequent comparative analysis of the ninety-seven sRNAomes of diverse plants successfully uncovered the abundant existence and precise cleavage of unique rRF signature small RNAs upstream of a novel 5′ consensus sequence of the 5.8S rRNA. The major cleavage process mapped identically among the different tissues of the same plant. The differential expression and cleavage of 5′5.8S srRNAs in *Phytophthora capsici* infected *P. nigrum* tissues indicated the critical biological functions of these srRNAs during stress response. The non-canonical short hairpin precursor structure, the association with Argonaute proteins, and the potential targets of 5′5.8S srRNAs reinforced their regulatory role in the RNAi pathway in plants. In addition, this novel lineage specific small RNAs may have tremendous biological potential in the taxonomic profiling of plants.

The advent of next-generation sequencing technologies has led to the discovery of functional small RNAs such as microRNAs and siRNAs from diverse organisms. In addition, the novel class of regulatory sRNAs originating from non-coding RNAs, including small nucleolar RNAs (snoRNAs)^1^ and transfer RNAs (tRNAs)^2^, were recently reported. Ribosomal RNAs (rRNA) are essential housekeeping genes, with integral roles in protein synthesis in all domains of life. The high copy number of ribosomal DNA (rDNA) genes in the genome leads to an abundance of rRNA transcripts that accounts for ~60% of the total RNA in the cell. The gene regulatory role of rRNA is under exploration. The qiRNAs (QDE-2-interacting small RNAs) generated from the rDNA locus have been reported to be induced during DNA damage in the filamentous fungi, *Neurospora crassa*^3^. The expression of specific ribosomal RNA derived fragments (rRFs) of unknown origin and function were reported from the rice embryogenic callus^4^. A novel set of rRNA derived chloroplast sRNAs were reported to be induced during heat response in the Chinese cabbage (*Brassica rapa* ssp. *chinensis*)^5^. Moreover, a deeply conserved non-canonical microRNA was recently identified from the rDNA internal transcribed spacer1 (ITS1) of the dipteran species^6^.

Multiple copies of ribosomal RNA are encoded in the genome, and the evolution of the rRNA copy number is related to cell size. In eukaryotes, the small (40S) ribosomal subunits comprise an assembly of 18SrRNA and 33 ribosomal proteins (RPs), whereas the large ribosomal units contain discrete rRNA components (5S, 5.8S and 25/28S rRNAs) and 46 ribosomal proteins. Except for 5S rRNA, all other rRNAs (18S, 5.8S, 25–28S rRNAs) are transcribed by RNA polymerase I (Pol I) as a single polycistronic unit; this unit undergoes complex post transcriptional processing into two external transcribed spacers (5′ETS and 3′ETS) and two internal transcribed spacers (ITS1 and ITS2), which flank and separate the mature rRNA components^7^. This precursor rRNA processing occurs in the nucleolus and nucleus by multiple endonucleolytic and exonucleolytic cleavage steps to produce mature rRNAs (Fig. 1). Maturation of 5.8SrRNA is one of the most complicated forms of pre-ribosomal RNA processing, and two alternate pathways give rise to the short (5.8S_S_) and long forms (5.8S_L_) of 5.8SrRNA that differ by the presence of additional 7 or 8 nucleotides at the 5′ end^7^,^8^. The occurrence of two forms of 5.8S rRNA with slightly different 5′ ends is common in eukaryotes, even though the ratio between the two forms vary from one organism to another. The shorter form of 5.8S rRNA (5.8S_s_) represents 80% of the total^9^ and is generated by endonucleolytic cleavage at the A3 site of the internal transcribed spacer 1 (ITS1) by RNAse MRP and subsequent 5′ → 3′ exonucleolytic trimming of the 27S-A3 pre-rRNA down to site B_1S_ by Rat1 and XRN1^10^,^11^. The direct endonucleolytic cleavage gives rise to the longer 5.8S_L_ in eukaryotes and does not rely on the cleavage at A3 site^12^. The 3′ end of both variants is generated by cleavage at site C2 in the ITS2 by cofactors of the nuclear exosome, Rrp6^11^. In plants, the exact mechanism by which the long and short forms of 5.8S rRNA are produced is not fully known. Only Rat1/XRN2-like members have been reported in plants, and three XRN exoribonuclease homologs, XRN2, XRN3 and XRN4, were identified in *Arabidopsis*^13^. Among these, XRN2, XRN3 and XRN4 act as endogenous PTGS suppressors; XRN2 and XRN3 are nuclear exoribonucleases, while XRN4 localizes to the cytoplasm^14^. In *Arabidopsis*, it has been shown that AtXRN2, similar to yeast nuclear Rat1, is responsible for cleavage at the sites A2 and A3 in ITS1^15^. In several model systems, RNAse MRP (Mitochondrial RNA Processing) has also been reported to participate in the cleavage of the internal transcribed spacer 1 (ITS1) of the rRNA precursor at the specific site A3, producing the mature 5′ end of the 5.8S rRNA^16^. Land plants do not encode any recognizable genes for RNAse P enzymes or proteins specific for ribonucleoprotein RNase P^17^. The protein-only RNase P enzymes (PRORPs), which are present in the organelles and the nucleus, have entirely replaced the ribonucleoproteins to exert RNAse P functions in plants^18^. AtMTR4, an exosome cofactor in the nucleolus, cooperates in several steps of 5.8S rRNA maturation and the removal of rRNA maturation by-products^19^. HEN 2, a plant specific RNA helicase that is absent in other eukaryotic lineages, targets different subsets of nuclear RNA substrates for degradation by the exosome^20^.

Our study demonstrated the existence of highly expressed, unique small RNAs generated from the 5′ terminus of 5.8SrRNA in black pepper, a unique basal angiosperm plant, as well as in other representative plant species ranging from green algae to eudicots. Until now, very little has been known about functional small RNAs from the 5.8S ribosomal RNAs in plants. Such a lineage specific small RNAs can be a novel candidate for use in molecular systematics of plants.

## Results

### Identification and analysis of unique ribosomal small RNAs from black pepper

**Figure 2:**
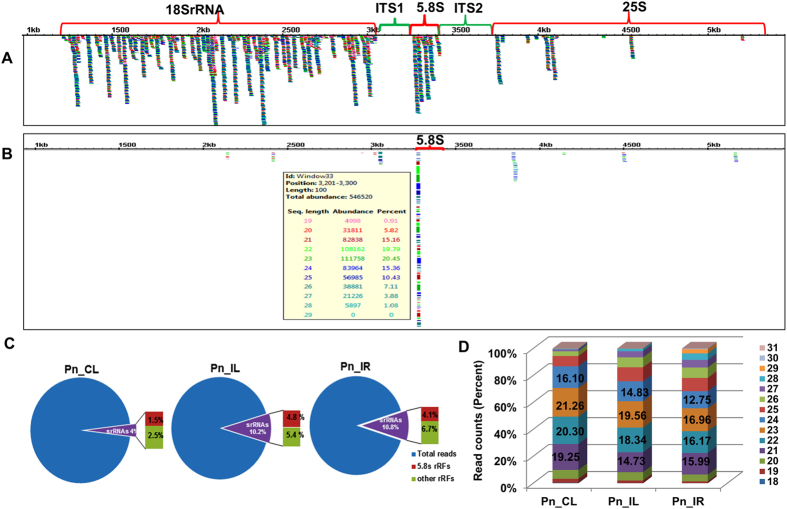
Distribution and abundance pattern of ribosomal RNA derived small RNAs in *Piper nigrum* small RNA libraries (**A**) Distribution of unique srRNA candidates against black pepper rRNA. (**B**) Abundant srRNA from Pn_CL (upto100 rpm) mapped against rRNA. Most of the abundant reads were mapped at the 5.8S rRNA region. (**C**) The proportion of 5.8S rRFs in the Pn_CL, Pn_IL and Pn_IR libraries. (**D**) The length categorisation of 5.8S rRFs among the three libraries.

**Figure 3:**
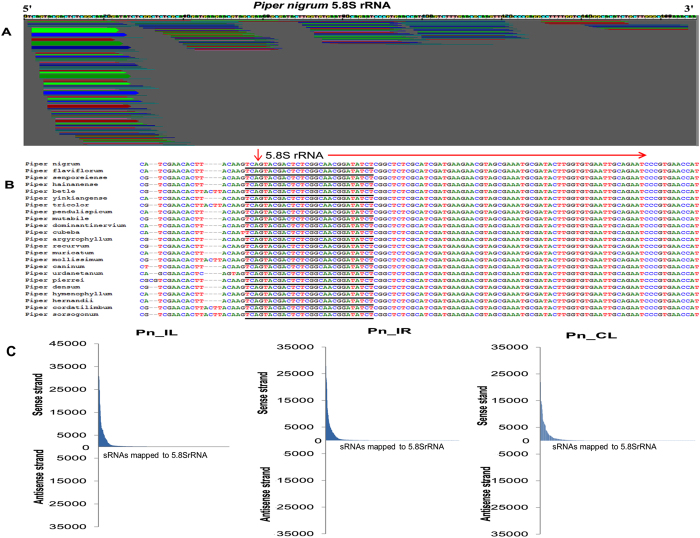
Abundance analysis of 5.8S rRFs. (**A**) The 5′5.8S rRFs were the most abundant category of srRNAs in *Piper nigrum*. srRNAs with read numbers > 10 were analyzed. (**B**) The 5′ conserved sequences from different species of *Piper.* (**C**) The mapped srRNAs to 5.8S rRNA.

The high throughput small RNA sequence data of control leaf (Pn_CL), *P. capsici* infected leaf (Pn_IL) and infected root (Pn_IR) of *Piper nigrum* were used for the analysis of sRNAs originating from ribosomal RNAs. Among the Pn_CL, Pn_IL and Pn_IL sRNA libraries of *Piper nigrum*, 750,269 (4.0%), 1,288,184 (10.2%) and 1,401,971(10.8%) reads, respectively, were mapped to rRNA (Table 1). The mapped unique srRNA candidates were distributed throughout the rRNA transcript, and the small RNA reads that mapped to internal transcribed spacers (ITS1 and ITS2) were negligible (Fig. 2A). The abundance analysis of srRNAs (>100 RPM) revealed that most of the prominent srRNAs clustered around the 5.8SrRNA region (Fig. 2B). Among the total srRNAs of all the three libraries, an average of 40.94 percent of the sequences could be categorized as 5.8S rRNA-derived small RNAs (Fig. 2C), of which the largest subset were sRNAs of 23 nt in length (Fig. 2D). The size distribution of srRNAs showed that 21 nt was the most common length of sRNAs in the Pn_CL and Pn_IR sRNA libraries, whereas the largest percentage of reads in the Pn_IL library were 23 nt long (Supplementary Figure 1). Because the sRNAome might also contain a large portion of rRNA degradation products, it was difficult to identify the functional srRNA from the large dataset of small RNAs. Generally, rRNA reads were neglected in the sRNA characterization and were assumed to be degradation products. The RIN values > 8.0 and 28S:18S ratio > 1.5 of the samples indicated a high degree of RNA integrity, thus excluding the possibility of extensive degradation (Supplementary Figure 2). The small RNAs that mapped to the 5′ terminus of 5.8SrRNA were found to be extremely abundant (Fig. 3A), and many of the variants contained 2–3 upstream nucleotides (Table 2). The proportion of small RNAs from the internal and 3′ terminal regions of 5.8SrRNA was much lower than the proportion of sRNAs from the 5′ end (Fig. 3A). 5.8S rRNA with few upstream nucleotides may potentially indicate the presence of 5.8S_L_rRNA. Notably, the 5′ sequences of 5.8S rRNA near ITS1 are highly conserved among the different species of Piperaceae family (Fig. 3B). Also, a major share (>99%) of srRNAs were mapped at the 5′ end of the sense strand of 5.8S rRNA (Fig. 3C). The positional distribution and high abundance of 5.8S rRFs indicated that sRNAs are generated precisely from the 5.8S rRNA.

**Figure 4:**
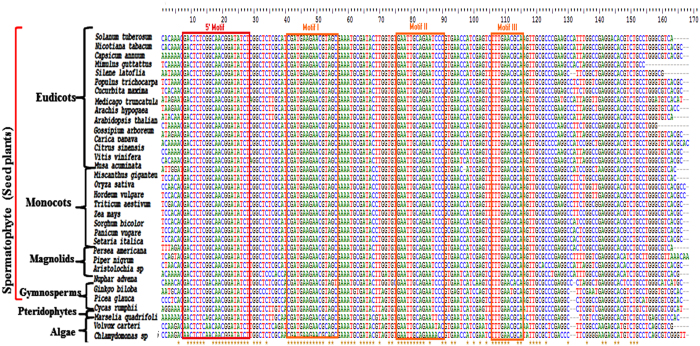
Multiple sequence alignment of rRNAs from different plant species. GACTCTCGGCAACGGATAT is the 5′5.8S rRF mapped 5′ motif found in all the lineages of spermatophyte plants.

**Figure 5:**
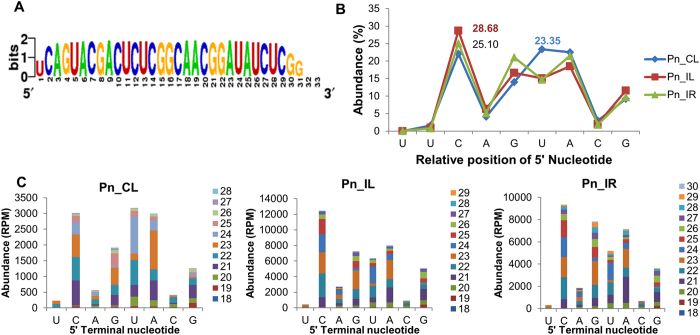
Analysis of isoforms and cleavage pattern of 5′5.8S rRFs from black pepper (**A**) Sequence logo generated from the different isoforms of 5′5.8S rRFs. (**B**) The cleavage pattern of 5.8S rRFs assessed upstream of the GACUCUC consensus (**C**) The length distribution of 5′5.8S rRFs.

Apart from the three conserved motifs previously described in viridiplants (Fig. 4), GACUCUCGGCAACGGAUAUCUC was observed as the novel 5′ consensus sequence present in all the seed plants (Spermatophytes). The 5′5.8S rRNA of different *Piper* species possessed a unique seven nucleotide sequence, UCAGUAC, upstream of the 5.8S rRNA consensus sequence GACUCUCGGCAACGGAUAUCUC. The different 5′ length variants of the 5.8S srRNAs from the *P. nigrum* library were represented as sequence logo (Fig. 5A). An analysis of the 5′ terminal nucleotides showed that cytosine was the terminal nucleotide in 25–28% of the 5′5.8S rRF sequences in the Pn_IL and Pn_IR sRNA libraries, indicating major cleavage at C (U↓CAGUACG). In contrast, major cleavage (23.35%) was observed at the U position in the Pn_CL library (U↓CAG↓UACG) (Fig. 5B). The length distribution pattern of 5′5.8S rRFs with the C terminal nucleotide showed that the major distribution of the small RNA variants ranged from 21 to 25 nt in size (Fig. 5C).

### Signature small RNAs in diverse plant lineages

**Figure 6:**
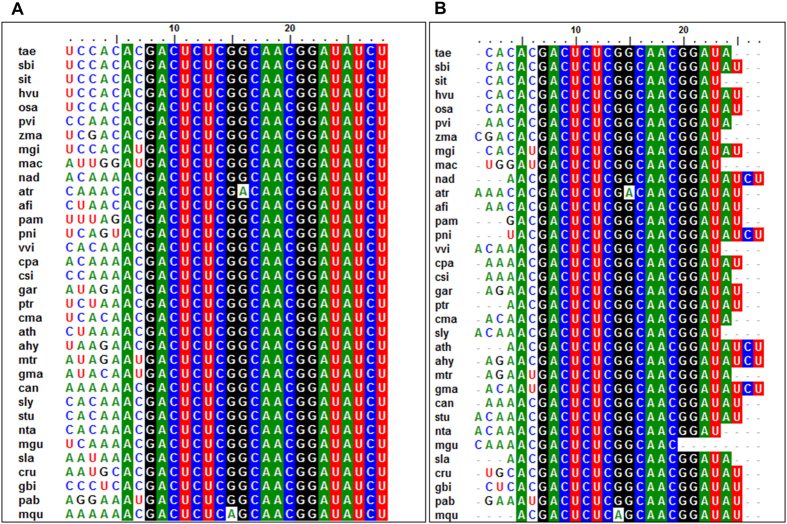
Unique sequence variation at the 5′ ends of 5.8S rRNAs and the prominent 5.8S rRFs in plants. (**A**) Sequence diversity at the 5′ termini of 5.8S rRNA in different plant species (**B**) Multiple sequence alignment of 5′5.8S rRF from different plant lineages. The most predominant 5′5.8S rRFs from each plant species were aligned using ClustalW. (tae: *T.aestivum*, sbi: *S. bicolor*, sit: *S. italica*, hvu: *H. vulgare*; osa: *O. sativa*; pvi: *P. virgatum*, zma: *Z. mays,* mgi: *M. giganteus,* mac: *M. acuminata,* nad: *N. advena,* atr: *A. trichopoda,* afi: *A. fimbriata,* pam: *P. americana*, pni: *P. nigrum*, vvi: *V. vinifera*, cpa: *C. papaya*, csi: *C. sinensis*, gar: *G. arboretum*, ptr: *P. trichocarpa*, cma: *C. maxima*, ath: *A. thaliana*, ahy: *A. hypogaea*, mtr: *M. truncatula*, gma:*G. max*, can: *C. annuum*, sly: *S. lycopersicum*, stu: *Solanum tuberosum*, nta: *N.tabacum*, mgu: *M. guttattus*, sla: *S. latifolia*, cru: *C. rumphiis*, gbi: *G. biloba,* pab: *P. abies*, mqu: *Marsilea quadrifolia.*

The signature srRNAs were detected in different lineages of plants by analyzing their high throughput small RNA sequence datasets (Supplementary Figure 3A–E). A close examination of the 5′ end of 5.8SrRNA revealed very distinct sequence diversity among different plant lineages (Fig. 6A), and the most abundant rRFs from the small RNA libraries mapped to 5.8S rRNA, especially at the 5′ end (Fig. 6B). Furthermore, these small RNAs contained the conserved 5′ motif. Therefore, it is possible that the cleavage at the 5′ end of the 5.8S rRNA is a conserved mechanism of small RNA biogenesis in plants. An analysis of the small RNAome confirmed that the precise cleavage of unique rRF signature small RNAs were prevalent across diverse plant lineages. The monotypic genus *Amborella* possessed a single nucleotide polymorphism at the consensus motif, GACUCUCGACAACGGAUAUC, whereas in the Pteridophyte *Marselia quadrifolia*, a G to A transition in the 8^th^ position of the consensus sequence GACUCUCAGCAACGGAUAU was observed (Table 3, Fig. 6A). Among the monocot group, the consensus sequence of CACGACUCUCGGCAACGGAUAU was observed in all the plants belonging to the order Poales except *Miscanthus sp*, which showed a C to U transition at the third nucleotide position. Within the Poacea family, the closely related plants *Oryza sativa* (Sub family Ehrhartoideae), *Hordeum vulgare* (Pooideae), *Triticum aestivum* (Pooideae), and *Setaria italica* (panicoideae) shared the distinct 5′ sequence of CACAC. In *Zea mays*, a transversion mutation resulted in CGACAC at the 5′, and in *Panicum virgatum*, AACAC was the 5′ terminal nucleotide sequence. *Musa accuminata*, a monocot plant belonging to the Zingiberales order, exhibited a very distinct 5′ nucleotide sequence, UUGGAU, which was terminal to the consensus sequence GACUCUCGGCAACGGAUAU in the rRFs. Even though the rRF consensus sequence CACGACUCUCGGCAACGGAUAU observed in the Poacea plants was present in *Cycas rumphii* and *Ginko biloba*, they both possessed distinct 5′ terminal nucleotides in the 5.8S rRFs, such as AUGCAC and CCUCAC. AACGACUCUCGGCAACGGAUAU was identified as the consensus sequence in all the dicot plants studied except the fabid plants *Medicago truncatula* and *Glycine max*, in which a C to U transition was observed in the 3^rd^ nucleotide base. However, each plant species possessed a unique patch of 5.8S rRNA; these signatures were present as abundant 5.8SrRNA in the sRNAome. The three plant species studied from the magnoliid clade had the consensus sequence of ACGACUCUCGGCAACGGAUAUCU, and the plants belonging to each family possessed distinct 5′ sequences in the unique 5.8SrRFs, such as CAGU in *Piper nigrum* (Piperaceae), UAAC in *Aristolochia fimbriata* (Aristolochiaceae), and UUAG in *Persea americana* (Lauraceae). Even though a similar consensus sequence was identified in the closely related basal angiosperm plant *Nuphar advena*, it possessed the well-defined 5′ sequence CAAA. Despite the nucleotide variation present in the 5′ consensus sequence, *Amborella sp.* had AAAC nucleotides upstream of the consensus sequence.

Small RNA libraries of lower plants also possessed the rRF signature small RNAs with similar processing and cleavage events, but with lower abundances. We identified unique 5.8SrRF signature sequences from the *Chara sp.*, a green algae species among the most closely related to land plants. The distinct nucleotide variations of the 5.8SrRF signatures and their low expression levels compared with the land plants were detected in *Chara sp*. Meanwhile, signature small RNAs were also detected in green algae such as *Chlamydomonas reinhardtii* and *Volvox carteri*, which represent the oldest ancestors of plants, but the read counts were below detectable levels (RPM < 10).

### Analysis of length distribution of the 5′5.8S rRFs and the 5′ cleavage patterns

To determine whether any unique cleavage and length patterns were present in each species, we analyzed the sRNA datasets from different tissues. An analysis of 5.8S srRNAs from different sRNA libraries of the same plant showed similar cleavage patterns in all the plant tissues (Supplementary Figure 4A–E). In a majority of the plant species, the exact 5′ nucleotide sequences of 5.8S rRNA were not clear from the database sequences. Therefore, the 5′ cleavage patterns of the rRF sequences were studied from the GACUCUC consensus sequence. The major cleavage was observed in the C nucleotide in all species belonging to the Poaceae family except *Panicum virgatum,* in which major cleavage was observed at the ‘A’ nucleotide (Supplementary Figure 4C). Other monocot orders, such as Zingiberales, showed a very distinct cleavage pattern at the U nucleotide in all the *Musa accuminata* sRNA libraries. We also observed that the sRNAs cleaved from these unique 5.8S rRNA loci were predominantly 23 to 25 nt in length, and the major cleavage event occurred at the 3^rd^ nucleotide upstream of the GACUCUC consensus sequence in most of the monocot plants. The major cleavage was mapped to the 3^rd^ and 6^th^ C nucleotide upstream of the consensus sequence in *Triticum aestivum* and *Zea mays*, respectively. By contrast, in both sRNA libraries of the Pteridophytic plant *Marselia sp.,* the major cleavage was mapped to the 2^nd^ nucleotide upstream of the GACUCUC consensus, in which the 5′5.8S rRFs were predominantly 21 nt in length (Supplementary Figures 4A & 5E).

Interestingly, in all the dicot plants except *Mimulus guttatus*, major cleavage was observed at the ‘A’ nucleotide upstream of the consensus sequence (Supplementary Figure 4B). Among the core dicots, such as Vitales and Malvids, the most prevalent cleavage was mapped to the 6^th^ and 5^th^ nucleotides upstream of the GACUCUC consensus, respectively. The cleavage at the 5^th^ nucleotide was also observed in Cucurbitales and Fabales of the Fabids group, whereas in *Populus trichocarpa* (Malpighiales), the major cleavage was mapped to the 3^rd^ nucleotide upstream of the consensus sequence. The cleavage at the 6^th^ nucleotide was predominant in all the Solanales plants. The length categorization of small RNAs from each cleavage position upstream of the GACUCUC consensus sequence showed that the small RNAs produced at the major cleavage site were predominantly 23–24nt in length (Supplementary Figure 5A–E). The 5′5.8S rRFs constituted an average of approximately 4.33 percent of the total reads in the small RNA libraries (Supplementary Figure 6). Relative occurrence of the most prominent 5′5.8SrRF, ‘AACGACUCUCGGCAACGGAUAUCU’ from the small RNA datasets of Arabidopsis next gen sequence database (https://mpss.danforthcenter.org) revealed a read count greater than 100 RPM from eighty nine sRNA libraries (Supplementary Figure 7). While thirteen sRNA libraries, that represents the 8.5% of the total libraries had shown the read count (RPM) less than 10. This shows the higher occurrence of the 5′5.8S srRNAs in the high throughput sRNA datasets.

### Analysis of the association between 5′5.8S rRFs and the Argonaute complexes

**Figure 7:**
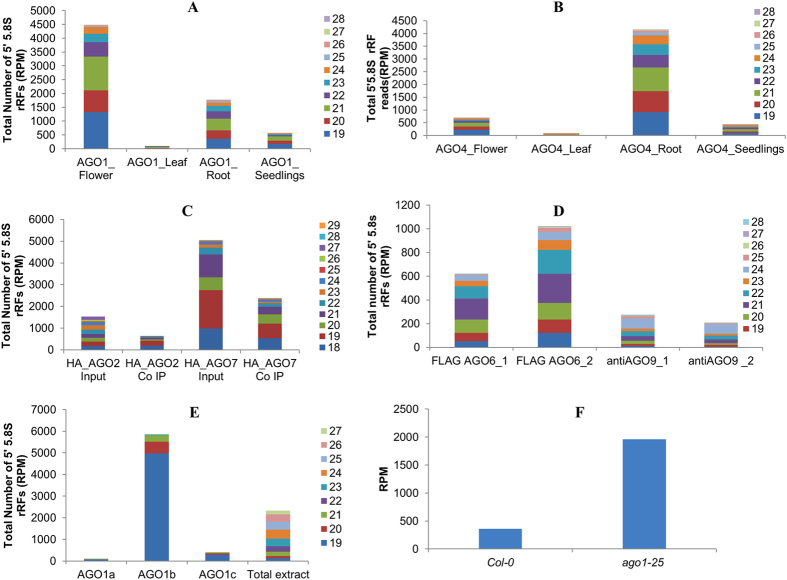
Association of 5′5.8S rRFs with different Argonaute proteins. 5′5.8S rRFs identified from AGO immuno-precipitated sRNA libraries of *A. thaliana.* (**A**) AGO1 (**B**) AGO4 (**C**) AGO2 & AGO7 (**D**) AGO6 & AGO9 (**E**) 5′5.8S rRFs identified from different homologs of AGO1 immuno-precipitated sRNA libraries of *O. sativa.* The sRNA data (GSM707682, GSM707686, GSM304284, GSM415789 and GSM415791)^22^,^23^,^39^ from the studies of Wang *et al*., 2011, Montgomery *et al*., 2008 and Havecker *et al*., 2010 were analysed and the total read counts of 5′5.8S rRFs was represented as the normalized read count (RPM) from the corresponding sRNA library. (**F**) 5′5.8S rRFs detected from the *Ago1–5* mutants.

In order to study the potential role of rRFs in the RNAi silencing pathways, their occurrence in the AGO complexed sRNAome of model plant species such as *Arabidopsis thaliana* and *Oryza sativa* was analyzed. It was proposed that each AGOs have distinct binding affinities towards the small RNAs with different 5′ terminal nucleotides^21^. The functional diversification of ten different AGOs was deduced from the associated sRNAs and genetic analysis in Arabidopsis^22^. AGO1 was reported to associate with 21-nucleotide sRNAs with a 5′ U, AGO2 binds 21 and 22 nucleotides with a 5′ A, while AGO4 predominantly associates with 24-nucleotide sRNAs with a 5′ A^21^,^22^,^23^. While AGO7 mainly binds MIR390, an initiator of tasiRNA production; AGO4 binds repeat and heterochromatin associated small RNAs. The association of tRNA derived RNA fragments with AGO proteins were also reported in plants^24^. The sequence data of argonaute coimmuno-precipitated sRNAome of Arabidopsis reported previously by Wang *et al*., 2011, Montgomery *et al*., 2008 and Havecker *et al*., 2010 were analyzed to identify the association of rRF candidates to AGOs. The 5′5.8S rRF variants were detected in the sRNAome that co-immunoprecipitated with different argonaute complexes, such as AGO1 to AGO9 (Fig. 7). The 5′5.8S rRFs displayed a strong preference for AGO1 in *Arabidopsis thaliana* (Fig. 7A). By comparing the total normalized reads among the AGO1 precipitated small RNA libraries from different tissues of *Arabidopsis*, 5′5.8S rRFs were found to be most prominent in the flower sRNA library, in which the relative abundance of FLAG AGO1 protein was high. In contrast, the AGO4 associated 5′5.8S rRFs were highly abundant in the root tissue, which has a higher relative FLAG AGO expression^25^. The abundances of 5.8S rRFs associated with AGO1 or AGO4 were very low in the leaf compared to other tissues. The length distribution of 5′5.8S rRFs also showed variations in the different AGO complexed sRNA libraries. The AGO4 sRNA datasets showed that the 5′5.8S rRFs were predominantly of 21 nt in length (Fig. 7B). In the AGO1, AGO2 and AGO7 complexed sRNA libraries, the 5′5.8S rRFs were predominantly 19 nt in length (Fig. 7C), while the 5′5.8S rRFs of 21 and 24 nt in length constituted the majority of the 5′5.8S rRFs in the AGO6 and AGO9 sRNA libraries, respectively (Fig. 7D). The relative abundance of normalized read counts of 5′5.8S rRFs among the different AGO complexed sRNA library from the Arabidopsis flower showed their high occurrence in the AGO1 sRNA library (Supplementary Figure 8).

When we compared the 5.8S rRFs associated with different components of Argonaute 1, such as AGO1a, AGO1b, and AGO1c, in the AGO1 knock-down *Oryza sativa* seedlings, the 19nt 5′5.8S rRFs showed increased accumulation; this is similar to observations in *Arabidopsis,* and among the three AGO1 complexes, AGO1b predominantly bound the 19 nt 5′5.8S rRFs (Fig. 7E). The detailed analysis of sRNA libraries of Arabidopsis *Ago1–25* mutant (GSM343002, GSM343003, GSM343004) revealed the high expression of 5′5.8S rRFs compared with the control plants (Fig. 7F). This suggests the functional specification of 5′5.8S rRFs according to their interactions with different Argonautes as well as their biological roles; namely, 5′5.8S rRFs probably play a similar role as microRNAs in the gene regulatory mechanisms of different physiological processes.

### Do rRFs have a non-canonical miRNA-like precursor?

**Figure 8:**
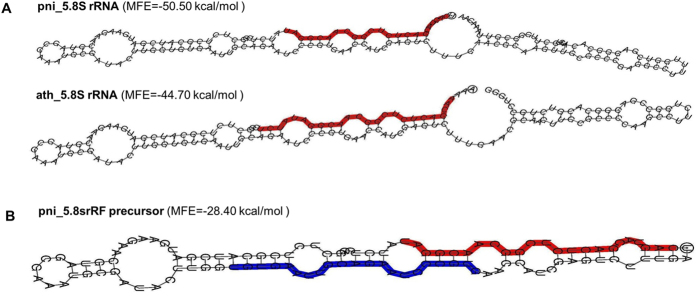
The predicted secondary structure of 5.8SrRNA of black pepper. The secondary structure of (**A**) *P. nigrum* and (**B**) *A. thaliana* 5.8S rRNA as predicted from the RNAFold. (**C**) The non-canonical short hairpin precursor of 5′5.8S rRFs. The 5′5.8S rRF was highlighted in red color. The next abundant rRF identified from the sRNA library was highlighted in blue in the opposite strand.

We assessed the ability of the 5.8S rRNA region to form non-canonical microRNA-like stem loop precursor structures. The *in silico* analysis predicted that the secondary structure of the *P. nigrum* 5.8S rRNA consisted of two hairpins joined by a hinge, with an MFE value of −50.50 kcal/mol (Fig. 8A). A similar hairpin structure was predicted for the 5.8S rRNA from *Arabidopsis thaliana*, with an MFE value of −44.70 kcal/mol. An alternate, non-canonical miRNA-like short hairpin precursor was also predicted, which had a sequence length of 110 nucleotides and an MFE value of −28.40 kcal/mol (Fig. 8B). 5.8S rRF could be mapped to the stem region of this short hairpin. Our results also indicated the second most abundant class of small RNAs from 5.8S rRNA that could be mapped to the same precursor structure. Furthermore, the 5′ modified RLM RACE experiments revealed cleavage at the C nucleotide (Supplementary Figure 9), which was identified as the major cleavage position of 5′5.8S rRFs during pathogenic infection. Thus, it is possible that the precursor 5.8S rRNA located 6 or 7 nucleotides upstream of the GACUCUC consensus sequence was expressed during the processing of 5.8SrRNA in pathogen-stressed black pepper plants. Similar non-canonical short hairpin structures with MFE values within the range of −28.5 KCal/mol were predicted for most of the plants, even if their 5′ ends were different (Supplementary Figure 10).

### Analysis for the Dicer Dependency of 5.8S rRFs

**Figure 9:**
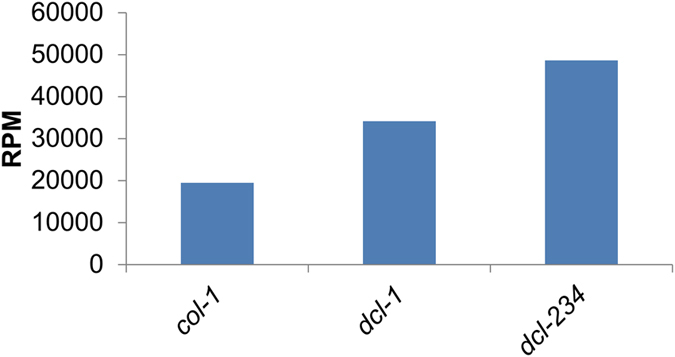
Involvement of Dicer-like activity on the biogenesis of srRNAs. The expression of 5′5.8S rRFs detected from the *dcl-1 and dcl-234*mutant Arabidopsis plants.

The sRNA datasets of flower tissues from the partial-loss-of-function *dcl-1* mutant and *dcl-234* triple mutants (GSE44622) showed high expression of 5.8S rRF in the mutant as compared to the wild type plants (Fig. 9). Notably, the 24 nt long 5′5.8S rRF ‘AACGACUCUCGGCAACGGAUAUCU’ showed high read count in all the sRNA libraries of *dcl-1, dcl-234* mutant compared to Col-1 wild type plants. The expression in the *dcl-234* triple mutant plant suggests the enrichment of 5.8S rRFs, similar to miRNAs.

### The potential functional role of 5′5.8S rRFs

**Figure 10:**
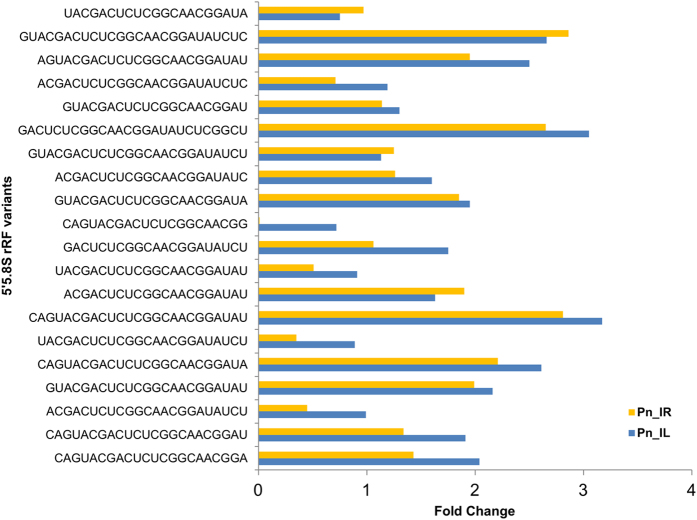
The expression of 5′5.8S rRF variants in the Pn_IL and Pn_IR libraries compared to the control leaf library (Pn_CL).

**Figure 11:**
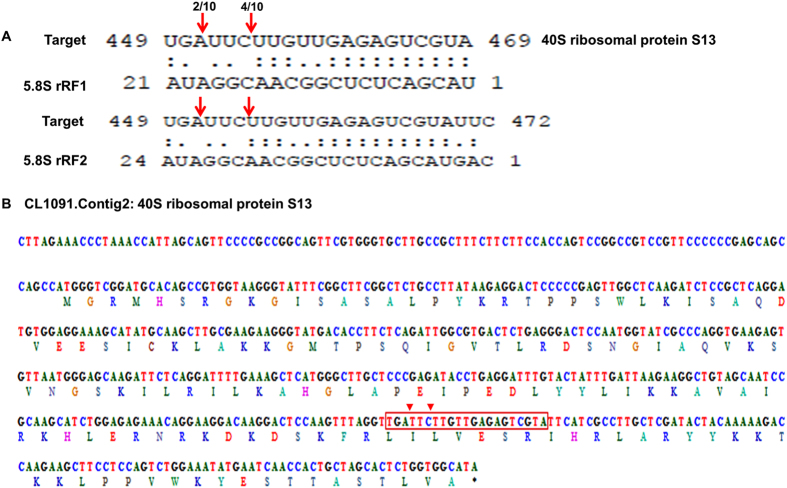
Experimental mapping of 5′5.8S rRF mediated cleavage on the predicted target. (**A**) Cleavage was mapped on the predicted rRF binding site in the mRNA transcript encoding 40S ribosomal protein S13 from black pepper by modified 5′RLM RACE experiments. The red arrow indicates mapped cleavage sites and the number indicates the frequency of clones from RACE experiments (**B**) 5′5.8S rRF cleavage sites on the CL1091.Contig 2 coding for 40S ribosomal protein S13.

The preferential breakdown of 5′5.8S rRFs and their prominence in the plant system suggests their stability and functional relevance. The variations in the expression of twenty abundant 5′5.8S srRNAs in the leaf and root sRNA libraries of *P. capsici* infected *Piper nigrum* plants (Fig. 10) showed the higher expression of many of the rRF variants. Because these small RNAs may potentially function as microRNAs, targets of the abundant 5′5.8S rRFs were predicted from black pepper mRNA transcripts using psRNATarget by following the miRNA target criteria. Genes encoding the 40S ribosomal protein, adenosylhomocysteinase, diaminopimelate epimerase, shikimate O-hydroxycinnamoyltransferase, diphthamide synthase and cytochrome P450 were the predicted targets of the 5′5.8S rRF variants from black pepper (Table 4). Even though mRNA cleavage and translational repression were predicted as the target regulatory mechanisms, cleavage was identified as the most prominent mechanism among the predicted targets. Potential target mRNAs were also predicted from other plants, such as *Arabidopsis thaliana, Oryza sativa, Carica papaya, Citrus sinensis, Cucumis sativus, Gossipium arboreum, Glycine max, Medicago truncatula, Populus trichocarpa, S. tuberosum, S. lycopersicum* and *Nicotiana tabaccum*. Transposable element genes, mRNAs encoding RNI-like proteins, xyloglucan endotransglucosylase, and several other functionally relevant proteins were predicted as the putative 5′5.8S rRF targets in *Arabidopsis thaliana*, while homeobox domain containing proteins and S-adenosyl-L-methionine:benzoic acid/salicylic acid carboxyl methyltransferase were identified as the rRF targets in *Oryza sativa* (Supplementary Table 1). Adenosylhomocysteinase, the competitive inhibitor of S-adenosylmethionine (SAM)-dependent methyl transferase reactions, was identified as a putative common target in the monocot plants *Hordeum vulgare, Triticum aestivum* and *Sorghum bicolor*. SAM methyl transferase and adenosyl homocysteinase are involved in methionine synthesis and methylation recycling in plants. In many plant species, proteins involved in rRNA biogenesis and ribosome assembly were predicted as 5′5.8S rRF targets. The 5′ modified RLM RACE experiments in black pepper further mapped the cleavage events to the 5′5.8S rRF aligned region of the transcript encoding 40S ribosomal protein S13, which is the RNA binding domain (Fig. 11). Four variants of RPS13 mRNA transcripts were identified from the assembled stress responsive transcriptome data from black pepper. The details of the FPKM normalized expression of the RPS13 mRNA isoforms from the *P. nigrum* transcriptome were shown in Supplementary Table 5. All the four variants of RPS13 mRNA transcripts showed down regulation in the pathogen infected tissues compared to the control (Supplementary Figure 11).

**Figure 12:**
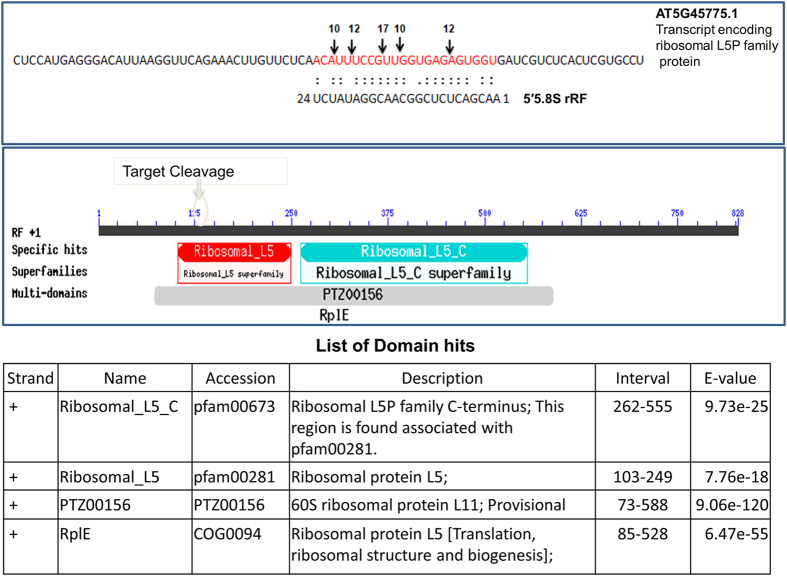
The 5.8S rRF mediated cleavage on the predicted targets detected from the Arabidopsis degradome data. The cleavage was detected on the rRF aligned position on the target mRNAs of ribosomal L5P family protein, at the L5 domain.

The sRNA mediated cleavage at the predicted target gene encoding ribosomal L5P family protein (AT5G45775.1) was detected from Arabidopsis degradome data (Fig. 12). The multiple cleavages (Supplementary Figure 12) were detected at the sRNA aligned region that encodes the conserved domain for the ribosomal protein L5. The degradome analysis also mapped the cleavage from other targets such as glutamate:glyoxylate aminotransferase (AT1G23310.2), N2,N2-dimethylguanosine tRNA methyltransferase (AT5G15810.1), myb domain protein (AT4G37260.1), N-terminal nucleophile aminohydrolases (AT5G61540.1) from Arabidopsis with comparatively less read count (<10) (Supplementary Figure 13). These findings demonstrated the precise processing of 5′5.8S rRFs and their potential functional role in gene regulation in plants.

## Discussion

The higher abundance of srRNAs that mapped to the 5′ terminus of 5.8S rRNA of *Piper nigrum* indicated that these srRNAs are precisely processed rather than randomly degraded. In a eukaryotic cell, ribosomal RNAs are prone to fast degradation, which eliminates rRNAs that were defective in length processing, folding and assembly^26^. The 5′ end of 5.8S rRNAs might be protected from degradation and may function as sites for biogenesis of functional sRNAs through unknown processing events. The length variation and differential density has been previously reported as a peculiar characteristic of rRNA-derived small RNAs^27^. In agreement with the findings from *Piper nigrum*, similar unique 5′5.8S srRNA signatures were detected from plants belonging to different phylogenetic groups. Even in well-studied model plants, these small RNAs remained hidden, as they generated from the rRNA. The deep evolutionary history of the plant can be assessed from the unique sRNAs that mapped to the syntenic rRNA regions in the genome, as these sRNAs may act in the ancestral gene regulatory apparatus for the biogenesis of evolutionarily labile sRNAs. The widespread, multi-laterally adapted RNAi system orchestrates several cellular processes and includes potentially lineage-specific variations in the sRNAome^28^. Thus, it would appear that the veracity and prevalence of these small RNAs were largely the consequence of evolutionary patterns the plant acquired through generations. Moreover, the accuracy of processing of these unique 5′5.8S srRNAs, as demonstrated from various plant sRNA libraries, validated their biogenesis through precise, endogenous cleavage from the nascent rRNA transcripts. The 5′ end of 5.8S rRNA is heterogeneous in eukaryotes. The terminal nucleotides in 5′5.8S rRFs indicated their biogenesis from the 5.8S_L_rRNA that differs by seven or eight nucleotides from other counterpart 5.8S_S_ rRNAs^8^. As is the case for many eukaryotes, the exact biological role of the two different forms of 5.8S rRNA was largely unknown in plants. It is possible that the long 5.8S rRNA (5.8S_L_), with 7 nt upstream sequences were processed extensively to produce signature small RNAs in plants. Previously, three conserved 5.8S rRNA motifs, CGATGAAGAACGyAGC (motifI), GAATTGCAGAAwyC (motif II), TTTGAAyGCA (motif III), were identified in Viridiplantae^29^. Sequence motifs in small RNAs can potentially regulate their stability and intracellular localization. The multiple sequence alignment of 5.8S_L_rRNA consisting of 6–7 nt upstream sequences and a LocARNA-P analysis confirmed GACUCUCGRCAACGGAUAUCU as the novel, functionally active, 5.8S rRNA motif present among the seed plants (spermatophytes) (Supplementary Figure 14). The abundant srRNAs spans this novel, conserved motif in all the lineages of the Viridiplantae. The lower plant groups, such as Pteridophytes and green algae, possessed distinct nucleotide variation in this consensus sequence. Because the sequence variations that contribute to the lineage differences are highly evident in the rRNA, the characterization of 5′5.8S rRFs is an important model to study the evolution of plants^30^. Within-family, the 5′5.8S rRFs were generally similar, while among the distant lineages, the differences in the 5′ nucleotides were apparent. Therefore, the study of the evolutionary dynamics of these unique sRNAs can give informative insights into their functional importance in plant development. As previously described^31^, the presence of all the three Viridiplantae motifs in the selected 5.8S rRNA and the ability to form a helix at the motif III (TTTGAAyGCA) indicated that no pseudogenes were involved in the dataset. The genomic locations of 5′5.8S rRFs were mapped to Chr2:5784–5808 (+strand) and Chr3:14199755–14199779 (+strand) in *Arabidopsis thaliana* and to Chr2: 28715492–28715515, Chr9: 4632–465 and Chr9: 12560–12583, 20488–20511, 28416–28439, 36344–36367 in *Oryza sativa*. Above all, the close examination of small RNAs mapped to the 5′ and 3′ ends of 5.8S rRNA could accurately define the termini of the 5.8S rRNAs from each species, which is difficult using other methods.

The regulatory structural RNAs were capable of forming a great variety of secondary structures, rendering them for a classical DICER processing system^32^. The non-canonical pathways involved in the biogenesis of small RNAs may have been predicted from the noncoding structural RNAs such as tRNAs^33^ and snoRNAs^1^. Moreover, recent research has demonstrated their biologically functional roles^33^,^34^,^35^ and genesis by a specific RNase under stress conditions^36^. The alternate secondary structure of 5.8S_L_ rRNA from *Piper nigrum* consisted of two hairpins and a hinge in the third motif (the TTTGAAyGCA region), which was similar to the previously reported novel box H/ACA in snoRNAs, in which small RNAs are formed from the conserved motifs box C, box D, and box H/ACA^1^. Similarly, the 5.8S srRNAs at the conserved motif III (TTTGA) can also form a non-canonical short hairpin structure. Interestingly, we observed that most of the plant 5.8S_L_ rRNAs could potentially form similar short hairpin structures even though sequence variation existed in the 5′ ends. The potential of forming secondary stem loop structures indicated that other than the conventional RNA processing by endonucelolytic and exonucleolytic cleavage, a possible non-canonical biogenesis pathway may be involved in their precise excision as microRNAs. Similar to tRNA genes, the 5.8S_L_ rRNA loci also has the intrinsic potential to form an alternative short hairpin structure, giving rise to mature RNAs or sRNAs. Apart from the usual rRNA recycling events for eliminating the degraded and defective rRNAs, the occurrence of functional srRNAs in the sRNAome of an organism has also been reported^27^. Therefore, many or some of the core pathways for producing miRNAs may function in the biogenesis of these small RNAs.

The abundant srRNAs identified from all the plant species were mapped to the 5′ end of the 5.8S rRNA. Even though the number of isoforms of 5′5.8S srRNAs was very large, the cleavage patterns were identical in different tissues of the same plant. The major cleavage events at the 5′ ends of 5.8S rRNAs were similar within a single plant group. The sequence artifacts that constituted the substitution variants, which differed by one or few nucleotides and were expressed at low levels, were excluded from the datasets. Regulatory RNAs are usually influenced by environmental signals and have potential functional roles during the stress response in plants. The normalization of each srRNA variant allowed for a more accurate estimation of their relative abundance. During pathogen-induced stress responses in black pepper, the major cleavage events were shifted by a few nucleotides upstream compared to the control leaf. The alteration of the 5′ end sequences can have a strong effect on its function and can potentially target an entirely different mRNAs during the stress response. A similar shift in the nucleotide cleavage site during the stress response was observed in *Hordeum vulgare* under pathogen infection and *Panicum virgatum* under drought compared to control tissues. It has also been reported that the rRNA-derived small RNAs are highly expressed during oxidative^37^ and heat stress^38^. The presence of the unique 5′5.8S rRFs and their association with different AGO proteins were identified in the AGO-associated sRNA profiles from *Arabidopsis thaliana*^23^,^39^ and *Oryza sativa*^39^. AGO1 harbors miRNA or sRNA, with a 5′ terminal uridine nucleotide, and change in the 5′ nucleotide redirects the miRNA or sRNA to a different argonaute complex^40^,^41^. Most of the 5′5.8S rRFs from plants do not possess the typical U nucleotide at the 5′ end, and *Arabidopsis* and Rice 5′5.8S rRFs detected from the AGO1 complexes raise the question of whether AGO1 has a preference for these unique small RNAs. Whereas the high expression of 5′5.8S rRFs were detected from the inflorescence of *ago 1–25* mutant, the fertile mutants with hypomorphic *ago1* alleles compared with the wild type plants. It may suggest the biogenesis of 5′5.8S rRFs not affected by the single nucleotide transition in the 3′ portion of the gene leading to the Gly-758 → Ser (19–3) substitutions^25^. The precisely processed, stable srRNAs and their occurrence in the AGO protein complexes further suggested that these srRNAs function like microRNAs or siRNAs in post-transcriptional gene silencing events.

An in-depth analysis of small RNA libraries from the flower tissues of *dcl-1, dcl-234* mutants, and (Col-0) wild-type plants revealed the abundance of 5′5.8S rRFs. The expression of 5.8S srRNAs was found high in.*dcl-234* triple mutant compared with the *dcl-1* mutant and wild type plants. The *dcl1*mutant is known to amplify the proportion of siRNAs and reduce the expression of miRNAs in the library^42^; whereas *dcl-234* triple mutant is known to reduce the proportion of siRNAs and significantly enriches the miRNA population^43^. Consistent with previous reports^44^, the high expression of the small RNAs might be due to the known residual activity and the insensitivity to certain miRNAs in the partial-loss-of-function *dcl1–7* mutant allele.

SAM-dependent carboxyl methyltransferase was identified as a potential target of 5′5.8S rRNAs in rice. S-Adenosyl methionine is the universal biological cofactor found in all branches of life, and it plays critical roles in the methylation of various biomolecules such as DNA, proteins and small molecule secondary metabolites. Methyl transferases, which are the largest group of SAM-dependent enzymes^45^ in plants, have key roles in stress responses and in primary and secondary metabolism^46^. Adenosyl homocysteinase acts by regulating DNA methylation, which affects the expression of certain genes involved in seed dormancy^47^. Certain common targets, such as genes involved in methionine biosynthesis and recycling, were predicted in the monocot plants, whereas the genes involved in ribosome biosynthesis, such as the 40S ribosomal protein, were identified as common targets among the dicot plants. Modified 5′RLM RACE experiments mapped the cleavage site to the highly conserved RPS13 domain on the predicted target mRNAs of the 40S ribosomal protein in black pepper. S13, which has been reported to form part of the amino acyl tRNA binding domain in the ribosome, also functions along with other ribosomal proteins to form ribonucleoprotein complexes with the 5.8S ribosomal RNA^48^. In plants, multiple expressed copies of the gene for the ribosomal protein S13 exist; these transcripts have been proposed to act through the gene dosage model^49^, as their proteins are required for optimum growth during different stages of the plant life cycle. It is possible that the unique ribosomal small RNAs may function in the gene dosage mechanisms that precisely regulate the expression of RPS13 in the different cell types and in the transitions between different developmental stages in plants. Also, analysis on Arabidopsis degradome data revealed the sRNA mediated cleavage on the predicted target mRNA encoding ribosomal L5P protein. The cleavage sites mapped at the sRNA aligned target region indicates the potential functional role of 5′5.8S rRFs in plants. In conclusion, our results showed that 5′5.8S rRFs are the unique, lineage-specific small RNAs with critical functional roles in plants.

## Materials and Methods

### Library preparation and Bioinformatic analysis of small RNAs from *Piper nigrum*

In this study, two high throughput sRNA library were prepared from leaf and root from *Phytophthora capsici* infected black pepper plants, and analyzed along with the previously sequenced control uninfected leaf sRNA library of black pepper^50^. The pathogen infection was artificially given to the plants using mycelia disc of virulent *P. capsici*, as mentioned in ref. 51. The total RNA enriched with the small RNA fraction (<200 nt) was extracted from the leaves and roots of *P. capsici* infected black pepper plants at 24 hour after pathogen infection, using mirVana miRNA isolation kit (Ambion) according to the manufacturer’s instructions. After the quality and quantity analysis of RNA using NanoDrop^TM^ 1000 spectrophotometer (Thermo Scientific, Wilmington, DE) and Agilent Bioanalyzer, the library of sRNAs (~18–30 nt) was constructed and sequenced^52^ on an Illumina Genome Analyzer at Beijing Genomics Institute (Shenzhen, Guangdong Province, China). The raw reads were processed to remove the low quality reads, and clean reads ranging from 18–30 nt length were selected. The small RNAs derived from rRNA, tRNA, snoRNA etc. were annotated from the black pepper small RNAome using BGI small RNA analysis pipeline procedure that analyze the known non-coding RNAs deposited in the Rfam and NCBI GenBank databases. The rRNA mapped reads were selected from the entire three libraries and analyzed further. The relative position of srRNAs was determined by mapping the reads to *P. nigrum* ribosomal RNA using PatMaN in VisSR Tool of UEA small RNA workbench (srna-workbench.cmp.uea.ac.uk)^53^. The full-length, perfect matches were accepted as hits. The rRNA-derived small RNAs (srRNAs) from all the three libraries were further analyzed for their read frequencies, size distribution and 5′ terminal nucleotide preferences. The srRNAs reads were normalized by dividing hit counts by the number of redundant reads in corresponding sRNA library and srRNAs with read numbers > 10 were selected, and their distributions and frequencies within the black pepper rRNA were analyzed. The cleavage pattern of the terminal nucleotides of the 5′5.8S rRFs were assessed upstream of the GACUCU consensus sequence. The individual read counts of twenty abundant 5′5.8S rRF variants were normalized (RPM) and their expression levels were compared to the expression levels of these variants in the control leaf sRNA library. The sRNA profiles of black pepper were deposited to the NCBI GEO database under the accession numbers GSM1606153 (Pn_CL), GSM1606154 (Pn_IL) and GSM1606155 (Pn_IR).

### Comparative analysis of sRNA datasets from diverse plant groups

The high throughput small RNA datasets of plants belonging to different lineages, including green algae (Chlorophyta), vascular (angiosperms and gymnosperms) and nonvascular plants (Pteridophyta), that were generated by Chavez Montes *et al*.^54^ were downloaded from the NCBI gene expression omnibus. The study group consisted of a total of 97 small RNA library datasets from thirty-seven distinct plant species^21^,^55^. Brief information about the sRNA datasets used in the study is provided in Supplementary Table 2. The sequence accession numbers of ribosomal RNAs from different plant species are given in Supplementary Table 3. The predominant group of 5.8S rRNA-derived small RNAs from most of the plant species possessed few upstream nucleotides compared to the database-curated 5.8S rRNAs. We selected a sequence boundary located 7 nt upstream of the GACUCUC motif in the 5.8S rRNA of all the plant species, and the consensus secondary structure was analyzed using locARNA-P, a software package that computes STARS (the structure based alignment reliabilities)^56^. The abundance and the distribution of 5′5.8S rRNAs were analyzed using the sequence alignment and VisSR tools in the UEA sRNA workbench. The 5′ cleavage pattern of 5′5.8S srRNAs were checked based on the 5′ conserved motif GACUCUCG, and the corresponding length distribution of isoforms were analyzed. The genomic locations of the 5′5.8S rRF sequences were further analyzed using the annotated plant genomes from the Phytozome database (http://phytozome.jgi.doe.gov/pz/portal.html). The occurrence of the 5′5.8SrRF was checked from the 153 small RNA datasets of Arabidopsis next gen sequence database (https://mpss.danforthcenter.org).

### *In silico* analysis of the non-canonical short hairpin precursors and experimental mapping of 5′5.8S_L_ rRNA

5.8S ribosomal RNA of black pepper was analyzed *in silico* for its potential to form non-canonical, miRNA-like hairpin precursors. The secondary structures were predicted using the RNA folding program in the UEA small RNA workbench. Because the exact 5′ end of the 5.8S rRNA could not be accurately determined, the selected sequence of 5.8S rRNA contained an additional seven nucleotides upstream of the 5′ motif. The 5′ end of the 5.8S rRNA in black pepper was mapped using modified 5′ RLM RACE experiments. In brief, the total RNA isolated from black pepper was ligated at the 5′ end with the Gene Racer^TM^ RNA oligo (Gene RACER kit, Invitrogen, Carlsbad, CA). The ligated RNA was purified using phenol: chloroform and precipitated. The reverse transcription was performed using the rRNA specific reverse primer (5′CAACTTGCGTTCAAAGACTCGATGGT3′), and PCR amplification was carried out using the Gene Racer 5′ primer and 5.8S rRNA-specific reverse primer. The amplicons of ~150 bp in length were eluted from the gel and cloned into the PCR4 TOPO cloning vector (Invitrogen) and sequenced. The 5.8S rRNAs from different plant lineages were multiple aligned and a consensus structure was predicted. The alternate secondary structures were predicted for the 5.8S_L_ rRNA (including 7 upstream nt) from all the plant species studied. The cleavage patterns were assessed upstream of the GACTCT consensus and the length distribution at each upstream nucleotide position were analyzed. The sequence logo of abundant 5′5.8S rRFs (>100 RPM) were generated using RNALogo (*rnalogo.mbc.nctu.edu.tw/*)^52^.

### Prediction and validation of molecular functions of 5′5.8S rRFs

To study the role of rRFs in the RNAi pathway, high-throughput sequencing datasets of small RNAs were co-immunoprecipitated with AGO proteins from *Arabidopsis thaliana* and *Oryza sativa*. The details of the sRNA sequence dataset used were shown in Supplementary Table 4. The datasets consisted of immunoprecipitated small RNAs from AGO1 (GSM707682- GSM707685)^38^, AGO4 (GSM707686-GSM707689)^38^, AGO2 (GSM304282 and GSM304283)^23^, AGO7 (GSM304284-GSM304285)^23^, AGO6 (GSM415789 andGSM415790)^22^ and AGO9 (GSM415791 and GSM415792)^22^ from *A. thaliana*, and with different AGO1 homologs (GSM455962- GSM455965) from *O. sativa*^40^. The studies of Wu *et al*., 2009 generated AGO1 knock down rice plants with an RNAi approach that consisted of an inverted repeat (IR) construct (AGO1IR) for targeting the four rice AGO1 homologs such as AGO1a, b, c, and d. The relative position of srRNAs in the selected libraries was determined by mapping the reads to ribosomal RNA using PatMaN in SiLoCo and VisSR Tool of UEA small RNA workbench (srna-workbench.cmp.uea.ac.uk)^53^. The srRNAs were analyzed for their length distribution pattern and cleavage around the unique signature motif. The read counts of different length variants were normalized and represented in RPM (Reads Per Million). The higher abundance and biogenesis from the short hairpin structure and the association with AGO indicated the potential functional role of 5′5.8S rRFs, which is similar to microRNAs in the plant system. The dependency of the rRF candidates to Dicer-like (DCL) proteins were checked from GSE44622 that corresponds to sRNA datasets of partial loss of function *dcl-1* and *dcl-234* triple mutants^42^. The most prevalent 5.8S srRNA isoforms were selected and their targets were predicted from the black pepper mRNA transcriptome using psRNATarget (http://plantgrn.noble.org/psRNATarget/)^57^. The targets were also predicted for the 5′5.8S rRFs from other plant species from the psRNATarget. To further validate the predicted cleavage on the sRNA–target pairs, the degradome library was searched for the cleavage product using the dRNA mapper tool from SoMART web server (http://somart.ist.berkeley.edu). The cleavage from the predicted target of *A. thaliana* was analysed from the PARE (Parallel Analysis of RNA Ends) sequencing data from Col-0 inflorescence (GSM280226) and xrn4 mutant inflorescence (GSM280226).

### Validation of rRF mediated cleavage of the target mRNAs of the Ribosomal Protein RPS13

The 5′5.8S rRF mediated cleavage of the target mRNAs of RPS13 was experimentally mapped using modified 5′RLM RACE experiments^51^. Total RNA was isolated from black pepper using mirVana miRNA isolation kit (Ambion), according to the manufacturer’s instructions. The GeneRacer^TM^ RNA oligo adapter (Invitrogen) (5′CGACUGGAGCACGAGGACACUGACAUGGACUGAAGGAGUAGAAA3′) was ligated to the 5′ end of the cleaved mRNAs, without an alkaline phosphatase treatment. The reverse transcription of the ligated RNA was carried out at 50 °C for 60 min using GeneRacer^TM^ oligo dT primer and SuperScript™ III Reverse Transcriptase (Gene racer kit, Invitrogen). Followed by the inactivation of RT reaction at 70 °C for 15 min, the PCR amplification of the cDNA ends were performed at a thermal profile: 94 °C/2 min (1 cycle); 94 °C/30 s, 72 °C/1 min (5 cycles); 94 °C/30 s, 70 °C/1 min (5 cycles); 94 °C/30 s, 65 °C/30 s,72 °C/1 min (25 cycles); 72 °C/10 min (1cycle), using a forward primer specific to 5′ adapter sequences (5′CGACTGGAGCACGAGGACACTGA3′) and a reverse primer specific to the RPS13 gene (5′TGGAAAGGCGACATCACAACGA3′). Amplicons of the required size were extracted from 1.5% gel using S.N.A.P.™ Columns (Invitrogen) and cloned into the pCR4-TOPO cloning vector (Invitrogen). After transformation into competent *E. coli* strain DH5-Alpha, the plasmid DNA was isolated from the positive clones and sequenced with BigDye Terminator v3.1 Cycle Sequencing Kit (Applied Biosystems), using M13 forward primer (5′GTAAAACGACGGCCAG-3′) and M13 reverse primer (5′CAGGAAACAGCTATGAC3′) in ABI PRISM^®^ 3700 DNA Analyzer (ABI). The expression of the RPS13 mRNAs were further analyzed from the stress responsive mRNA transcriptome data (NCBI Sequence Read Archive under accession no. SRX853366 and SRX856639) (unpublished data) using FPKM method (Fragments Per kb per Million fragments)^58^.

## Additional Information

**How to cite this article:** Asha, S. and Soniya, E. V. The sRNAome mining revealed existence of unique signature small RNAs derived from 5.8SrRNA from *Piper nigrum* and other plant lineages. *Sci. Rep.* **7**, 41052; doi: 10.1038/srep41052 (2017).

**Publisher's note:** Springer Nature remains neutral with regard to jurisdictional claims in published maps and institutional affiliations.

## Supplementary Material

Supplementary Information

## Figures and Tables

**Figure 1:**
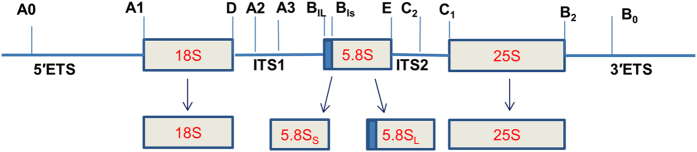
The cleavage points during the processing of 5.8S rRNA from pre-ribosomal RNA.

**Table 1:** Summary of ribosomal RNA derived small RNAs in the *P. nigrum* sRNA libraries of black pepper.

Library	Pn_CL	Pn_IL	Pn_IR
Total reads	18,629,003	12,621,368	12,948,539
rRNA mapped reads (srRNAs)	750,269	1,288,184	1,401,971
Total Unique srRNAs	50,926	60,277	122,031
Unique srRNAs (<10reads)	7,788	9,557	13,149

**Table 2:** The list of abundant srRNAs identified from *P. nigrum* sRNA libraries.

Sequence	Length	Read count in sRNA library		
		Pn_CL	Pn_IL	Pn_IR
CAGUACGACUCUCGGCAACGGA	22	13752	38322	25775
CAGUACGACUCUCGGCAACGGAU	23	13380	33983	23518
ACGACUCUCGGCAACGGAUAUCU	23	22983	30890	21796
GUACGACUCUCGGCAACGGAUAU	23	10125	30640	27894
CAGUACGACUCUCGGCAACGGAUA	24	7075	29330	22808
UACGACUCUCGGCAACGGAUAUCU	24	21900	27486	19404
CAGUACGACUCUCGGCAACGGAUAU	25	4069	24862	19808
ACGACUCUCGGCAACGGAUAU	21	11698	24572	30275
UACGACUCUCGGCAACGGAUAU	22	14729	18815	14578
GACUCUCGGCAACGGAUAUCU	21	7915	18098	11482
CAGUACGACUCUCGGCAACGG	21	14807	16489	10380
GUACGACUCUCGGCAACGGAUA	22	6009	15753	15029
ACGACUCUCGGCAACGGAUAUC	22	6496	13332	10836
GUACGACUCUCGGCAACGGAUAUCU	25	7360	10919	12142
GACUCUCGGCAACGGAUAUCUCGGCU	26	1866	10466	8135
GUACGACUCUCGGCAACGGAU	21	6218	10348	9506
ACGACUCUCGGCAACGGAUAUCUC	24	5945	9207	6776
AGUACGACUCUCGGCAACGGAUAU	24	2110	8104	5672
GUACGACUCUCGGCAACGGAUAUCUC	26	1889	8091	9523
UACGACUCUCGGCAACGGAUA	21	6991	7961	9531

**Table 3:** The relative position of major cleavage of 5.8S srRNAs from different plant species.

Plant group	Scientific Name	Major Cleavage site	Relative Position of major cleavage from 5′ consensus
Dicots	*Vitis vinifera*	C↓ACAAACG	−6
	*Carica papaya*	C↓AAAACG	−6
	*Citrus sinensis*	C↓AAAACG	−6
	*Gossipium arboreum*	U↓AGAACG	−6
	*Medicago truncatula*	U↓AGAAUG	−5
	*Arachis hypogea*	A↓AGAACG	−5
Dicots	*Glycine max*	U↓ACAAUG	−5
	*Cucurbita maxima*	C↓ACAACG	−5
	*Populus trichocarpa*	CUA↓AACG	−3
	*Arabidopsis thaliana*	UAA↓AACG	−3
	*Silene latifolia*	AUA↓AACG	−3
	*Mimulus guttatus*	C↓CAAAACG	−6
	*Solanum lycopersicum*	C↓ACAAACG	−6
	*Solanum tuberosum*	C↓ACAAACG	−6
	*Capsicum annuum*	A↓AAAACG	−5
	*Nicotiana tabaccum*	C↓ACAAACG	−6
Monocots	*Oryza sativa*	C↓CACACG	−5
	*Triticum aestivum*	C↓CACACG	−5
	*Hordeum vulgare*	C↓CACACG	−5
	*Setaria italica*	C↓CACACG	−5
	*Zea mays*	U↓CGACACG	−5
	*Sorghum bicolor*	C↓CACACG	−5
	*Panicum virgatum*	C↓AACACG	−5
	*Miscanthus giganteus*	C↓CACAUG	−5
	*Musa acuminata*	U↓UGGAUG	−5
Magnoliids	*Piper nigrum*	UCAG↓UACG	−3
	*Aristolochia fimbriata*	U↓AACACG	−5
	*Persea americana*	UUA↓GACG	−3
Other basal angiosperms	*Amborella sp*	↓AAACACG	−6
	*Nuphar advena*	CAAA↓ACG	−3
Gymnosperms	*Cycas rumphii*	A↓UGCACG	−5
	*Ginko biloba*	C↓CUCACG	−5
	*Picea abies*	G↓GAAAUG	−5
Pteridophyte	*Marsilea quadrifolia*	AAAA↓ACG	−3
Chlorophyta	*Chara coralina*	A↓GAACUG	−5

**Table 4:**
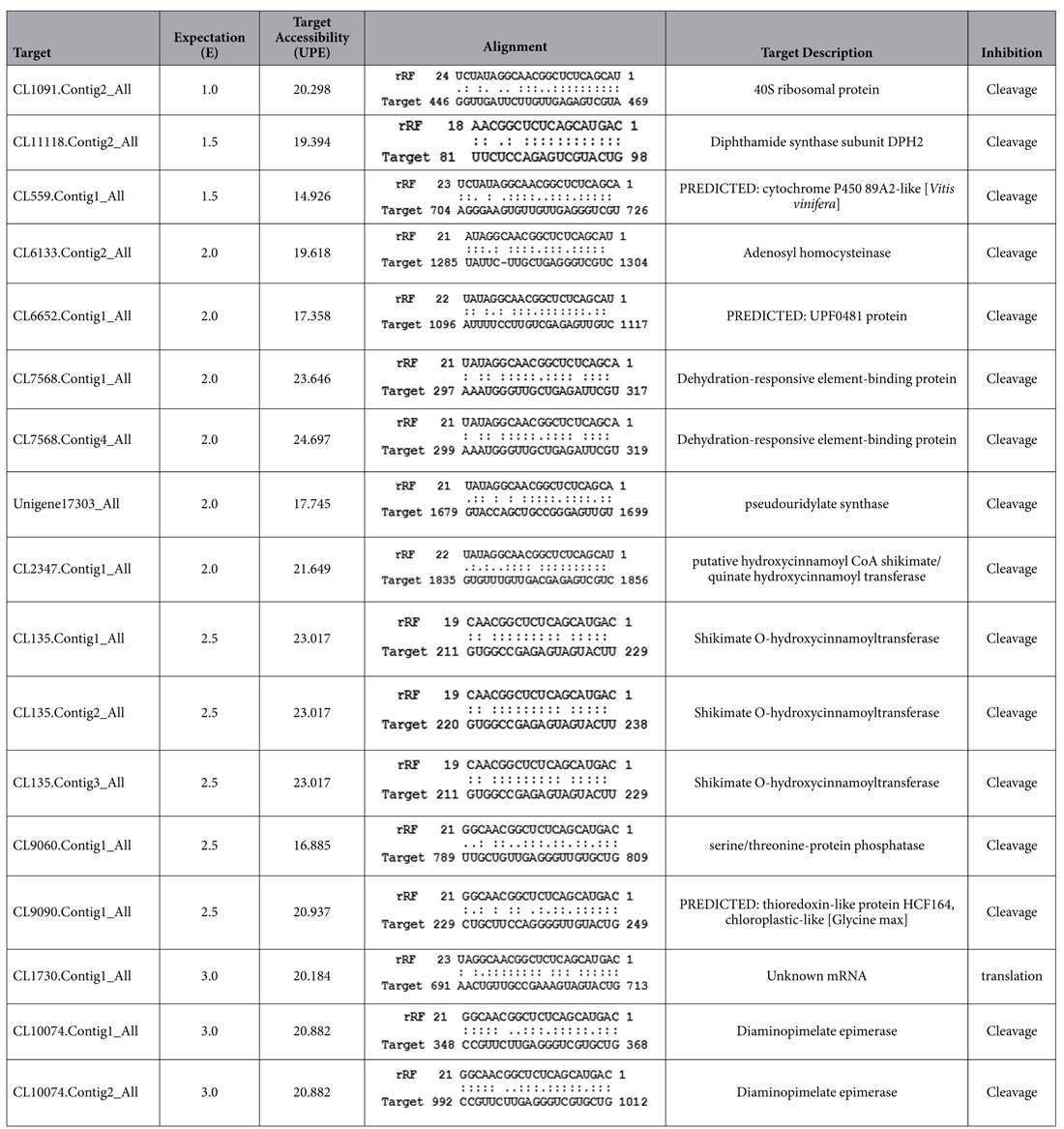
Targets predicted for 5′5.8S srRNAs from the black pepper transcriptome.
