# Supplementary material for: The sRNAome mining revealed existence of unique signature small RNAs derived from 5.8SrRNA from Piper nigrum and other plant lineages
Source: Sci Rep. 2017 Feb 1;7:41052. doi: 10.1038/srep41052 (PMC5286533; doi:10.1038/srep41052)
Supplement: Supplementary Information [file srep41052-s1.doc]

**The sRNAome mining revealed existence of unique signature small RNAs derived from 5.8SrRNA from *Piper nigrum* and other plant lineages.**

**Srinivasan Asha and E. V. Soniya**

**Supplementary Table 1. The predicted targets of the 5′5.8SrRFs from other plant species**

| **Plant species** | **Target database** | **Target Acc.** | **[Expectation (E)](http://plantgrn.noble.org/psRNATarget/?dowhat=Help#maxexpectation)** | **[Target Accessibility (UPE)](http://plantgrn.noble.org/psRNATarget/?dowhat=Help#upe)** | **Alignment** | **Target Description** | **Inhibition** |
| --- | --- | --- | --- | --- | --- | --- | --- |
| ***Arabidopsis thaliana*** | TAIR, version 10 | AT5G28056.1 | 3.5 | 18.702 | rRF 24 UCUAUAGGCAACGGCUCUCAGCAA 1  : .:. ..:::::.:::.::::::  Target 3483 AUGUGAUUGUUGCUGAGGGUCGUU3506 | transposable element gene | Cleavage |
|  |  | AT4G28850.1 | 3.5 | 20.252 | rRF 20 UAGGCAACGGCUCUCAGCAA 1  ::..:: ::::::.:::::  Target 671 AUUUGUGGCCGAGGGUCGUA 690 | xyloglucan endotransglucosylase/  hydrolase | Cleavage |
|  |  | [AT1G23310.2](http://plantgrn.noble.org/psRNATarget/getseq.do?sessionid=1420699874938401&source=target&seqID=AT1G23310.2) | 3.5 | 22.124 | rRF 20 UAGGCAACGGCUCUCAGCAA 1  :::::::::. ::. :::::  Target 933 AUCCGUUGCUCAGGCUCGUU 952 | glutamate:glyoxylate aminotransferase | Translation |
|  |  | AT1G23310.1 | 3.5 | 22.124 | rRF 20 UAGGCAACGGCUCUCAGCAA 1  :::::::::. ::. :::::  Target 933 AUCCGUUGCUCAGGCUCGUU 952 | glutamate:glyoxylate aminotransferase | Translation |
|  |  | AT4G08455.1 | 3.5 | 19.913 | rRF 24 UCUAUAGGCAACGGCUCUCAGCAA 1  : : ::::::: . : ::::::::  Target 265 AUAAAUCCGUUCUGGUGAGUCGUU288 | BTB/POZ domain-containing protein | Translation |
|  |  | AT3G18150.1 | 4.0 | 21.73 | rRF 21 AUAGGCAACGGCUCUCAGCAA 1  :::::::::::::: ::::  Target 926 UAUCCGUUGCCGAGCUUCGUG 946 | RNI-like superfamily protein | Cleavage |
|  |  | AT4G37260.1 | 4.0 | 20.057 | rRF 24 UCUA-UAGGCAACGGCUCUCAGCAA 1  :::: :::.::::: :::. :.:::  Target 172 AGAUGAUCUGUUGCAGAGGCUUGUU196 | myb domain protein 73 | Translation |
|  |  | AT2G06885.1 | 4.0 | 20.683 | rRF 20 UAGGCAACGGCUCUCAGCAA 1  ::.::::::.:::: ::: :  Target 1568 AUUCGUUGCUGAGAAUCGAU 1587 | transposable element gene | Cleavage |
|  |  | [AT5G20060.1](http://plantgrn.noble.org/psRNATarget/getseq.do?sessionid=1420699874938401&source=target&seqID=AT5G20060.1) | 4.0 | 12.274 | rRF 21 AUAGGCAACGGCUCUCAGCAA 1  :: :: ::::::::.::: ::  Target 37 UAACCCUUGCCGAGGGUCAUU 57 | alpha/beta-Hydrolases superfamily protein | Cleavage |
|  |  | AT1G30935.1 | 4.0 | 21.765 | rRF 22 UAUAGGCAACGGCUCUCAGCAA 1  : ::: :::::: .: ::::::  Target 58 AAAUCAGUUGCCAGGUGUCGUU 79 | pseudogene, F-box protein family, contains F-box domain | Translation |
|  |  | AT5G15810.1 | 4.0 | 23.383 | rRF 20 UAGGCAACGGCUCUCAGCAA 1  ::: :::::::: .:: :::  Target 936 AUCAGUUGCCGAUGGUGGUU 955 | N2,N2-dimethylguanosine tRNA methyltransferase | Cleavage |
|  |  | AT4G34310.2 | 4.0 | 24.371 | rRF 20 UAGGCAACGGCUCUCAGCAA 1  ::.::::: :::::: :::  Target 371 AUUCGUUGAGGAGAGUGGUU 390 | alpha/beta-Hydrolases superfamily protein | Translation |
|  |  | [AT4G34310.1](http://plantgrn.noble.org/psRNATarget/getseq.do?sessionid=1420699874938401&source=target&seqID=AT4G34310.1) | 4.0 | 24.371 | rRF 20 UAGGCAACGGCUCUCAGCAA 1  ::.::::: :::::: :::  Target 371 AUUCGUUGAGGAGAGUGGUU 390 | alpha/beta-Hydrolases superfamily protein | Translation |
|  |  | [ATMG01360.1](http://plantgrn.noble.org/psRNATarget/getseq.do?sessionid=1420699874938401&source=target&seqID=ATMG01360.1) | 4.5 | 20.023 | rRF 24 UCUAUAGGCAACGGCUCUCAGCAA 1  : :::::::: :..:.:: :.:::  Target 1382 AUAUAUCCGUAGUUGGGAUUUGUU1405 | cytochrome oxidase | Cleavage |
|  |  | [AT3G30213.1](http://plantgrn.noble.org/psRNATarget/getseq.do?sessionid=1420699874938401&source=target&seqID=AT3G30213.1) | 4.5 | 15.378 | rRF 23 CUAUAGGCAACGGCUCUCAGCAA 1  :: ::.:::::..:::: ::: :  Target 2187 GAAAUUCGUUGUUGAGAAUCGAU 2209 | transposable element gene | Cleavage |
|  |  | [AT1G23180.1](http://plantgrn.noble.org/psRNATarget/getseq.do?sessionid=1420699874938401&source=target&seqID=AT1G23180.1) | 4.5 | 20.236 | rRF 20 UAGGCAACGGCUCUCAGCAA 1  :::::::::.:::::: :  Target 950 AUCCGUUGCUGAGAGUGGAG 969 | ARM repeat superfamily protein | Cleavage |
|  |  | [AT3G11370.1](http://plantgrn.noble.org/psRNATarget/getseq.do?sessionid=1420699874938401&source=target&seqID=AT3G11370.1) | 4.5 | 14.476 | rRF 24 UCUAUAGGCAACGGCUCUCAGCAA 1  ::: ::.::::::: ::.::.::  Target 1317 AGAAAUUCGUUGCC-AGGGUUGUC1339 | Cysteine/Histidine-rich C1 domain family protein | Translation |
|  |  | [AT3G22900.1](http://plantgrn.noble.org/psRNATarget/getseq.do?sessionid=1420699874938401&source=target&seqID=AT3G22900.1) | 4.5 | 18.967 | rRF 23 CUAUAGGCAACGGCUCUCAGCAA 1  :.:.::.::: :::: :::.:::  Target 40 GGUGUCUGUUUCCGA-AGUUGUU 61 | RNA polymerase Rpb7-like, N-terminal domain | Cleavage |
|  |  | [AT3G01180.1](http://plantgrn.noble.org/psRNATarget/getseq.do?sessionid=1420699874938401&source=target&seqID=AT3G01180.1) | 4.5 | 15.0 | rRF 23 CUAUAGGCAACGGCUCUCAGCAA 1  :. ::: ::::::::::: . ::  Target 170 GGCAUCAGUUGCCGAGAGCUCUU 192 | starch synthase 2 | Cleavage |
|  |  | [AT2G32780.1](http://plantgrn.noble.org/psRNATarget/getseq.do?sessionid=1420699874938401&source=target&seqID=AT2G32780.1) | 4.5 | 19.068 | rRF 24 UCUAUAGGCAACGGCUCUCAGCAA 1  : : ::..:::::.:. :::.::  Target 469 AAAGAUUUGUUGCUGGAAGUUGUG 492 | ubiquitin-specific protease 1 | Cleavage |
|  |  | [AT5G09350.1](http://plantgrn.noble.org/psRNATarget/getseq.do?sessionid=1420699874938401&source=target&seqID=AT5G09350.1) | 4.5 | 18.004 | rRF 21 AUAGGCAACGGCUCUCAGCAA 1  :::::: : .:::::::. ::  Target 763 UAUCCGGUAUCGAGAGUUAUU 783 | phosphatidylinositol 4-OH kinase beta2 | Cleavage |
|  |  | AT2G28980.1 | 4.5 | 19.407 | rRF 21 AUAGGCAACGGCUCUCAGCAA 1  :: :.::::. ::::::: ::  Target 2113 UACCUGUUGUAGAGAGUCAUU 2133 | transposable element gene | Translation |
| ***Arabidopsis thaliana*** | TAIR, version 10 | AT5G61540.1 | 4.5 | 18.849 | miRNA 23 CUAUAGGCAACGGCUCUCAGCAA 1  :. : :.::::..:: .::.:::  Target 227 GGAAGCUGUUGUUGAAGGUUGUU 249 | N-terminal nucleophile aminohydrolases | Cleavage |
|  |  | AT5G45775.1 | 5.0 | 20.104 | miRNA 24 UCUAUAGGCAACGGCUCUCAGCAA 1  : :: ::::::: .:::::: ::  Target 128 ACAUUUCCGUUGGUGAGAGUGGUG 151 | Ribosomal L5P family protein | Cleavage |
|  |  | AT5G45775.1 | 5.0 | 20.104 | miRNA 24 UCUAUAGGCAACGGCUCUCAGCAA 1  : :: ::::::: .:::::: ::  Target 128 ACAUUUCCGUUGGUGAGAGUGGUG 151 | Ribosomal L5P family protein | Cleavage |
|  |  | AT5G45775.1 | 5.0 | 20.104 | miRNA 22 UAUAGGCAACGGCUCUCAGCAA 1  :: ::::::: .:::::: ::  Target 130 AUUUCCGUUGGUGAGAGUGGUG 151 | Ribosomal L5P family protein | Cleavage |
|  |  | AT5G45775.1 | 5.0 | 20.104 | miRNA 22 UAUAGGCAACGGCUCUCAGCAA 1  :: ::::::: .:::::: ::  Target 130 AUUUCCGUUGGUGAGAGUGGUG 151 | Ribosomal L5P family protein | Cleavage |
|  |  | AT5G45775.1 | 5.0 | 20.104 | miRNA 21 AUAGGCAACGGCUCUCAGCAA 1  : ::::::: .:::::: ::  Target 131 UUUCCGUUGGUGAGAGUGGUG 151 | Ribosomal L5P family protein | Cleavage |
|  |  | AT5G45775.1 | 5.0 | 20.104 | miRNA 24 UCUAUAGGCAACGGCUCUCAGCAA 1  : :: ::::::: .:::::: ::  Target 128 ACAUUUCCGUUGGUGAGAGUGGUG 151 | Ribosomal L5P family protein | Cleavage |
| ***Oryza sativa*** | TIGR genome cDNA OSA1 Release 5  (OSA1R5) | [LOC_Os03g03260.1](http://plantgrn.noble.org/psRNATarget/getseq.do?sessionid=1420698383708067&source=target&seqID=LOC_Os03g03260.1) | 2.5 | 22.637 | rRF 22 UAGGCAACGGCUCUCAGCACAC 1  ::: :::::::.:.::.:::::  Target 2487 AUCGGUUGCCGGGGGUUGUGUG 2508 | cDNA|homeobox domain containing protein, expressed | Cleavage |
|  |  | [LOC_Os11g15040.2](http://plantgrn.noble.org/psRNATarget/getseq.do?sessionid=1420698383708067&source=target&seqID=LOC_Os11g15040.2) | 3.0 | 18.812 | rRF 24 UAUAGGCAACGGCUCUCAGCACAC 1  :: ..:::::.:::.:::::::  Target 828 AUUCUUGUUGCUGAGGGUCGUGUU851 | S-adenosyl-L-methionine:benzoic acid/salicylic acid carboxyl methyltransferase, putative, expressed | Cleavage |
|  | DFCI Gene index (OSGI), version 19 | [TC500889](http://plantgrn.noble.org/psRNATarget/getseq.do?sessionid=1420698033722015&source=target&seqID=TC500889) | 3.0 | 18.812 | rRF 24 UAUAGGCAACGGCUCUCAGCACAC 1  :: ..:::::.:::.:::::::  Target 851 AUUCUUGUUGCUGAGGGUCGUGUU 874 | Benzothiadiazole-induced S-adenosyl-L-methionine:salicylic acid carboxyl methyltransferase 1 | Cleavage |
|  |  | [TC495556](http://plantgrn.noble.org/psRNATarget/getseq.do?sessionid=1420698033722015&source=target&seqID=TC495556) | 3.0 | 23.648 | rRF 20 GGCAACGGCUCUCAGCACAC 1  ..:::::.:::.:::::::  Target 746 UUGUUGCUGAGGGUCGUGUU 765 | SAM dependent carboxyl methyltransferase; | Cleavage |
| ***Hordeum vulgare*** | DFCI Gene Index (HVGI), version 12, | TC242536 | 5.0 | 19.333 | rRF 23 AUAGGCAACGGCUCUCAGCACAC 1  :.:.: ::::.:::.::::: ::  Target 499 UGUUC-UUGCUGAGGGUCGUCUG 520 | Adenosylhomocysteinase | Cleavage |
|  |  | TC260399 | 5.0 | 19.93 | rRF 23 AUAGGCAACGGCUCUCAGCACAC 1  :.:.: ::::.:::.::::: ::  Target 1210 UGUUC-UUGCUGAGGGUCGUCUG1231 | Adenosylhomocysteinase | Cleavage |
|  |  | TC261912 | 4.5 | 15.874 | rRF 23 AUAGGCAACGGCUCUCAGCACAC 1  :::::::::.: :::::.:: ::  Target 351 UAUCCGUUGUC-AGAGUUGUUUG 372 | DNAJ heat shock N-terminal domain-containing protein-like | Cleavage |
| [***Sorghum***](http://plantgrn.noble.org/psRNATarget/getseq.do?sessionid=1420701604360281&source=srna&seqID=S1) ***bicolor*** | *Sorghum bicolor* unigene, DFCI Gene Index (SBGI), version 9 | TC126487 | 4.5 | 23.713 | rRF 23 AUAGGCAACGGCUCUCAGCACAC 1  :.::: ::::.:::.::::: ::  Target 1197 UGUCC-UUGCUGAGGGUCGUUUG 1218 | Adenosylhomocysteinase | Cleavage |
|  |  | CD208148 | 4.5 | 24.455 | rRF 20 GGCAACGGCUCUCAGCACAC 1  :::::::: ::.::: :::  Target 152 CCGUUGCCCAGGGUCCCGUG 171 | Heat shock 70 kDa protein | Cleavage |
| [***Triticum***](http://plantgrn.noble.org/psRNATarget/getseq.do?sessionid=1420700976324167&source=srna&seqID=S1) ***aestivum*** | *Triticum aestivum* (wheat), unigene, DFCI Gene Index (TAGI), version 12  Triticum aestivum (wheat), unigene, DFCI Gene Index (TAGI), version 12,released on 2010_04_18 | [TC393868](http://plantgrn.noble.org/psRNATarget/getseq.do?sessionid=1420700976324167&source=target&seqID=TC393868) | 3.5 | 16.441 | rRF 21 AGGCAACGGCUCUCAGCACAC 1  ::.:::::: . .::::::::  Target 496 UCUGUUGCCUGUGGUCGUGUG 516 | similar to UniRef100_Q40151 Cluster: Hsc70 | Translation |
|  |  | TC437024 | 4.0 | 22.51 | rRF 23 AUAGGCAACGGCUCUCAGCACAC 1  :.:.: ::::.:::.::::: ::  Target 514 UGUUCCUUGCUGAGGGUCGUCUG 536 | Adenosylhomocysteinase | Cleavage |
|  |  | [TC425089](http://plantgrn.noble.org/psRNATarget/getseq.do?sessionid=1420700976324167&source=target&seqID=TC425089) | 4.0 | 24.718 | rRF 23 AUAGGCAACGGCUCUCAGCACAC 1  : :..::: ..:.::::.:::::  Target 693 UUUUUGUUCUUGGGAGUUGUGUG 715 | Alcohol dehydrogenase ADH2H | Cleavage |
|  |  | [TC380697](http://plantgrn.noble.org/psRNATarget/getseq.do?sessionid=1420700976324167&source=target&seqID=TC380697) | 4.0 | 21.024 | rRF 22 UAGGCAACGGCUCUCAGCACAC 1  .:.::: ::::::::: ::::  Target 1002 GUUCGUAACCGAGAGUCAUGUG 1023 | Ribonucleoside-diphosphate reductase | Cleavage |
|  |  | [CA661262](http://plantgrn.noble.org/psRNATarget/getseq.do?sessionid=1420700976324167&source=target&seqID=CA661262) | 4.0 | 24.841 | rRF 23 AUAGGCAACGGCUCUCAGCACAC 1  :. :: ::::::::::: : :::  Target 10 UGCCCAUUGCCGAGAGUGGAGUG 32 | Glyoxalase family-like protein | Cleavage |
|  |  | [TC426440](http://plantgrn.noble.org/psRNATarget/getseq.do?sessionid=1420700976324167&source=target&seqID=TC426440) | 4.5 | 23.151 | rRF 22 UAGGCAACGGCUCUCAGCACAC 1  .::::: : :::::: :::::  Target 204 GUCCGUCGAGGAGAGUGGUGUG 225 | Cysteine proteinase precursor | Cleavage |
|  |  | DR734708 | 4.0 | 24.915 | rRF 23 AUAGGCAACGGCUCUCAGCACAC 1  : :.:: :: ::.:: :::::::  Target 918 UCUUCGCUGACGGGACUCGUGUG 940 | Heat shock factor-binding protein 1 | Cleavage |
|  |  | GH732621 | 4.5 | 20.272 | rRF 22 UAGGCAACGGCUCUCAGCACAC 1  .:.::: ::::.:: :: ::::  Target 179 GUUCGUCGCCGGGACUCCUGUG 200 | PBS lyase HEAT domain protein repeat-containing protein | Cleavage |
|  |  | [TC389443](http://plantgrn.noble.org/psRNATarget/getseq.do?sessionid=1420700976324167&source=target&seqID=TC389443) | 5.0 | 18.87 | rRF 20 GGCAACGGCUCUCAGCACAC 1  :::::::: :::::::  Target 1374 CAUAUGCCGAGAAUCGUGUG 1393 | Acyl-CoA oxidase | Cleavage |
| [***Zea***](http://plantgrn.noble.org/psRNATarget/getseq.do?sessionid=1420706915450376&source=srna&seqID=S1) ***mays*** | *Zea mays* unigene, DFCI Gene Index (ZMGI), version 19 | FL021680 | 5.0 | 24.341 | rRF 21 GGCAACGGCUCUCAGCACAGC 1  :::::::: ::.::: ::.:  Target 128 CCGUUGCCCAGGGUCCCGUUG 148 | Heat shock 70 kDa protein, mitochondrial precursor | Cleavage |
|  |  | [TC491782](http://plantgrn.noble.org/psRNATarget/getseq.do?sessionid=1420706915450376&source=target&seqID=TC491782) | 0.0 | 19.023 | rRF 23 UAGGCAACGGCUCUCAGCACAGC 1  :::::::::::::::::::::::  Target 539 AUCCGUUGCCGAGAGUCGUGUCG 561 | BHLH transcription factor | Cleavage |
|  |  | [CD977855](http://plantgrn.noble.org/psRNATarget/getseq.do?sessionid=1420706915450376&source=target&seqID=CD977855) | 3.0 | 24.732 | rRF 20 GCAACGGCUCUCAGCACAGC 1  ::: ::.:: .:::::::::  Target 89 CGUCGCUGAUGGUCGUGUCG 108 | Zinc finger CCCH domain-containing protein ZFN-like 2 | Translation |
|  |  | [FL120932](http://plantgrn.noble.org/psRNATarget/getseq.do?sessionid=1420706915450376&source=target&seqID=FL120932) | 3.5 | 17.942 | rRF 20 GCAACGGCUCUCAGCACAGC 1  .:::: .:::.::::::::  Target 188 UGUUGNUGAGGGUCGUGUCA 207 | Triosephosphate isomerase, cytosolic | Cleavage |
| ***Citrus sinensis*** | *Citrus sinensis* (Orange), unigene, DFCI Gene Index (CSGI), version 1 | [TC6277](http://plantgrn.noble.org/psRNATarget/getseq.do?sessionid=1424360528586178&source=target&seqID=TC6277) | 3.0 | 18.184 | rRF 22 UAGGCAACGGCUCUCAGCAAAA 1  :::.:::::::: : :.:::::  Target 722 AUCUGUUGCCGAAAUUUGUUUU 743 | 40S ribosomal protein S15 | Translation |
|  |  | [EY700955](http://plantgrn.noble.org/psRNATarget/getseq.do?sessionid=1424360528586178&source=target&seqID=EY700955) | 3.5 | 23.596 | rRF 22 UAGGCAACGGCUCUCAGCAAAA 1  .:::: ::::.:.::.:::::  Target 840 GUCCGCCGCCGGGGGUUGUUUU 861 | Radical SAM enzyme, Cfr family protein | Cleavage |
|  |  | [TC23504](http://plantgrn.noble.org/psRNATarget/getseq.do?sessionid=1424360528586178&source=target&seqID=TC23504) | 4.0 | 14.873 | rRF 21 AGGCAACGGCUCUCAGCAAAA 1  ::: :::..:: ::::::::  Target 635 UCCAUUGUUGAUAGUCGUUUC 655 | Dem protein | Translation |
|  |  | [EY670560](http://plantgrn.noble.org/psRNATarget/getseq.do?sessionid=1424360528586178&source=target&seqID=EY670560) | 4.0 | 20.552 | rRF 21 AGGCAACGGCUCUCAGCAAAA 1  :. :::: : ::.::::::::  Target 725 UUGGUUGACCAGGGUCGUUUU 745 | Alpha-glucan water dikinase, chloroplast precursor | Cleavage |
|  |  | [EY758138](http://plantgrn.noble.org/psRNATarget/getseq.do?sessionid=1424360528586178&source=target&seqID=EY758138) | 4.5 | 18.504 | rRF 23 AUAGGCAACGGCUCUCAGCAAAA 1  ::: :: ::..:::::::::: :  Target 583 UAUGCGGUGUUGAGAGUCGUUCU 605 | 40S ribosomal protein S19-like | Cleavage |
|  |  | [TC14950](http://plantgrn.noble.org/psRNATarget/getseq.do?sessionid=1424360528586178&source=target&seqID=TC14950) | 4.5 | 23.935 | rRF 23 AUAGGCAACGGCUCUCAGCAAAA 1  : ::: : ::::.: :::::::  Target 739 UUUCCAUCGCCGGGCGUCGUUUA 761 | Non-specific lipid-transfer protein | Translation |
|  |  | [EY716051](http://plantgrn.noble.org/psRNATarget/getseq.do?sessionid=1424360528586178&source=target&seqID=EY716051) | 4.5 | 18.068 | rRF 23 AUAGGCAACGGCUCUCAGCAAAA 1  :. :: ::::. .::::.::::  Target 819 UGACCAUUGCUUGGAGUUGUUUA 841 | O-methyltransferase | Cleavage |
|  |  | [EY749697](http://plantgrn.noble.org/psRNATarget/getseq.do?sessionid=1424360528586178&source=target&seqID=EY749697) | 4.5 | 16.505 | rRF 22 UAGGCAACGGCUCUCAGCAAAA 1  :::: : : :::.::::::::  Target 136 AUCCUCUCCGGAGGGUCGUUUU 157 | Glucose-6-phosphate isomerase | Cleavage |
|  |  | [TC17705](http://plantgrn.noble.org/psRNATarget/getseq.do?sessionid=1424360528586178&source=target&seqID=TC17705) | 4.5 | 16.505 | rRF 22 UAGGCAACGGCUCUCAGCAAAA 1  :::: : : :::.::::::::  Target 557 AUCCUCUCCGGAGGGUCGUUUU 578 | Glucose-6-phosphate isomerase | Cleavage |
|  |  | [EY724411](http://plantgrn.noble.org/psRNATarget/getseq.do?sessionid=1424360528586178&source=target&seqID=EY724411) | 4.5 | 20.234 | rRF 20 GGCAACGGCUCUCAGCAAAA 1  :::: : ::.::::::::  Target 236 UGGUUGACCAGGGUCGUUUU 255 | Alpha-glucan water dikinase, chloroplast precursor | Cleavage |
|  |  | [EY748745](http://plantgrn.noble.org/psRNATarget/getseq.do?sessionid=1424360528586178&source=target&seqID=EY748745) | 4.5 | 21.051 | rRF 20 GGCAACGGCUCUCAGCAAAA 1  :: :::: :.:.::.::::  Target 580 CCAUUGCAGGGGGUUGUUUC 599 | Peptidyl-prolyl cis-trans isomerase | Cleavage |
|  |  | [CK739457](http://plantgrn.noble.org/psRNATarget/getseq.do?sessionid=1424360528586178&source=target&seqID=CK739457) | 4.5 | 17.818 | rRF 20 GGCAACGGCUCUCAGCAAAA 1  :: ::::.:::: .:::::  Target 73 CCUUUGCUGAGAAGUGUUUU 92 | Mutator-like transposase | Cleavage |
|  |  | [EY737091](http://plantgrn.noble.org/psRNATarget/getseq.do?sessionid=1424360528586178&source=target&seqID=EY737091) | 5.0 | 21.788 | rRF 22 UAGGCAACGGCUCUCAGCAAAA 1  ::: ::: :.:::.::. ::::  Target 730 AUCAGUUACUGAGGGUUCUUUU 751 | Plastid phosphoenolpyruvate/phosphate translocator | Cleavage |
|  |  | [EY700828](http://plantgrn.noble.org/psRNATarget/getseq.do?sessionid=1424360528586178&source=target&seqID=EY700828) | 5.0 | 10.604 | rRF 22 UAGGCAACGGCUCUCAGCAAAA 1  .::::: :..:.:.::.::: :  Target 824 GUCCGUGGUUGGGGGUUGUUGU 845 | Probable transmembrane sensor | Cleavage |
|  |  | [EY701072](http://plantgrn.noble.org/psRNATarget/getseq.do?sessionid=1424360528586178&source=target&seqID=EY701072) | 5.0 | 13.093 | rRF 23 AUAGGCAACGGCUCUCAGCAAAA 1  :.: .: ::. :::.::.:::::  Target 755 UGUGUGGUGUGGAGGGUUGUUUU 777 | Cytochrome P450 92B1 | Cleavage |
|  |  | [TC11705](http://plantgrn.noble.org/psRNATarget/getseq.do?sessionid=1424360528586178&source=target&seqID=TC11705) | 5.0 | 12.891 | rRF 23 AUAGGCAACGGCUCUCAGCAAAA 1  ::: : ::::. : :::.:::::  Target 774 UAUACAUUGCUUAAAGUUGUUUU 796 | P-type H+-ATPase | Translation |
|  |  | [EY715151](http://plantgrn.noble.org/psRNATarget/getseq.do?sessionid=1424360528586178&source=target&seqID=EY715151) | 5.0 | 19.345 | rRF 22 UAGGCAACGGCUCUCAGCAAAA 1  :::: ::::: ::. :: ::::  Target 262 AUCCUUUGCCUAGGCUCAUUUU 283 | Molecular chaperone | Cleavage |
|  |  | [TC18637](http://plantgrn.noble.org/psRNATarget/getseq.do?sessionid=1424360528586178&source=target&seqID=TC18637) | 5.0 | 18.794 | rRF 21 AGGCAACGGCUCUCAGCAAAA 1  ::. :::.::: .:::::: :  Target 335 UCUUUUGUCGACGGUCGUUAU 355 | Cellulose synthase-like protein CslG | Translation |
|  |  | [CV885424](http://plantgrn.noble.org/psRNATarget/getseq.do?sessionid=1424360528586178&source=target&seqID=CV885424) | 5.0 | 17.168 | rRF 22 UAGGCAACGGCUCUCAGCAAAA 1  ::::: :: :. :::.:::::  Target 593 AUCCGGUGGAGGAAGUUGUUUU 614 | Casbene synthase, chloroplast precursor | Translation |
|  |  | [EY676466](http://plantgrn.noble.org/psRNATarget/getseq.do?sessionid=1424360528586178&source=target&seqID=EY676466) | 5.0 | 17.02 | rRF 20 GGCAACGGCUCUCAGCAAAA 1  :. :::.::::.::: :::  Target 588 CUUUUGUCGAGGGUCCUUUA 607 | Glyceraldehyde-3-phosphate dehydrogenase | Cleavage |
| ***Carica papaya*** | *Carica papaya* (Papaya), unigene, DFCI Gene Index (CAPAGI), version 1, | [TC5272](http://plantgrn.noble.org/psRNATarget/getseq.do?sessionid=1424360141011337&source=target&seqID=TC5272) | 4.5 | 16.764 | rRF 23 AUAGGCAACGGCUCUCAGCAAAA 1  : :.. : ::::.:.::.:::::  Target 3 UUUUUUUGGCCGGGGGUUGUUUU 25 | : Plasma membrane H+-ATPase; | Cleavage |
|  |  | [EX285424](http://plantgrn.noble.org/psRNATarget/getseq.do?sessionid=1424360141011337&source=target&seqID=EX285424) | 4.5 | 17.021 | rRF 22 UAGGCAACGGCUCUCAGCAAAA 1  :::: : :. :: :::::::::  Target 419 AUCCAUCGUGGAAAGUCGUUUU 440 | Dem protein | Translation |
|  |  | [TC9162](http://plantgrn.noble.org/psRNATarget/getseq.do?sessionid=1424360141011337&source=target&seqID=TC9162) | 4.5 | 19.715 | rRF 22 UAGGCAACGGCUCUCAGCAAAA 1  :::: : :. :: :::::::::  Target 1108 AUCCAUCGUGGAAAGUCGUUUU 1129 | Dem protein | Translation |
|  |  | [TC6733](http://plantgrn.noble.org/psRNATarget/getseq.do?sessionid=1424360141011337&source=target&seqID=TC6733) | 5.0 | 17.026 | rRF 21 AGGCAACGGCUCUCAGCAAAA 1  :.:::::.::.::::. :: :  Target 365 UUCGUUGUCGGGAGUUCUUCU 385 | NADP-dependent malic enzyme 1; | Cleavage |
|  |  | [TC723](http://plantgrn.noble.org/psRNATarget/getseq.do?sessionid=1424360141011337&source=target&seqID=TC723) | 5.0 | 16.891 | rRF 21 AGGCAACGGCUCUCAGCAAAA 1  :.:::::.::.::::. :: :  Target 977 UUCGUUGUCGGGAGUUCUUCU 997 | NADP-dependent malic enzyme 3 | Cleavage |
|  |  | [EX285849](http://plantgrn.noble.org/psRNATarget/getseq.do?sessionid=1424360141011337&source=target&seqID=EX285849) | 5.0 | 16.448 | rRF 22 UAGGCAACGGCUCUCAGCAAAA 1  :::.: : :: :::::.::::  Target 259 AUCUGCUACCCAGAGUUGUUUC 280 | 3-deoxy-manno-octulosonate cytidylyltransferase; | Cleavage |
|  |  | [EX267395](http://plantgrn.noble.org/psRNATarget/getseq.do?sessionid=1424360141011337&source=target&seqID=EX267395) | 5.0 | 14.067 | rRF 21 AGGCAACGGCUCUCAGCAAAA 1  :.:: :::: :: ::.::::  Target 930 UUCGAUGCCAAGUGUUGUUUC 950 | : HEAT repeat-containing protein | Translation |
| [***Gossipium***](http://plantgrn.noble.org/psRNATarget/getseq.do?sessionid=1424359321456603&source=srna&seqID=S1) ***sp*** | *Gossypium* (cotton), unigene, DFCI Gene Index (CGI), version 11 | [CO078353](http://plantgrn.noble.org/psRNATarget/getseq.do?sessionid=1424359321456603&source=target&seqID=CO078353) | 4.5 | 16.032 | rRF 20 GGCAACGGCUCUCAGCAAGA 1  : :::::.:.::.:::::  Target 207 AAGGUGCCGGGGGUUGUUCU 226 | Histone deacetylase | Cleavage |
|  |  | [TC3985](http://plantgrn.noble.org/psRNATarget/getseq.do?sessionid=1424359321456603&source=target&seqID=TC3985) | 4.5 | 16.639 | rRF 22 UAGGCAACGGCUCUCAGCAAGA 1  : :.::::: :::::: ::::  Target 1204 AACUGUUGCAGAGAGUAAUUCU 1225 | Ethanolamine-phosphate cytidylyltransferase 1 | Cleavage |
|  |  | [CO085294](http://plantgrn.noble.org/psRNATarget/getseq.do?sessionid=1424359321456603&source=target&seqID=CO085294) | 4.5 | 13.097 | rRF 21 AGGCAACGGCUCUCAGCAAGA 1  ::: ::::: :::.:::::  Target 21 UCCAUUGCCUUUAGUUGUUCU 41 | Granule bound starch synthase II precursor | Translation |
|  |  | [TC9109](http://plantgrn.noble.org/psRNATarget/getseq.do?sessionid=1424359321456603&source=target&seqID=TC9109) | 4.5 | 24.323 | rRF 20 GGCAACGGCUCUCAGCAAGA 1  :::: :.:: ::::: ::.:  Target 1224 CCGUAGUCGCGAGUCUUUUU 1243 | mitochondrion rrn26 gene for rRNA large subunit (26S), partial (17%) | Translation |
|  |  | [CO106097](http://plantgrn.noble.org/psRNATarget/getseq.do?sessionid=1424359321456603&source=target&seqID=CO106097) | 5.0 | 19.13 | rRF 23 AUAGGCAACGGCUCUCAGCAAGA 1  :::.: ::::.:::: :.:::.  Target 767 UAUUCAUUGCUGAGAAUUGUUUG 789 | Phloem calmodulin-like-domain protein kinase PCPK1 | Cleavage |
|  |  | [TC5941](http://plantgrn.noble.org/psRNATarget/getseq.do?sessionid=1424359321456603&source=target&seqID=TC5941) | 5.0 | 17.506 | rRF 24 UAUAGGCAACGGCUCU-CAGCAAGA 1  .: ::::::::: ::: ::: ::.:  Target 32 GUUUCCGUUGCCCAGAAGUCCUUUU56 | Actin | Translation |
|  |  | [TC4405](http://plantgrn.noble.org/psRNATarget/getseq.do?sessionid=1424359321456603&source=target&seqID=TC4405) | 5.0 | 16.186 | rRF 22 UAGGCAACGGCUCUCAGCAAGA 1  .:. ::: :.::::::.: :::  Target 786 GUUGGUUUCUGAGAGUUGAUCU 807 | Stress-related protein | Cleavage |
|  |  | [CO092634](http://plantgrn.noble.org/psRNATarget/getseq.do?sessionid=1424359321456603&source=target&seqID=CO092634) | 5.0 | 13.225 | rRF 22 UAGGCAACGGCUCUCAGCAAGA 1  .:::: : .:::::: :::::  Target 525 GUCCGAGGAUGAGAGUGGUUCU 546 | Histone deacetylase HDT1 | Cleavage |
|  |  | [TC7210](http://plantgrn.noble.org/psRNATarget/getseq.do?sessionid=1424359321456603&source=target&seqID=TC7210) | 5.0 | 21.659 | rRF 24 UAUAGGCAACGGCUCUCAGCAAGA 1  .:. .:: :::.:: .::: ::::  Target 963 GUGAUCGGUGCUGAUGGUCUUUCU 986 | Beta-ketoacyl-ACP synthase III | Translation |
|  |  | [TC570](http://plantgrn.noble.org/psRNATarget/getseq.do?sessionid=1424359321456603&source=target&seqID=TC570) | 5.0 | 13.225 | rRF 22 UAGGCAACGGCUCUCAGCAAGA 1  .:::: : .:::::: :::::  Target 614 GUCCGAGGAUGAGAGUGGUUCU 635 | Histone deacetylase HDT1 | Cleavage |
|  |  | [CO120447](http://plantgrn.noble.org/psRNATarget/getseq.do?sessionid=1424359321456603&source=target&seqID=CO120447) | 5.0 | 21.596 | rRF 22 UAGGCAACGGCUCUCAGCAAGA 1  : :.: ::..:::: :::::.  Target 253 AGCUGCUGUUGAGAAUCGUUUC 274 | F-box/LRR-repeat protein 5 | Cleavage |
|  |  | [CO096551](http://plantgrn.noble.org/psRNATarget/getseq.do?sessionid=1424359321456603&source=target&seqID=CO096551) | 5.0 | 16.464 | rRF 20 GGCAACGGCUCUCAGCAAGA 1  :: ::::. ::::: ::::  Target 105 CCUUUGCUAAGAGUGGUUCC 124 | calmodulin-binding protein | Cleavage |
|  |  | [CO081500](http://plantgrn.noble.org/psRNATarget/getseq.do?sessionid=1424359321456603&source=target&seqID=CO081500) | 5.0 | 18.552 | rRF 20 GGCAACGGCUCUCAGCAAGA 1  .: :::.:.:: :.:::::  Target 371 AUGAUGCUGGGACUUGUUCU 390 | Helix-loop-helix DNA-binding | Cleavage |
|  |  | [TC1883](http://plantgrn.noble.org/psRNATarget/getseq.do?sessionid=1424359321456603&source=target&seqID=TC1883) | 5.0 | 20.059 | rRF 24 UAUAGGCAACGGCUCUCAGCAAGA 1  : : ::: : :.:::.:: :::.:  Target 605 AAAACCGAUUCUGAGGGUGGUUUU628 | : Oxygen-evolving enhancer protein 2, chloroplast precursor | Cleavage |
| [***Populus***](http://plantgrn.noble.org/psRNATarget/getseq.do?sessionid=1424359594861260&source=srna&seqID=S1) ***trichocarpa*** | *Populus trichocarpa* (poplar), unigene, DFCI Gene Index (PPLGI), version 5 | [CN550256](http://plantgrn.noble.org/psRNATarget/getseq.do?sessionid=1424359594861260&source=target&seqID=CN550256) | 4.0 | 17.964 | rRF 21 AUAGGCAACGGCUCUCAGCAA 1  :::..:: :. :::.::::::  Target 434 UAUUUGUGGUGGAGGGUCGUU 454 | 60S ribosomal protein L15 | Translation |
|  |  | [TC159801](http://plantgrn.noble.org/psRNATarget/getseq.do?sessionid=1424359594861260&source=target&seqID=TC159801) | 4.5 | 18.648 | rRF 22 UAUAGGCAACGGCUCUCAGCAA 1  :: :..:::::.::: ::.:::  Target 518 AUUUUUGUUGCUGAGUGUUGUU 539 | Maturation-associated SRC1-like protein | Cleavage |
|  |  | [TC163008](http://plantgrn.noble.org/psRNATarget/getseq.do?sessionid=1424359594861260&source=target&seqID=TC163008) | 4.5 | 15.739 | rRF 20 UAGGCAACGGCUCUCAGCAA 1  ::.::::..::::::. ::  Target 949 UUCUGUUGUUGAGAGUUCUU 968 | Aminoimidazolecarboximide ribonucleotide transformylase/inosine monophosphate cyclohydrolase | Cleavage |
|  |  | [TC179100](http://plantgrn.noble.org/psRNATarget/getseq.do?sessionid=1424359594861260&source=target&seqID=TC179100) | 4.5 | 13.338 | rRF 21 AUAGGCAACGGCUCUCAGCAA 1  : ::.: :::::::: :.:::  Target 462 UUUCUGAUGCCGAGACUUGUU 482 | Peptidyl-prolyl cis-trans isomerase | Cleavage |
|  |  | [TC171800](http://plantgrn.noble.org/psRNATarget/getseq.do?sessionid=1424359594861260&source=target&seqID=TC171800) | 4.5 | 20.363 | rRF 22 UAUAGGCAACGGCUCUCAGCAA 1  ::::::::::: : :::: :::  Target 996 AUAUCCGUUGCAG-GAGUAGUU 1016 | Cytochrome P450 71D10 | Translation |
|  |  | [TC187511](http://plantgrn.noble.org/psRNATarget/getseq.do?sessionid=1424359594861260&source=target&seqID=TC187511) | 5.0 | 22.992 | rRF 21 AUAGGCAACGGCUCUCAGCAA 1  :::..:::::: :::: .::  Target 19 UAUUUGUUGCCUAGAGGUGUC 39 | Tropinone reductase | Translation |
|  |  | [TC155145](http://plantgrn.noble.org/psRNATarget/getseq.do?sessionid=1424359594861260&source=target&seqID=TC155145) | 5.0 | 17.95 | rRF 22 UAUAGGCAACGGCUCUCAGCAA 1  .::::. ::::::: :::::  Target 354 GUAUCUUGUGCCGAGUGUCGUG 375 | Protein yippee-like At4g27745 | Cleavage |
|  |  | [CF236016](http://plantgrn.noble.org/psRNATarget/getseq.do?sessionid=1424359594861260&source=target&seqID=CF236016) | 5.0 | 18.279 | rRF 22 UAUAGGCAACGGCUCUCAGCAA 1  : :::.:::::::::. :::  Target 355 AGAUCUGUUGCCGAGGAGCGUG 376 | Ribosomal protein L15 | Cleavage |
|  |  | [CF228163](http://plantgrn.noble.org/psRNATarget/getseq.do?sessionid=1424359594861260&source=target&seqID=CF228163) | 5.0 | 17.665 | rRF 22 UAUAGGCAACGGCUCUCAGCAA 1  : :::.:::::::::. :::  Target 354 AGAUCUGUUGCCGAGGAGCGUG 375 | Ribosomal protein L15 | Cleavage |
|  |  | [TC147449](http://plantgrn.noble.org/psRNATarget/getseq.do?sessionid=1424359594861260&source=target&seqID=TC147449) | 5.0 | 17.695 | rRF 22 UAUAGGCAACGGCUCUCAGCAA 1  : :::.:::::::::. :::  Target 352 AGAUCUGUUGCCGAGGAGCGUG 373 | Ribosomal protein L15 | Cleavage |
|  |  | [TC148029](http://plantgrn.noble.org/psRNATarget/getseq.do?sessionid=1424359594861260&source=target&seqID=TC148029) | 5.0 | 17.378 | rRF 22 UAUAGGCAACGGCUCUCAGCAA 1  ::.:.:: :. ::::::.:::  Target 544 AUGUUCGAGGUGGAGAGUUGUU 565 | CBL-interacting protein kinase 20 | Translation |
|  |  | [TC139171](http://plantgrn.noble.org/psRNATarget/getseq.do?sessionid=1424359594861260&source=target&seqID=TC139171) | 5.0 | 24.599 | rRF 22 UAUAGGCAACGGCUCUCAGCAA 1  ::.:.::: :. : ::::.:::  Target 331 AUGUUCGUGGUGGUGAGUUGUU 352 | UniRef100_A0MNK6 Cluster: CBL-interacting protein kinase 19 | Translation |
|  |  | [TC147274](http://plantgrn.noble.org/psRNATarget/getseq.do?sessionid=1424359594861260&source=target&seqID=TC147274) | 5.0 | 13.886 | rRF 20 UAGGCAACGGCUCUCAGCAA 1  ::::: ::::.::.:::  Target 128 GCACGUUGACGAGGGUUGUU 147 | Type II secretion system protein F domain protein | Cleavage |
|  |  | [CX176635](http://plantgrn.noble.org/psRNATarget/getseq.do?sessionid=1424359594861260&source=target&seqID=CX176635) | 5.0 | 14.174 | rRF 20 UAGGCAACGGCUCUCAGCAA 1  ::::: ::::.::.:::  Target 115 GCACGUUGACGAGGGUUGUU 134 | NADH-ubiquinone oxidoreductase chain 2 | Cleavage |
|  |  | [DN497868](http://plantgrn.noble.org/psRNATarget/getseq.do?sessionid=1424359594861260&source=target&seqID=DN497868) | 5.0 | 21.386 | rRF 22 UAUAGGCAACGGCUCUCAGCAA 1  .::::.:::: .: ::::::  Target 137 GUAUCUGUUGAUGUUAGUCGUC 158 | Ctr family transporter | Translation |
|  |  | [TC138648](http://plantgrn.noble.org/psRNATarget/getseq.do?sessionid=1424359594861260&source=target&seqID=TC138648) | 5.0 | 20.366 | rRF 21 AUAGGCAACGGCUCUCAGCAA 1  :::. : ::.::::.::.::  Target 752 UAUUGGCUGUCGAGGGUUGUG 772 | Serine/threonine-protein phosphatase 2A 65 kDa regulatory subunit A beta isoform | Cleavage |
|  |  | [DT477706](http://plantgrn.noble.org/psRNATarget/getseq.do?sessionid=1424359594861260&source=target&seqID=DT477706) | 5.0 | 12.294 | rRF 20 UAGGCAACGGCUCUCAGCAA 1  :. :::::.:::: :.:::  Target 778 UUUAGUUGCUGAGAUUUGUU 797 | Glucose-1-phosphate adenylyltransferase | Cleavage |
|  |  | [TC149483](http://plantgrn.noble.org/psRNATarget/getseq.do?sessionid=1424359594861260&source=target&seqID=TC149483) | 5.0 | 14.767 | rRF 22 UAUAGGCAACGGCUCUCAGCAA 1  ::. : ::::::::::.:::  Target 760 AUGCCAUCUGCCGAGAGUUGUU 781 | Pectin methylesterase | Cleavage |
|  |  | [TC146059](http://plantgrn.noble.org/psRNATarget/getseq.do?sessionid=1424359594861260&source=target&seqID=TC146059) | 5.0 | 20.626 | rRF 20 UAGGCAACGGCUCUCAGCAA 1  ::..: :::.:.::::.: :  Target 700 AUUUGCUGCUGGGAGUUGAU 719 | CUE domain containing protein, expressed | Cleavage |
|  |  | [TC186494](http://plantgrn.noble.org/psRNATarget/getseq.do?sessionid=1424359594861260&source=target&seqID=TC186494) | 5.0 | 24.764 | rRF 20 UAGGCAACGGCUCUCAGCAA 1  ::: :::::::::. :.: :  Target 67 AUCAGUUGCCGAGGAUUGAU 86 | Homeobox protein knotted-1-like 3 | Cleavage |
| ***Glycine max*** | *Glycine max* (soybean), unigene, DFCI Gene Index (GMGI) version 16 | [TC440456](http://plantgrn.noble.org/psRNATarget/getseq.do?sessionid=1424358326993263&source=target&seqID=TC440456) | 4.0 | 17.078 | rRF 24 UAUAGGCAACGGCUCUCAGUAACA 1  ::: :.:::::: ::::::::: :  Target 711 AUAGCUGUUGCC-AGAGUCAUUCU733 | 50S ribosomal protein L28, chloroplast precursor | Cleavage |
|  |  | [TC450859](http://plantgrn.noble.org/psRNATarget/getseq.do?sessionid=1424358326993263&source=target&seqID=TC450859) | 4.0 | 13.294 | rRF 20 GGCAACGGCUCUCAGUAACA 1  :.:::::.:: :::.::: :  Target 166 CUGUUGCUGAUAGUUAUUAU 185 | SNF1-related kinase | Translation |
|  |  | [BI470345](http://plantgrn.noble.org/psRNATarget/getseq.do?sessionid=1424358326993263&source=target&seqID=BI470345) | 4.0 | 17.352 | rRF 20 GGCAACGGCUCUCAGUAACA 1  :::::: ::: :::::::  Target 64 AAGUUGCCUAGAUUCAUUGU 83 | PPR-repeat containing protein | Cleavage |
|  |  | [TC470472](http://plantgrn.noble.org/psRNATarget/getseq.do?sessionid=1424358326993263&source=target&seqID=TC470472) | 4.0 | 23.166 | rRF 20 GGCAACGGCUCUCAGUAACA 1  : ::: ::::::::::::  Target 225 ACAUUGGCGAGAGUCAUUGG 244 | Citrate synthase, glyoxysomal precursor | Cleavage |
|  |  | [TC445978](http://plantgrn.noble.org/psRNATarget/getseq.do?sessionid=1424358326993263&source=target&seqID=TC445978) | 4.0 | 24.924 | rRF 21 AGGCAACGGCUCUCAGUAACA 1  :::::: :. .:: :::::::  Target 56 UCCGUUACUCGGAUUCAUUGU 76 | Starch synthase II-2 | Cleavage |
|  |  | [TC471270](http://plantgrn.noble.org/psRNATarget/getseq.do?sessionid=1424358326993263&source=target&seqID=TC471270) | 4.0 | 22.096 | rRF 20 GGCAACGGCUCUCAGUAACA 1  : ::: ::::::::::::  Target 674 ACAUUGGCGAGAGUCAUUGG 693 | Citrate synthase, glyoxysomal precursor | Cleavage |
|  |  | [TC422475](http://plantgrn.noble.org/psRNATarget/getseq.do?sessionid=1424358326993263&source=target&seqID=TC422475) | 4.5 | 16.478 | rRF 24 UAUAGGCAACGGCUCUCAGUAACA 1  :::::::: :..:.:: :..::::  Target 1721 AUAUCCGUAGUUGGGAUUUGUUGU1744 | Cytochrome c oxidase subunit 1 | Cleavage |
|  |  | [BE804093](http://plantgrn.noble.org/psRNATarget/getseq.do?sessionid=1424358326993263&source=target&seqID=BE804093) | 4.5 | 15.25 | rRF 24 UAUAGGCAACGGCUCUCAGUAACA 1  ::::. :::::.:: ::.:::::  Target 159 AUAUUAGUUGCUGAAUGUUAUUGU 182 | Potato DNA for copia-like transposable element; | Translation |
|  |  | [TC486115](http://plantgrn.noble.org/psRNATarget/getseq.do?sessionid=1424358326993263&source=target&seqID=TC486115) | 4.5 | 17.035 | rRF 24 UAUAGGCAACGGCUCUCAGUAACA 1  :: ::.::: :.:: .::..::::  Target 360 AUUUCUGUUCCUGAAGGUUGUUGU 383 | Ribulose bisphosphate carboxylase/oxygenase activase, | Translation |
|  |  | [TC431385](http://plantgrn.noble.org/psRNATarget/getseq.do?sessionid=1424358326993263&source=target&seqID=TC431385) | 4.5 | 20.794 | rRF 24 UAUAGG-CAACGGCUCU--CAGUAACA 1  :::::. :::::.:::: ::::::::  Target524 AUAUCUUGUUGCUGAGAAGGUCAUUGU550 | Heat shock protein 90 | Translation |
|  |  | [TC464921](http://plantgrn.noble.org/psRNATarget/getseq.do?sessionid=1424358326993263&source=target&seqID=TC464921) | 4.5 | 10.253 | rRF 24 UAUAGGCAACGGCUCUCAGUAACA 1  ::: :: :: ::::: ::.::::  Target 226 AUAGCCUUUACCGAGCUUCGUUGU 249 | Glycine-rich RNA-binding protein | Translation |
|  |  | [NP213096](http://plantgrn.noble.org/psRNATarget/getseq.do?sessionid=1424358326993263&source=target&seqID=NP213096) | 5.0 | 16.073 | rRF 24 UAUAGGCAACGGCUCUCAGUAACA 1  .:::.::::::: . :: .:::::  Target 183 GUAUUCGUUGCCUGAAGAUAUUGU 206 | Disease resistance-like protein | Translation |
|  |  | [BE209469](http://plantgrn.noble.org/psRNATarget/getseq.do?sessionid=1424358326993263&source=target&seqID=BE209469) | 5.0 | 13.204 | rRF 24 UAUAGGCAACGGCUCUCAGUAACA 1  ::: :::: ::::::. : ::::  Target 197 AUAGCCGUGGCCGAGGCUAAUUGC 220 | Protein kinase G11A | Cleavage |
|  |  | [TC487072](http://plantgrn.noble.org/psRNATarget/getseq.do?sessionid=1424358326993263&source=target&seqID=TC487072) | 5.0 | 17.556 | rRF 24 UAUAGGCAACGGCUCUCAGUAACA 1  .::: ::: .:::.::::::::  Target 211 GUAUGAAUUGAUGAGGGUCAUUGU234 | Beta-tubulin 7 | Cleavage |
|  |  | [TC432934](http://plantgrn.noble.org/psRNATarget/getseq.do?sessionid=1424358326993263&source=target&seqID=TC432934) | 5.0 | 13.603 | rRF 23 AUAGGCAACGGCUCUCAGUAACA 1  :.:..:::: .:: ::.:::::  Target 351 UGUUUGUUGGUGAUUGUUAUUGU 373 | Mitotic cyclin a2-type | Translation |
|  |  | [TC486105](http://plantgrn.noble.org/psRNATarget/getseq.do?sessionid=1424358326993263&source=target&seqID=TC486105) | 5.0 | 21.947 | rRF 24 UAUAGGCAACGGCUCUCAGUAACA 1  ::. :.::::..: :: ::::::  Target1149 AUGGCUGUUGUUGUCAGACAUUGU 1172 | CBS domain-containing protein | Translation |
|  |  | [TC423116](http://plantgrn.noble.org/psRNATarget/getseq.do?sessionid=1424358326993263&source=target&seqID=TC423116) | 5.0 | 20.415 | rRF 24 UAUAGG-CAACGGCUCU--CAGUAACA 1  :::::. :::::::: : ::::::::  Target76 AUAUCUUGUUGCCGAAAAGGUCAUUGU 502 | Heat shock protein 90 | Translation |
| ***Solanum tuberosum*** | *Solanum tuberosum* unigene, DFCI Gene Index (STGI), version 13 | [TC202514](http://plantgrn.noble.org/psRNATarget/getseq.do?sessionid=1424345816882570&source=target&seqID=TC202514) | 2.5 | 17.739 | rRF 21 GGCAACGGCUCUCAGCAAACA 1  ..:::::.:::: :.::::::  Target 621 UUGUUGCUGAGACUUGUUUGU 641 | CMV 1a interacting protein 1 | Translation |
|  |  | [TC209130](http://plantgrn.noble.org/psRNATarget/getseq.do?sessionid=1424345816882570&source=target&seqID=TC209130) | 2.5 | 11.833 | rRF 21 GGCAACGGCUCUCAGCAAACA 1  ..:::::.:::: :.::::::  Target 1283 UUGUUGCUGAGACUUGUUUGU 1303 | CMV 1a interacting protein 1 | Translation |
|  |  | [AM906435](http://plantgrn.noble.org/psRNATarget/getseq.do?sessionid=1424346440763084&source=target&seqID=AM906435) | 3.5 | 22.4 | rRF 21 GGCAACGGCUCUCAGCAAACA 1  .::::::. ::: :::::::  Target 180 UCGUUGCUUCGAGGCGUUUGU 200 | Mannose-P-dolichol utilization defect 1b | Cleavage |
|  |  | [TC217879](http://plantgrn.noble.org/psRNATarget/getseq.do?sessionid=1424346440763084&source=target&seqID=TC217879) | 4.0 | 22.396 | rRF 25 UAUAGGCAACGGCUCUCAGCAAACA 1  :: .:: : .:.:::::::::::  Target 60 AUCGUCGGAGGUGGGAGUCGUUUGU 84 | ATP-dependent RNA helicase-like protein DB10 | Cleavage |
|  |  | [EG015628](http://plantgrn.noble.org/psRNATarget/getseq.do?sessionid=1424346440763084&source=target&seqID=EG015628) | 4.5 | 13.801 | rRF 20 GCAACGGCUCUCAGCAAACA 1  .::::..:::: :.:: :::  Target 481 UGUUGUUGAGAUUUGUCUGU 500 | Vacuolar processing enzyme-1b | Translation |
|  |  | [CN516922](http://plantgrn.noble.org/psRNATarget/getseq.do?sessionid=1424346440763084&source=target&seqID=CN516922) | 5.0 | 11.622 | rRF 25 UAUAGGCAACGGCUCUCAGCAAACA 1  .:.:::::: :: ::::.::: ::  Target 57 GUGUCCGUUUCCUUGAGUUGUUGGU 81 | ABC transporter related | Cleavage |
|  |  | [CN515612](http://plantgrn.noble.org/psRNATarget/getseq.do?sessionid=1424346440763084&source=target&seqID=CN515612) | 5.0 | 17.784 | rRF 23 UAGGCAACGGCUCUCAGCAAACA 1  .::.::: :.::::: .::: ::  Target 559 GUCUGUUUCUGAGAGGUGUUGGU 581 | 60S ribosomal protein L21-like protein | Cleavage |
|  |  | [CV429819](http://plantgrn.noble.org/psRNATarget/getseq.do?sessionid=1424346440763084&source=target&seqID=CV429819) | 5.0 | 21.288 | rRF 24 AUAGGCAACGGCUCUCAGCAAACA 1  :. :: ::::. :::::: ::::  Target 578 UGGCCAUUGCUAAGAGUCCUUUGG601 | 4-coumarate:coenzyme A ligase | Cleavage |
|  |  | [DV625227](http://plantgrn.noble.org/psRNATarget/getseq.do?sessionid=1424346440763084&source=target&seqID=DV625227) | 5.0 | 21.277 | rRF 20 GCAACGGCUCUCAGCAAACA 1  :::::::.::: :::: :  Target 343 AGUUGCCGGGAGACGUUCGG 362 | SBP transcription factor | Cleavage |

**Supplementary Table 2 :** List of high throughput sRNA datasets of plants used in the study

| **Plant group** | **Plant Species** | **GEO Accession No** | **Tissue** | **Platform** | **References** |
| --- | --- | --- | --- | --- | --- |
| Dicots | *Solanum tuberosum* | GSM803582, GSM803583,GSM803584 | Leaves, Flowers, Stolon | Illumina Genome Analyzer | Chavez Montes et al., 2014 |
|  | *Solanum lycopersicum* | GSM803579, GSM803580, GSM803581 | Leaves, Flowers, Fruit | Illumina Genome Analyzer |  |
|  | *Nicotiana tabacum* | GSM717860, GSM717861, GSM717862 | Leaves, Male Flowers, Pods | Illumina Genome Analyzer |  |
|  | *Capsicum annuum* | GSM712519, GSM712520, GSM712521 | Leaves, Flowers, Fruits | Illumina Genome Analyzer |  |
|  | *Mimulus guttattus* | GSM717696, GSM717697, GSM717698 | Leaves, Flowers, Roots | Illumina Genome Analyzer |  |
|  | *Silene latifolia* | GSM803576, GSM803577, GSM803578 | Leaves, Flowers, Smutted Flowers | Illumina Genome Analyzer |  |
|  | *Populus trichocarpa* | GSM717875, GSM717876 | Leaves, Xylem | Illumina Genome Analyzer |  |
|  | *Cucurbita maxima* | GSM717552, GSM717553, GSM717554 | Leaves, Flowers, Phloem sap | Illumina Genome Analyzer |  |
|  | *Medicago truncatula* | GSM769277, GSM769273, GSM769275 | Leaves, root, flower, Nodules | Illumina Genome Analyzer | Zhai et al., 2011 |
|  | *Arachis hypogaea* | GSM769280, GSM769281 | Nodules, Flower | Illumina Genome Analyzer |  |
|  | *Glycine max* | GSM769282, GSM769283, GSM769284, GSM769285 | Root, Nodule, Flower, Developing Seed | Illumina Genome Analyzer |  |
|  | *Arabidopsis thaliana* | GSM707678, GSM707679, GSM707680 | Flowers, Leaves, Roots | Illumina Genome Analyzer | Wang et al., 2011 |
|  | *Gossipium arboreum* | GSM717570, GSM717571,GSM717572 | Leaves, Flowers, Fibres | Illumina Genome Analyzer | Chavez Montes et al., 2014 |
|  | *Citrus sinensis* | GSM712528, GSM712529,GSM712530 | Leaves, Flowers, Fruits | Illumina Genome Analyzer |  |
|  | *Carica papaya* | GSM712525, GSM712526, GSM712527 | Leaves, Flowers, Infected Leaves | Illumina Genome Analyzer |  |
|  | *Vitis vinifera* | GSM803800, GSM803801, GSM803802 | Leaves, Flowers, Berries | Illumina Genome Analyzer |  |
| Monocots | *Oryza sativa* | GSM816687,GSM816705,GSM816719,  GSM816732 | Seedlings, Root, Shoot, Panicle | Illumina Genome Analyzer | Jeong et al., 2011 |
|  | *Hordeum vulgare* | GSM717573, GSM717574,GSM717575 | Leaves, Inflorescence, Blumeria infected leaf | Illumina Genome Analyzer | Chavez Montes et al., 2014 |
|  | *Zea mays* | GSM433620, GSM433621,GSM433622 | Leaves, Ears, Tassels | Illumina Genome Analyzer |  |
|  | *Setaria italica* | GSM803573, GSM803574,GSM803575 | Leaves, Flowers, Roots | Illumina Genome Analyzer |  |
|  | *Sorghum bicolor* | GSM803128, GSM803129,GSM803130 | Leaves, Flowers, Stolon | Illumina Genome Analyzer |  |
|  | *Triticum aestivum* | GSM803792, GSM803793, GSM803794 | Leaves, Spikelets, Infected Spikelets | Illumina Genome Analyzer |  |
|  | *Panicum virgatum* | GSM717866, GSM717867, GSM717868 | Leaves, Flowers, Drought treated Leaves | Illumina Genome Analyzer |  |
|  | *Miscanthus giganteus* | GSM501370,GSM501371, GSM501372 | Leaves, Flowers, Rhizomes | Illumina Genome Analyzer |  |
|  | *Musa acuminata* | GSM717699, GSM717700, GSM717701 | Leaves, Male Flowers, Immature Fruit | Illumina Genome Analyzer |  |
| Magnoliids | *Persea americana* | GSM717869,GSM717870,GSM717871 | Leaves, Flowers, Ripening Fruit | Illumina Genome Analyzer |  |
|  | *Piper nigrum* | GSM1606153,GSM1606154, GSM1606155 | Leaves, Infected leaf, Infected Root | Illumina Genome Analyzer | Under communication |
|  | *Aristolochia fimbriata* | GSM712072, GSM712073 | Leaves, Flower Buds | Illumina Genome Analyzer | Chavez Montes et al., 2014  Chavez Montes et al., 2014 |
| Lower angiosperms | *Nuphar advena* | GSM717863,GSM717864,GSM717865 | Leaves, Flowers, Roots | Illumina Genome Analyzer |  |
|  | *Amborella sp* | GSM712477 | Leaves | Illumina Genome Analyzer |  |
| Gymnosperm | *Cycas rumphiis* | GSM717555,GSM717556, GSM717557 | Leaves, Ovules, Microsporangia | Illumina Genome Analyzer |  |
|  | *Picea abies* | GSM717872, GSM717873 | Leaves, Flowers | Illumina Genome Analyzer |  |
|  | *Ginkgo biloba* | GSM717559,GSM717560, GSM717561 | Leaves, Female Cones, Male Cones | Illumina Genome Analyzer |  |
| Pteridophyte (Ferns) | *Marsilea quadrifolia* | GSM717693, GSM717694 | Leaves, Roots | Illumina Genome Analyzer |  |
| Chlorophyta  (Green algae) | *Chara corallina* | GSM712522 | Thallus | Illumina Genome Analyzer |  |
|  | *Chlamydomonas reinhardtii* | GSM803103 | - | Illumina Genome Analyzer |  |
|  | *Volvox carteri* | GSM803797 | - | Illumina Genome Analyzer |  |

**Supplementary Table 3**. The genebank accessions of rRNA sequences of plants used in the study

| **Plant species** | **Genbank Accesssion** | **Plant species** | **Genbank Accesssion** |
| --- | --- | --- | --- |
| *Aristolochia sp* | gi|222062916|emb|AM501927.1| | *Carica papaya* | gi|401787231|gb|JX092051.1| |
| *Nuphar advena* | gi|209418406|emb|FM242145.1| | *Citrus sinensis* | gi|392313558|gb|JQ990165.1| |
| *Persea americana* | gi|240248190|emb|FM957821.1| | *Gossypium arboreum* | gi|530831|gb|U12712.1|GAU12712 |
| *Piper nigrum* | gi|13925819|gb|AF275198.1| | *Medicago truncatula* | gi|8132842|gb|AF233339.1| |
| *Cycas rumphii* | gi|21623588|dbj|AB076205.1| | *Arachis hypogaea* | gi|314910732|gb|HQ537458.1| |
| *Ginkgo biloba* | gi|75993658|gb|DQ191445.1 | *Glycine max* | gi|5912443|emb|AJ011337.1| |
| *Picea glauca* | gi|4574780|gb|AF136621.1| | *Populus trichocarpa* | gi|5912284|emb|AJ006440.1| |
| *Panicum virgatum* | gi|68164129|gb|DQ005062.1| | *Cucurbita maxima* | gi|225698853|emb|AM981171.1| |
| *Hordeum vulgare* | gi|698945885|gb|KM217265.1| | *Silene latifolia* | gi|313718373|emb|FN821133.1| |
| *Sorghum bicolor* | gi|299831205|gb|GQ856358.1| | *Mimulus guttatus* | gi|46392996|gb|AY575439.1| |
| *Triticum aestivum* | gi|17016972|gb|AF438188.1|AF438188 | *Solanum lycopersicum* | gi|216409752|dbj|AB373816.1| |
| *Oryza sativa* | gi|86211221|gb|DQ355274.1| | *Solanum tuberosum* | gi|573016788|gb|KF022370.1| |
| *Zea mays* | gi|3282440|gb|AF019817.1| | *Nicotiana tabacum* | gi|4582191|emb|AJ012367.1| |
| *Setaria italica* | gi|459256896|gb|KC201689.1| | *Capsicum annuum* | gi|294845721|gb|GU944973.1| |
| *Musa acuminata* | gi|312284045|emb|FR727954.1| | *Marsilea quadrifolia* | gi|33317275|gb|AF448792.1| |
| *Miscanthus x giganteus* | gi|18652835|emb|AJ426563.1| | *Arabidopsis thaliana* | gi|6686789|emb|AJ232900.1 |
| *Vitis vinifera* | gi|671390052|gb|KF544886.1| |  |  |

**Supplementary Table 4**. Details of the AGO immunoprecipitated small RNA library used in the study

| **Plant** | **GEO Accession No** | **ARGONAUTE Association** | **Tissue** | **Total reads** | **Sequencing method** | **References** |
| --- | --- | --- | --- | --- | --- | --- |
| *Arabidopsis thaliana* | GSM707682 | AGO1 | Flower | 4,000,470 | Illumina | Wang et al., 2011 |
|  | GSM707683 | AGO1 | Leaf | 2,786,268 |  |  |
|  | GSM707684 | AGO1 | Root | 2,544,588 |  |  |
|  | GSM707685 | AGO1 | Seedling | 4,845,886 |  |  |
|  | GSM707686 | AGO4 | Flower | 4,112,230 |  |  |
|  | GSM707687 | AGO4 | Leaf | 3,885,991 |  |  |
|  | GSM707688 | AGO4 | Root | 3,741,619 |  |  |
|  | GSM707689 | AGO4 | Seedling | 4,233,673 |  |  |
|  | GSM304282 | AGO2 | Flower | 687,236 | Illumina | Montgomery et al., 2008 |
|  | GSM304283 | AGO2 | Flower | 1,467,452 |  |  |
|  | GSM304284 | AGO7 | Flower | 272,641 |  |  |
|  | GSM304285 | AGO7 | Flower | 822,542 |  |  |
|  | GSM415789 | AGO6 | Flower | 1,523,909 | Illumina | Havecker et al., 2010 |
|  | GSM415790 | AGO6 | Flower | 1,280,536 |  |  |
|  | GSM415791 | AGO9 | Flower | 2,204,544 |  |  |
|  | GSM415792 | AGO9 | Flower | 2,906,787 |  |  |
| *Oryza sativa* | GSM455962 | AGO1a | Seedling | 3,791,013 | Illumina | Wu et al., 2009 |
|  | GSM455963 | AGO1b | Seedling | 1,230,668 |  |  |
|  | GSM455964 | AGO1c | Seedling | 3,891,687 |  |  |
|  | GSM455965 | AGO1_total | Seedling | 4,029,462 |  |  |

The GSM707682-GSM707685 represents the AGO1 immunoprecipitated and two-step purified (TSP) small RNAs from flowers, leaves, roots and seedlings of the transgenic Arabidopsis plants expressing FLAG-AGO1^33^. GSM707686-GSM707689 represents the AGO4 immunoprecipitated and two-step purified (TSP) small RNAs from flowers, leaves, roots and seedlings of the transgenic Arabidopsis plants expressing FLAG-AGO4^33^. GSM304282 and GSM304283consisted of AGO2 immunoprecipitated and co- immunoprecipitated small RNAs from the flower tissue (stages 1–12) of Col-0 AGO2:HA-AGO2 transformed Arabidopsis plants^31^. Whereas GSM304284-GSM304285 includes AGO7 immunoprecipitated small RNAs from the flower tissues of zip-1 AGO7:HA-AGO7 transformed Arabidopsis plants^31^. GSM415789 andGSM415790 were FLAGAGO6 immunoprecipitated small RNA datasets of Arabidopsis plant with FLAG epitope–tagged genomic construct that complements the ago6-2 mutation and GSM415791 and GSM415792 represents the small RNA library immunoprecipitated with endogenous AGO9 Antibody^32^. GSM455962, GSM455963 and GSM455964 represent the datasets of small RNAs immunoprecipitated with different AGO1 homologs, AGO1a, AGO1b and AGO1c respectively from *O. sativa*. GSM455965 represents the control AGO1 immunoprecipitated small RNAs^34^.

**Supplementary Table 5. The expression of different RPS13 mRNA transcripts among the stress responsive transcriptome of black pepper.** Pn_IL- *P. capsici* infected *P. nigrum* Leaf Transcriptome, Pn_IR- *P. capsici* infected root Transcriptome, Pn_CL- Control uninfected Leaf Transcriptome. Total raw fragments in Pn_IL, Pn_IR and Pn_CL transcriptome were respectively, 22432586, 16789220 and 24645407.

| **Gene ID** | **Gene**  **Length** | **Raw fragments** | | | **FPKM** | | |
| --- | --- | --- | --- | --- | --- | --- | --- |
|  |  | **Pn_IL** | **Pn_IR** | **Pn_CL** | **Pn_IL** | **Pn_IR** | **Pn_CL** |
| **RPS13_1** | 822 | 627 | 629 | 870 | 34.0029 | 45.5773 | 42.9449 |
| **RPS13_2** | 823 | 494 | 414 | 1637 | 26.7576 | 29.9619 | 80.7073 |
| **RPS13_3** | 828 | 156 | 159 | 221 | 8.3988 | 11.4376 | 10.8299 |
| **RPS13_4** | 935 | 244 | 180 | 340 | 11.6332 | 11.4665 | 14.7981 |

**Supplementary Figure 1. Size distribution of srRNAs (ribosomal RNA derived small RNAs) in *Piper nigrum* sRNA libraries. Bars represent the percentage of reads in each length groups**


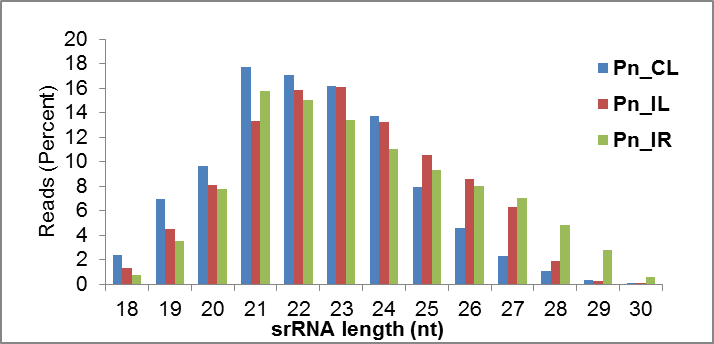


**Supplementary Figure 2. The integrity of RNA samples used for the sRNA library preparation assessed on the Agilent 2100 bioanalyzer**


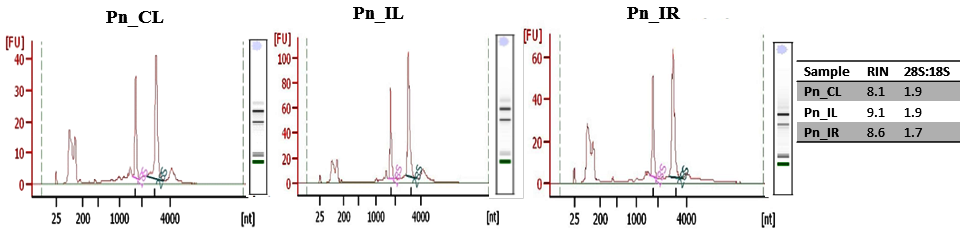


**Supplementary Figure 3(A). The 5.8SrRF variants (most abundant 10) from the leaf sRNA libraries of monocot plants**
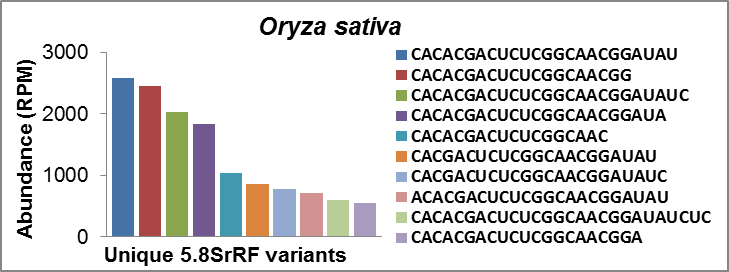

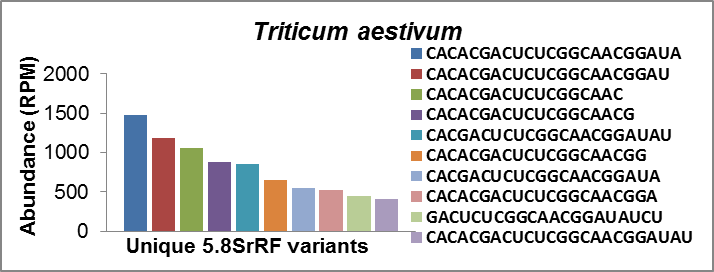

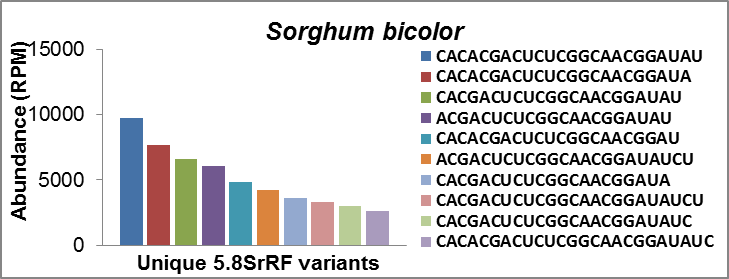

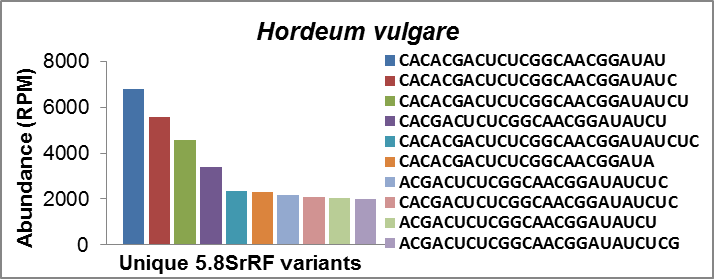

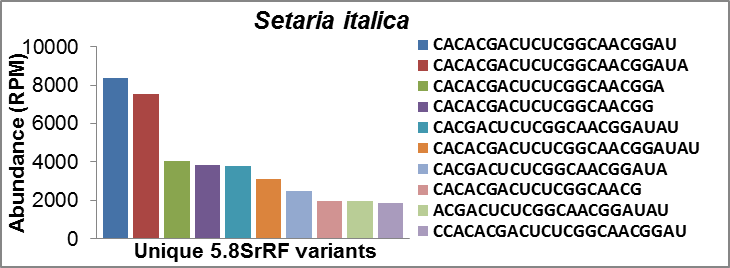

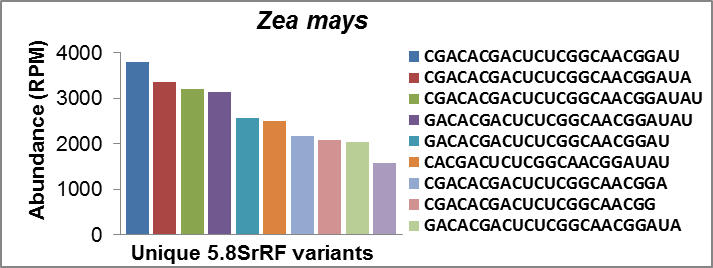

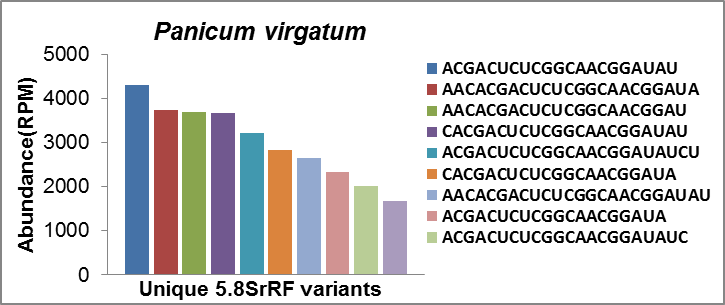

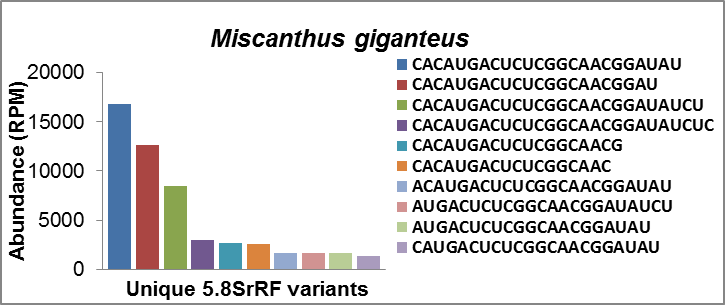

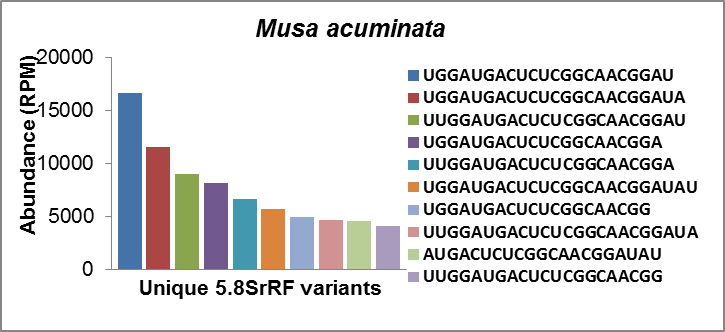


**Supplementary Figure 3(B). The abundant 5.8S rRF variants (most abundant 10) from the leaf sRNA libraries of magnoliid plants (*Aristolochia fimbriata*, *Persea Americana*) and lower angiosperm plants (*Nuphar advena* and *Amborella* sp)**


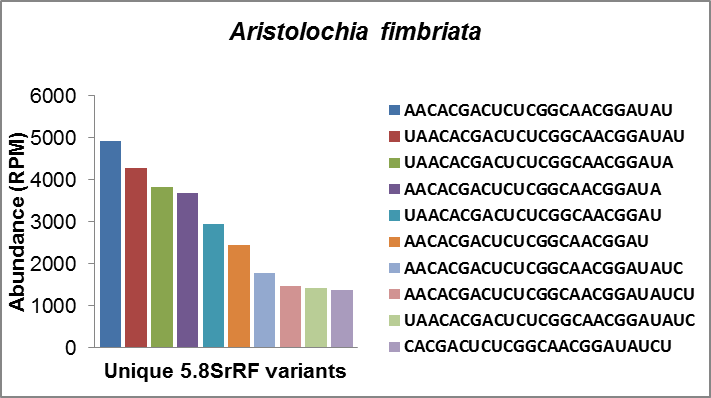

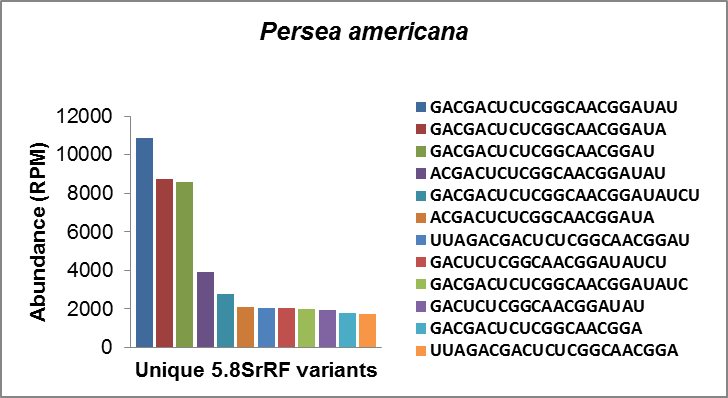

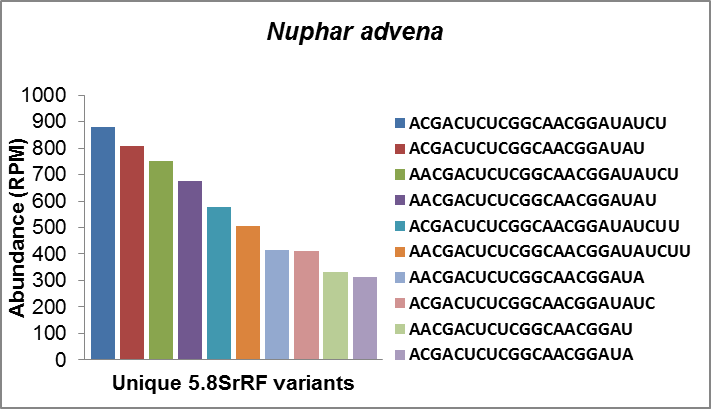

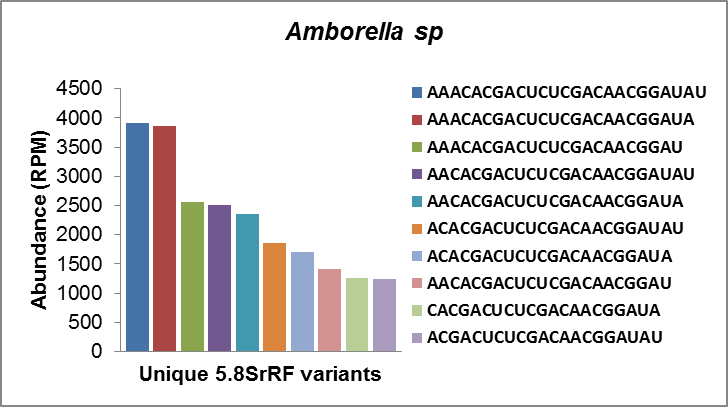


**Supplementary Figure 3(C). The 5.8S rRF variants (abundant 10) from the sRNA libraries of dicot plants**
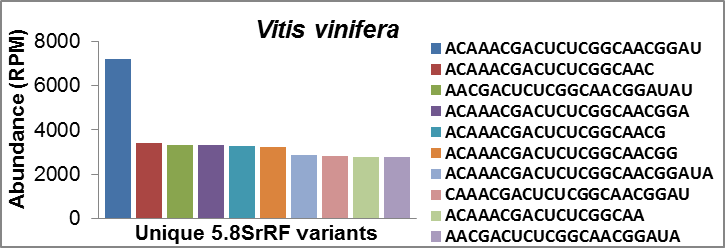

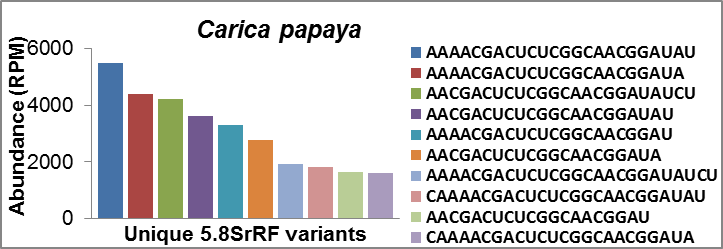

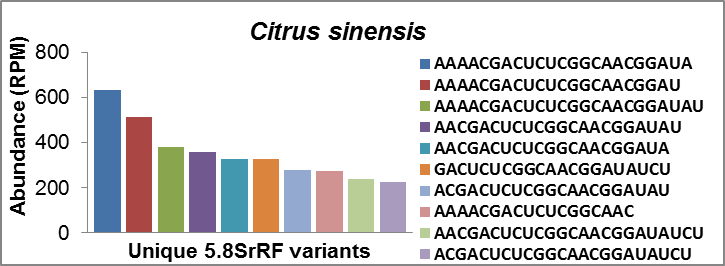

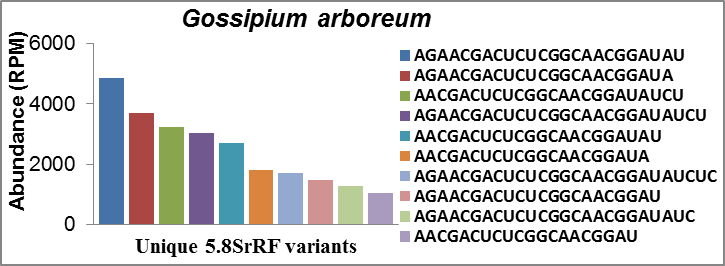

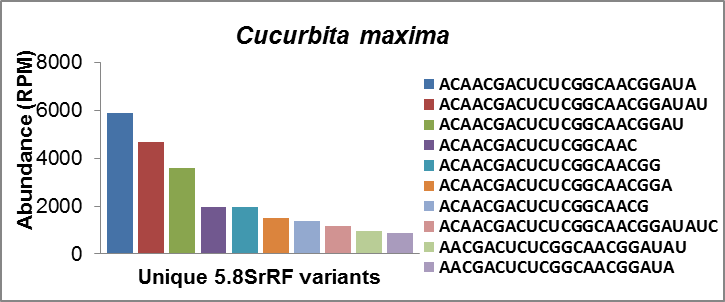

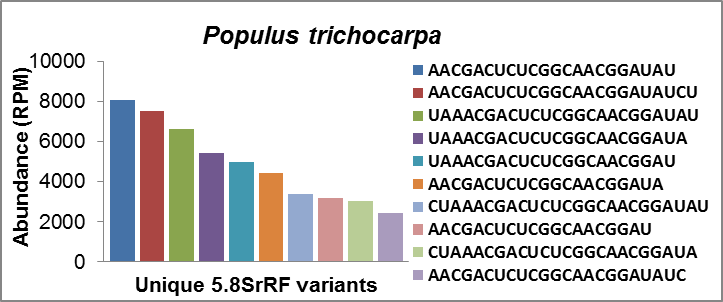


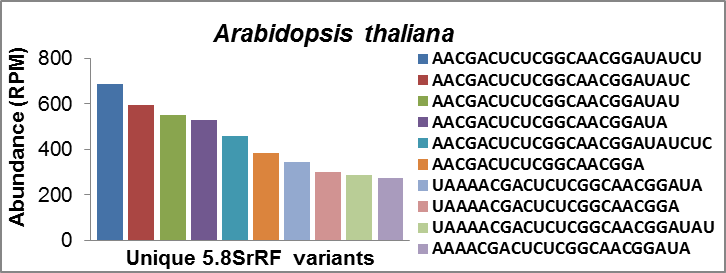

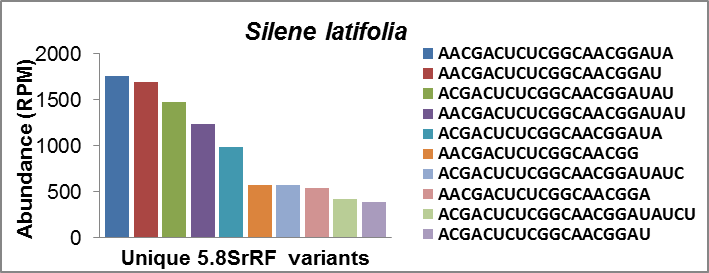

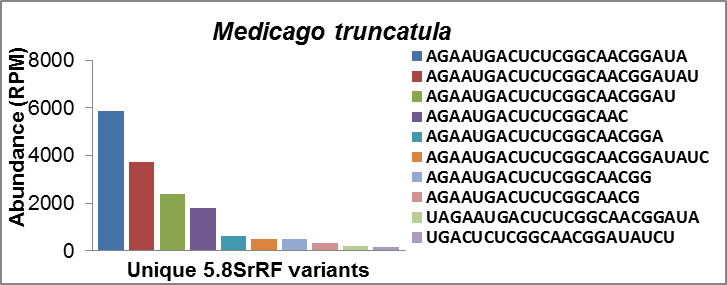

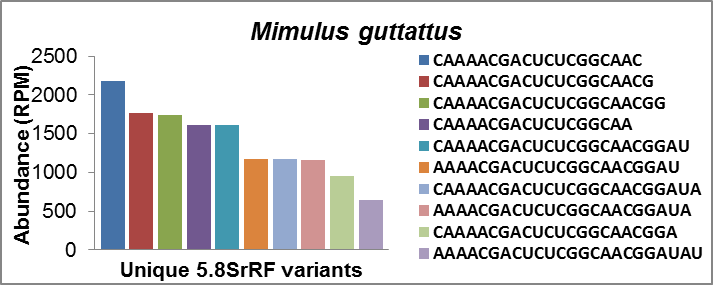

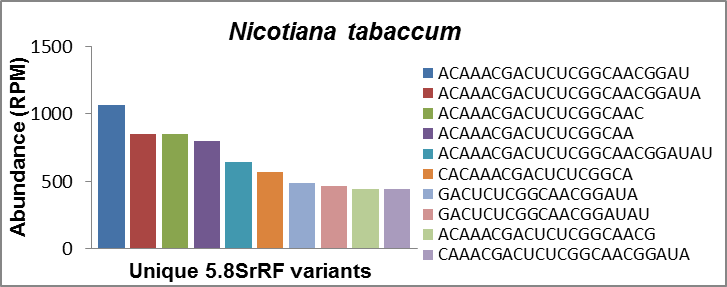

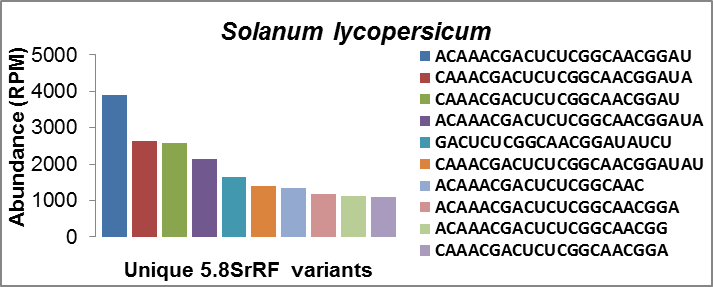


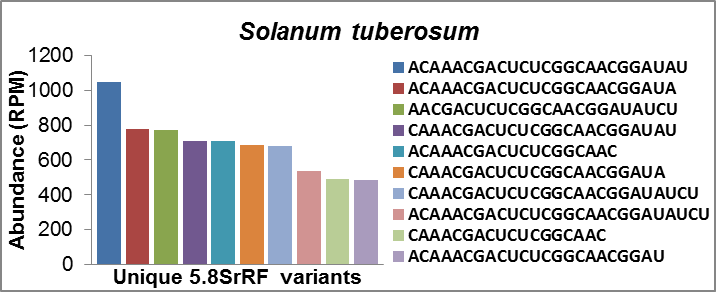

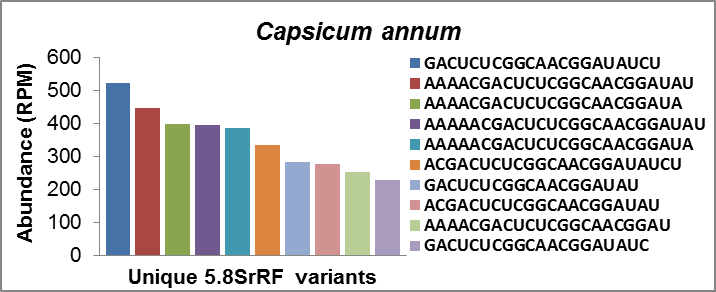


**Supplementary Figure 3(D). The abundant 5.8SrRF variants from the sRNA libraries of gymnosperm plants (*Cycas rumphii*, *Picea abies* and *Ginkgo biloba*)**


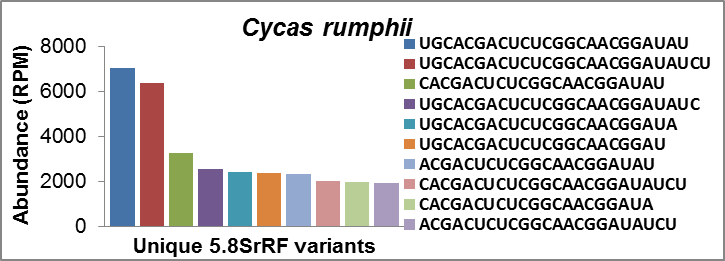

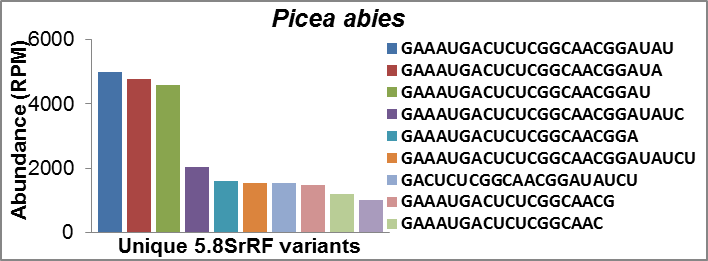

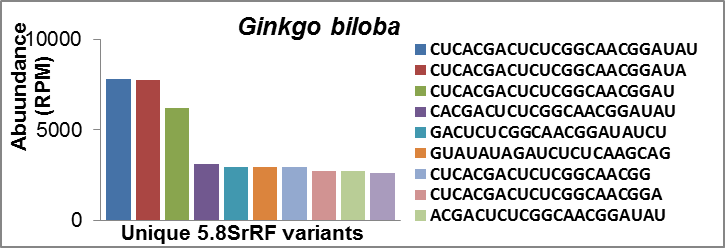


**Supplementary Figure 3(E). The abundant 5.8SrRF variants from the sRNA libraries of pteridophyte plant, *Marselia quadrifolia***


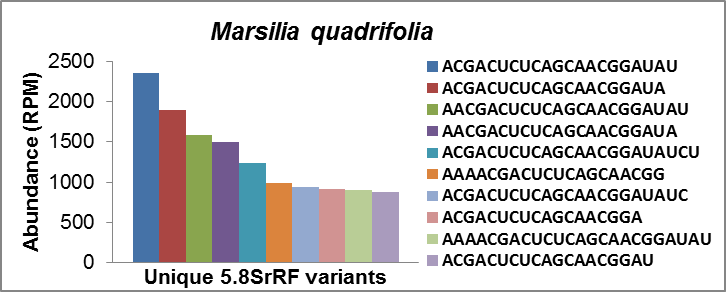


**Supplementary Figure 4(A). Cleavage pattern of 5' nucleotides of the 5′5.8SrRFs of the pteridophyte plant, *Marselia quadrifolia* assessed upstream of the ‘GACUCUC’ consensus**


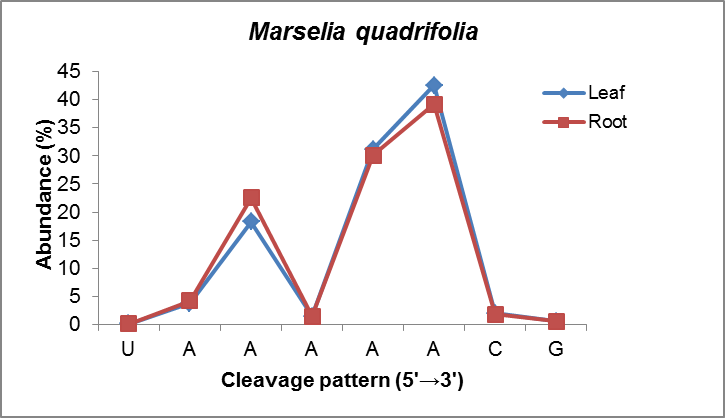


**Supplementary Figure 4(B). Cleavage pattern of 5' nucleotides of the 5'5.8S rRFs of the dicot plants assessed upstream of the ‘GACUCUC’ consensus**


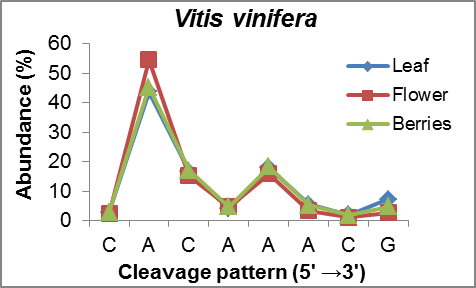

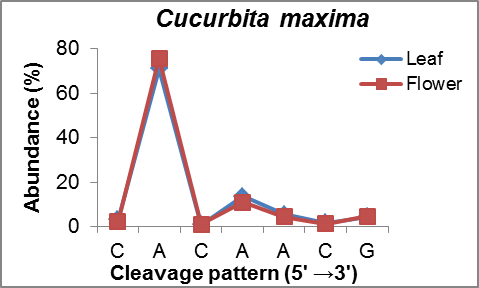

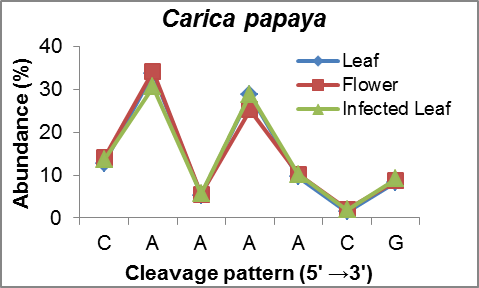

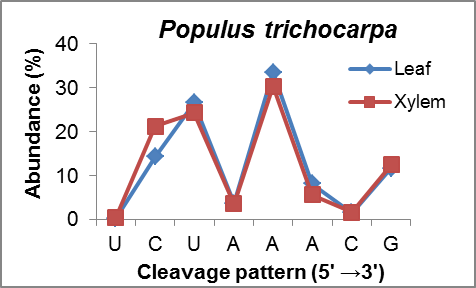

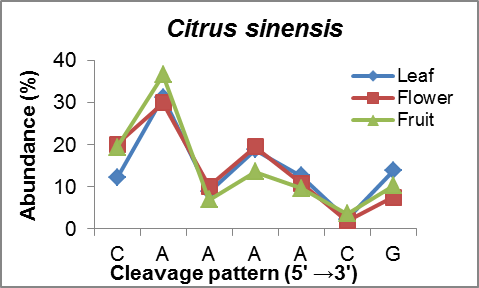

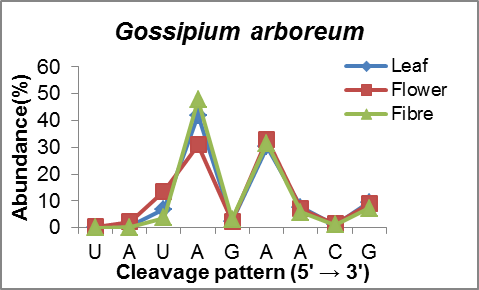

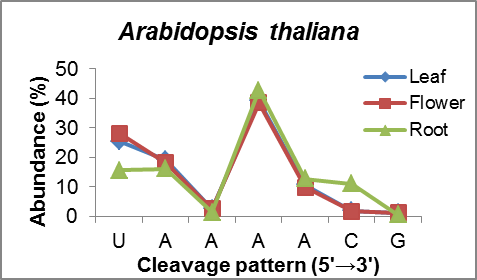

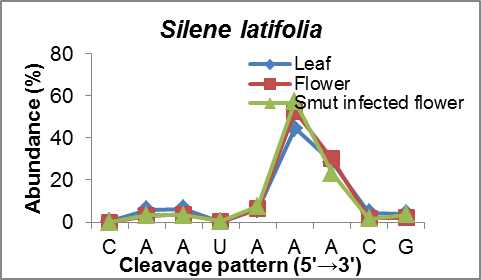

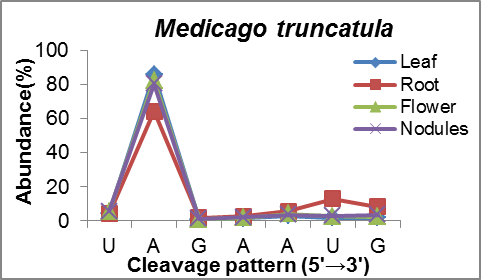


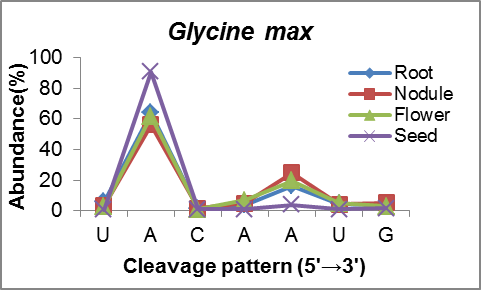

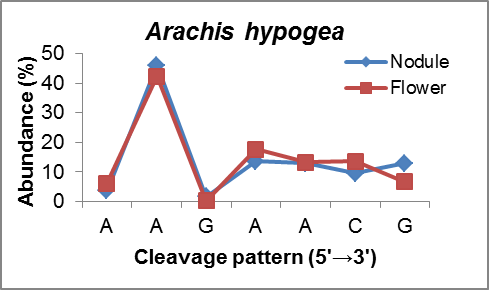

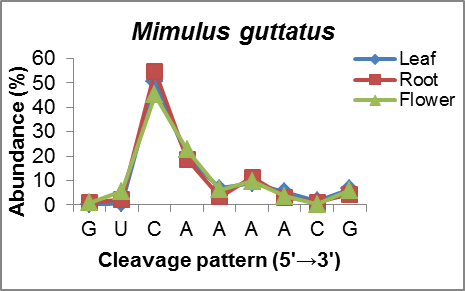

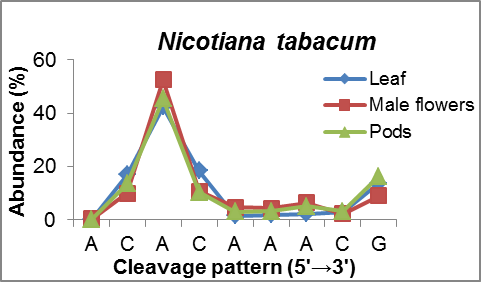

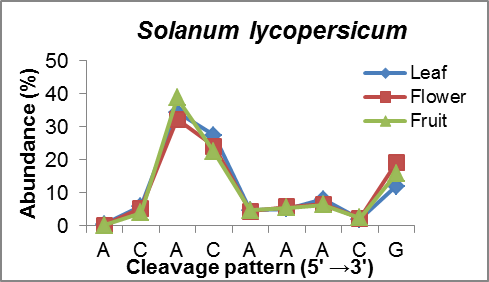

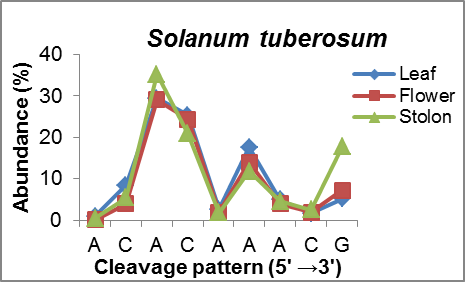

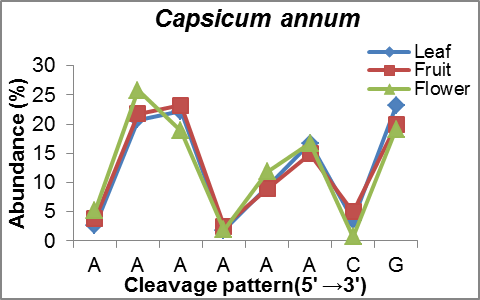


**Supplementary Figure 4 (C). Cleavage pattern of 5' nucleotides of the 5′ 5.8SrRFs of the monocot plants assessed upstream of the ‘GACUCUC’ consensus**


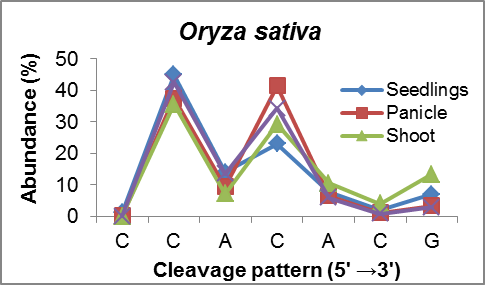

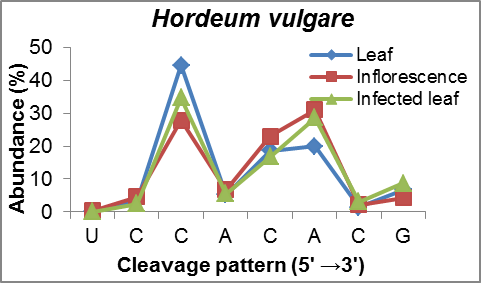

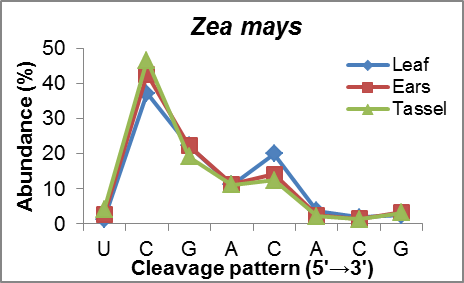

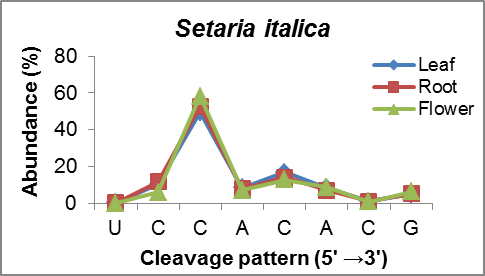

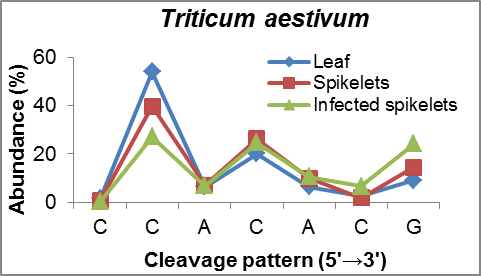

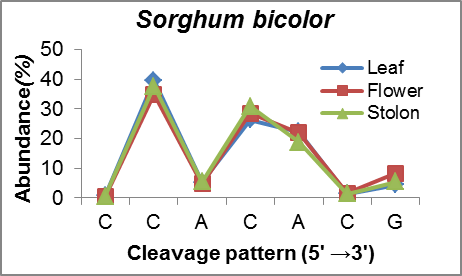

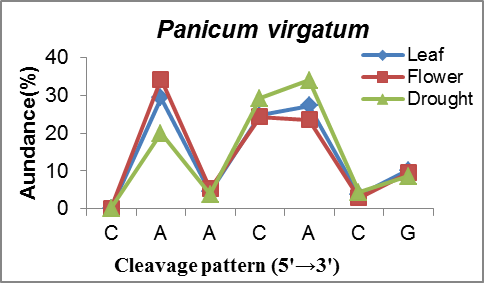

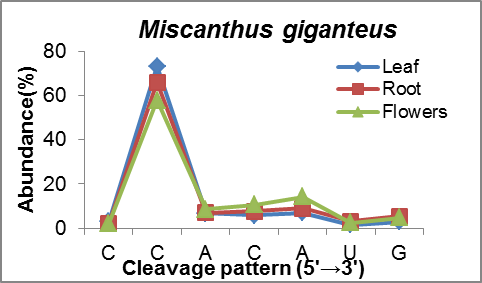

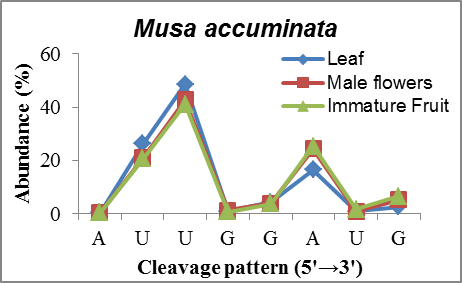


**Supplementary Figure 4(D) . Cleavage pattern of 5' nucleotides of the 5′ 5.8SrRFs of the magnoliid plants and the lower angiosperm plant *Nuphar advena* assessed upstream of the ‘GACUCUC’ consensus**


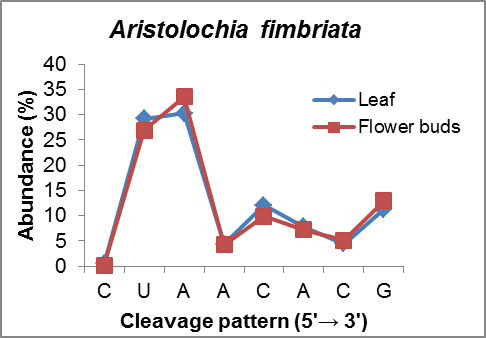

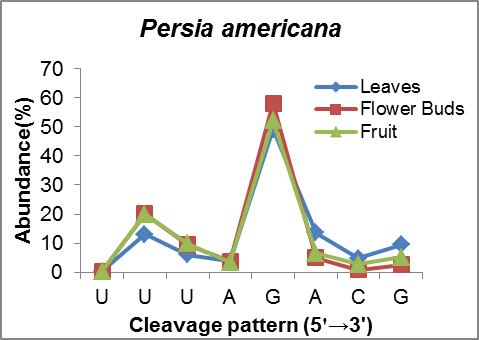

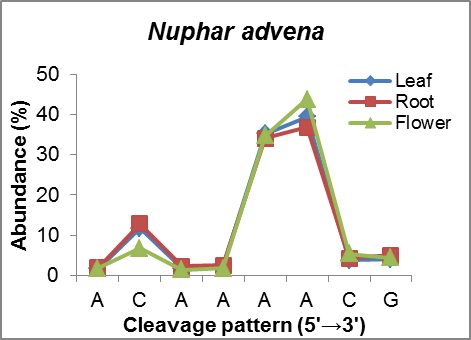


**Supplementary Figure. 4(E). Cleavage pattern of 5’ nucleotides of the 5’ 5.8s rRFs of the gymnosperm plants assessed upstream of the ‘GACUCUC’ consensus**


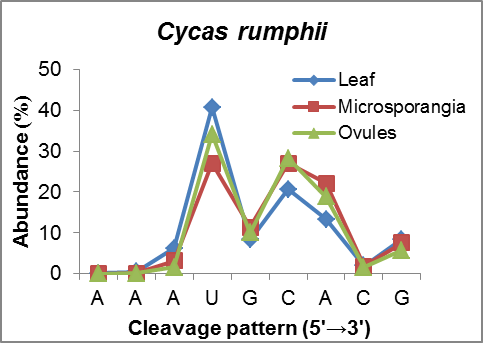

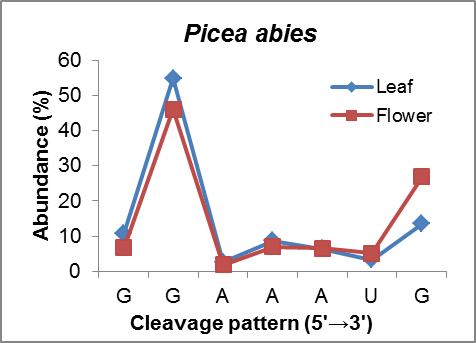

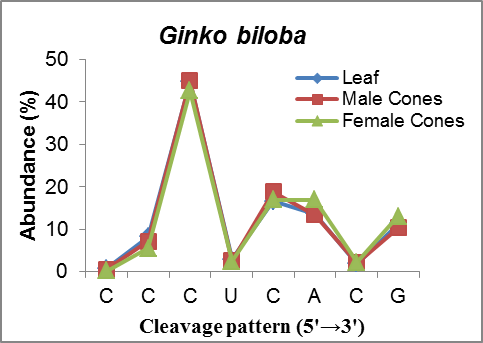


**Supplementary Figure 5(A). Length categorisation of 5′ 5.8SrRFs assessed upstream of the ‘GACUCUC’ consensus in dicots**


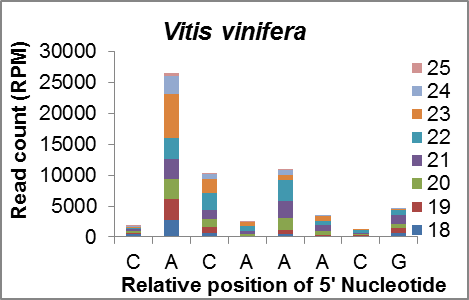

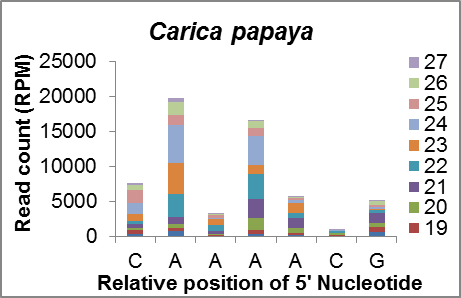

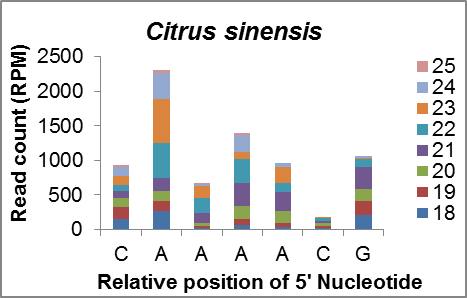

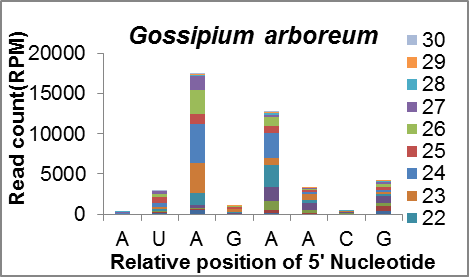

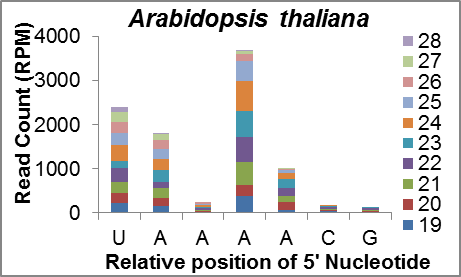

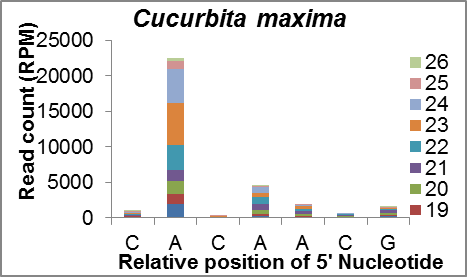

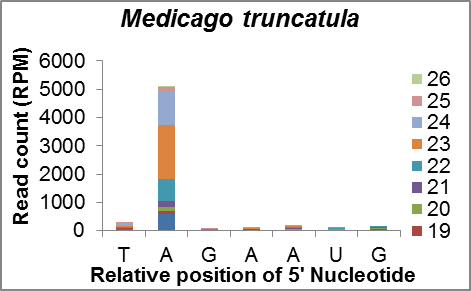

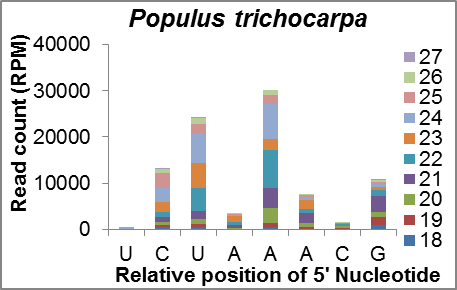

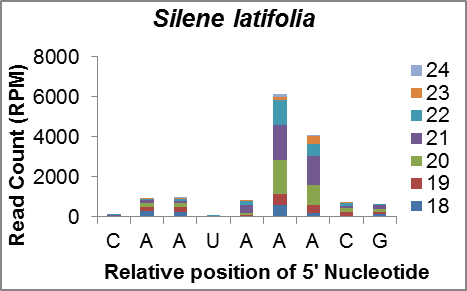


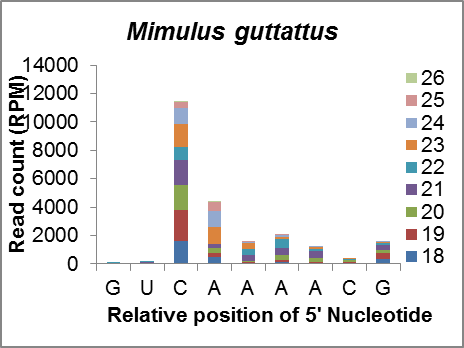

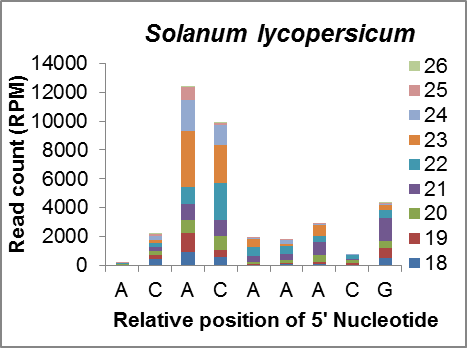

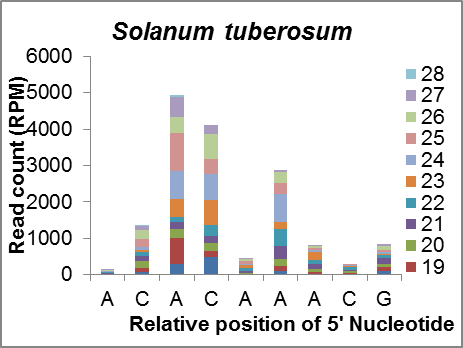

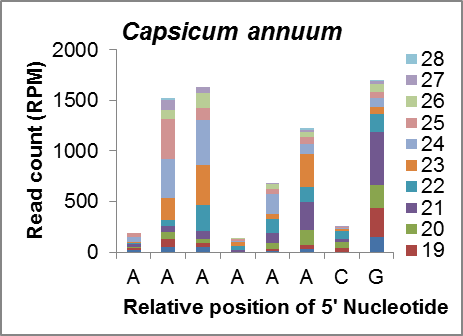

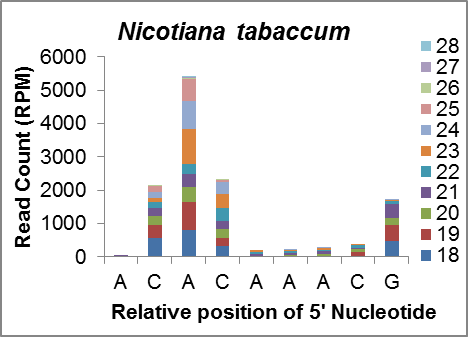


**Supplementary Figure 5(B). Length distribution of 5' 5.8SrRFs assessed upstream of the ‘GACUCUC’ consensus in the monocot plants**
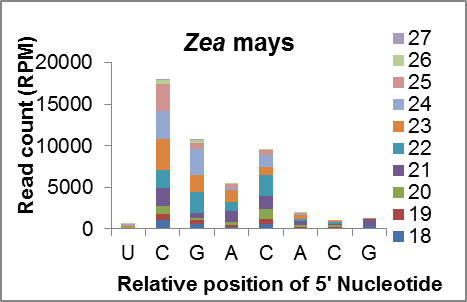

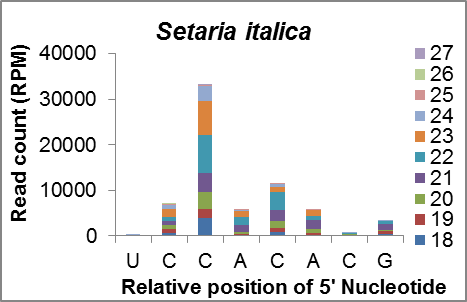

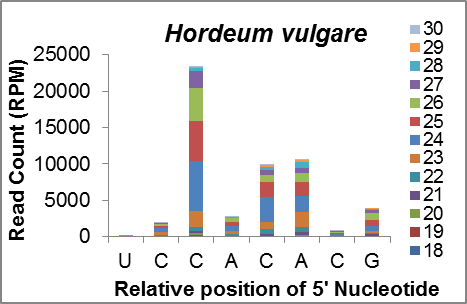

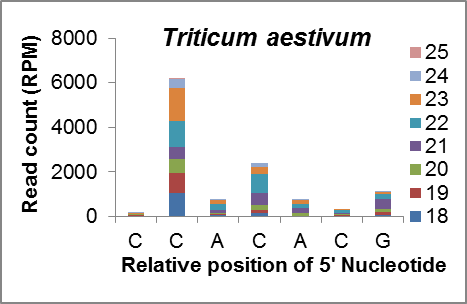

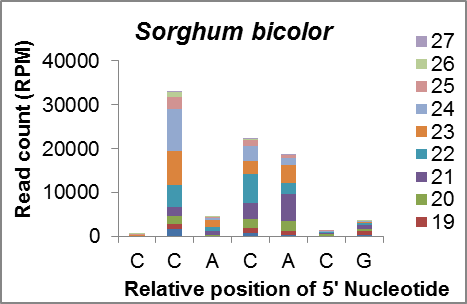

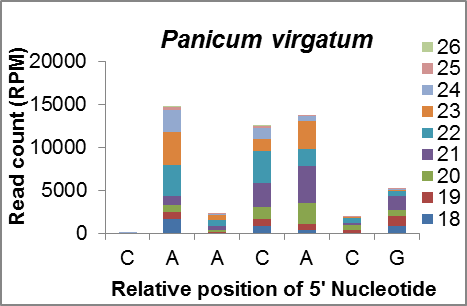
**
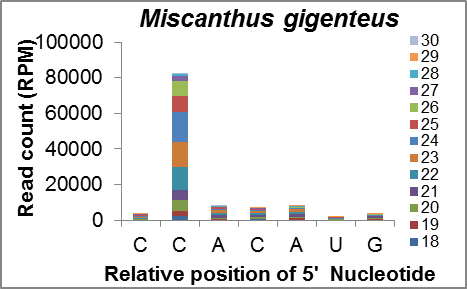
**
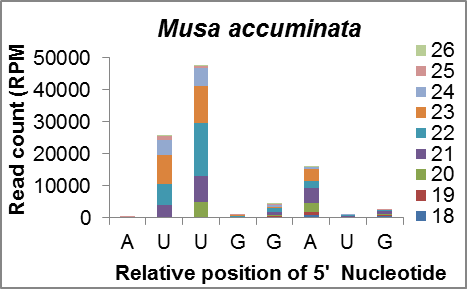


**Supplementary Figure 5(C). Length distribution of 5′ 5.8SrRFs assessed upstream of the ‘GACUCUC’ consensus in the magnoliid and other lower angiosperm plants**


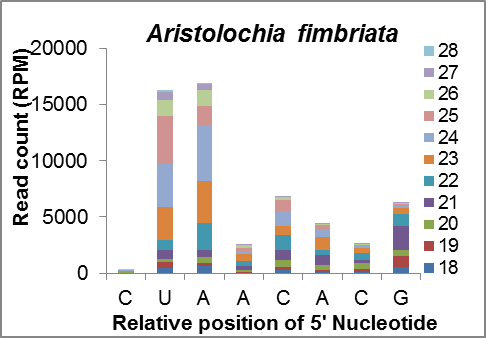

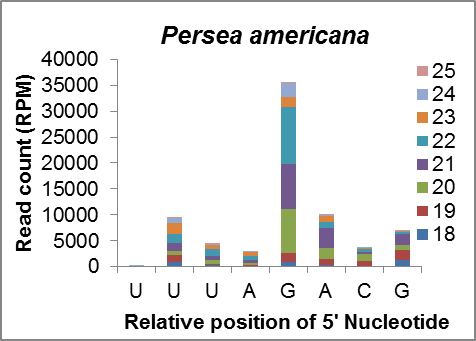

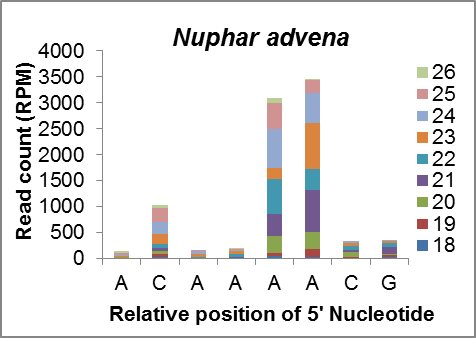

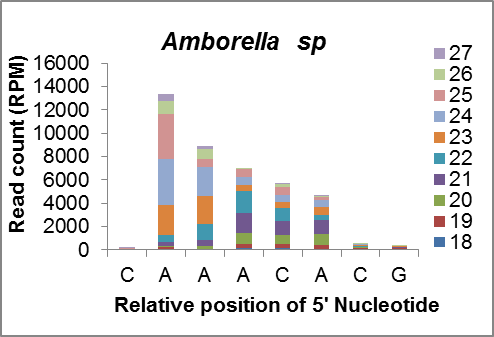


**Supplementary Figure 5(D). Length distribution of 5' 5.8SrRFs assessed upstream of the ‘GACUCUC’ consensus in the gymnosperm plants**


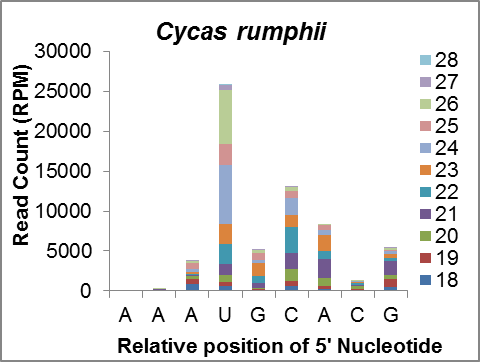

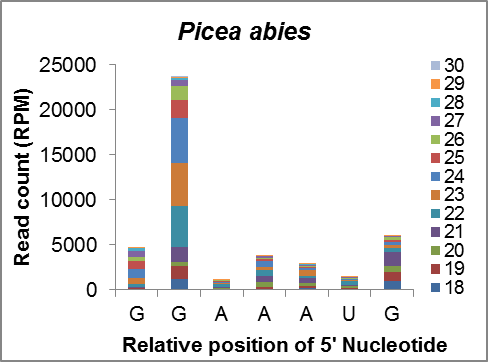

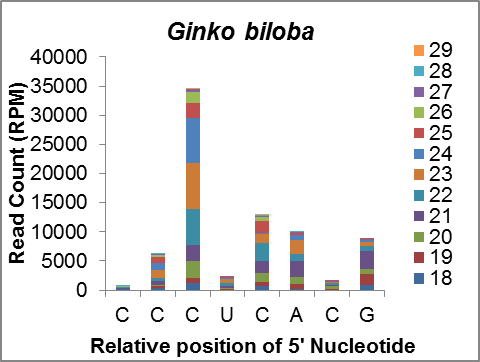


**Supplementary Figure 5(E). Length distribution of 5′5.8SrRFs assessed upstream of the ‘GACUCUC’ consensus in pteridophyte plant, *Marselia quadrifolia***


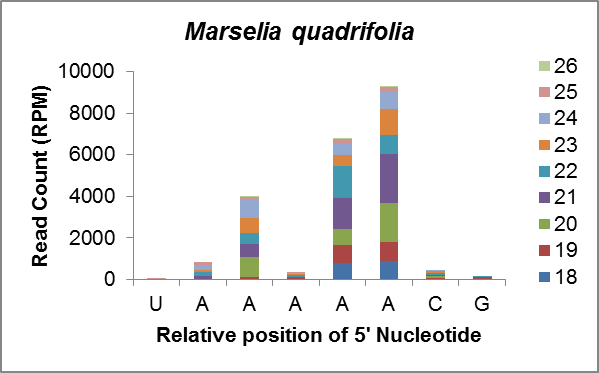


**Supplementary Figure 6. The proportion of 5'5.8S rRF reads in the leaf small RNA libraries of different plant species.** The 5′5.8S rRFs constitute an average of 4.33 percent of the total small RNA reads in the sRNA libraries.


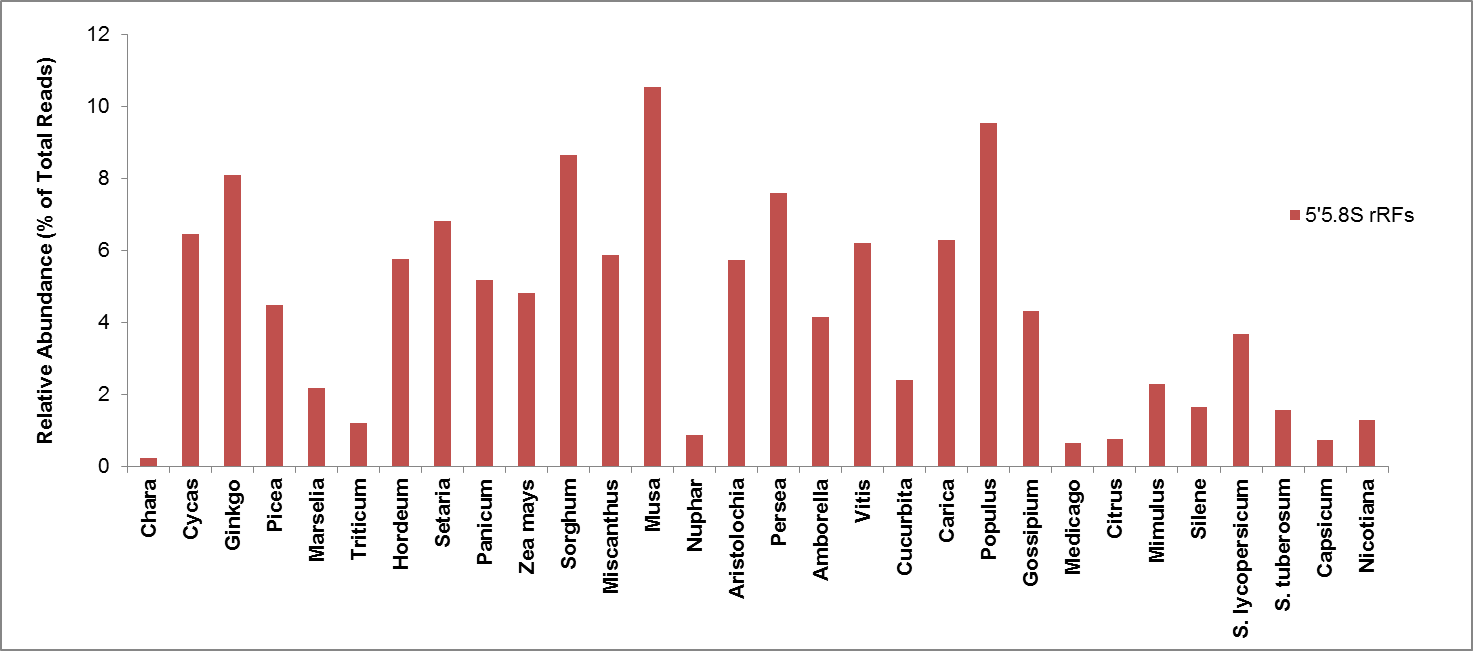


**Supplementary Figure 7:** The abundance of 5’5.8S rRF AACGACUCUCGGCAACGGAUAUCU in the 153 sRNA libraries accessed from the Arabidopsis next gen sequence database (https://mpss.danforthcenter.org).


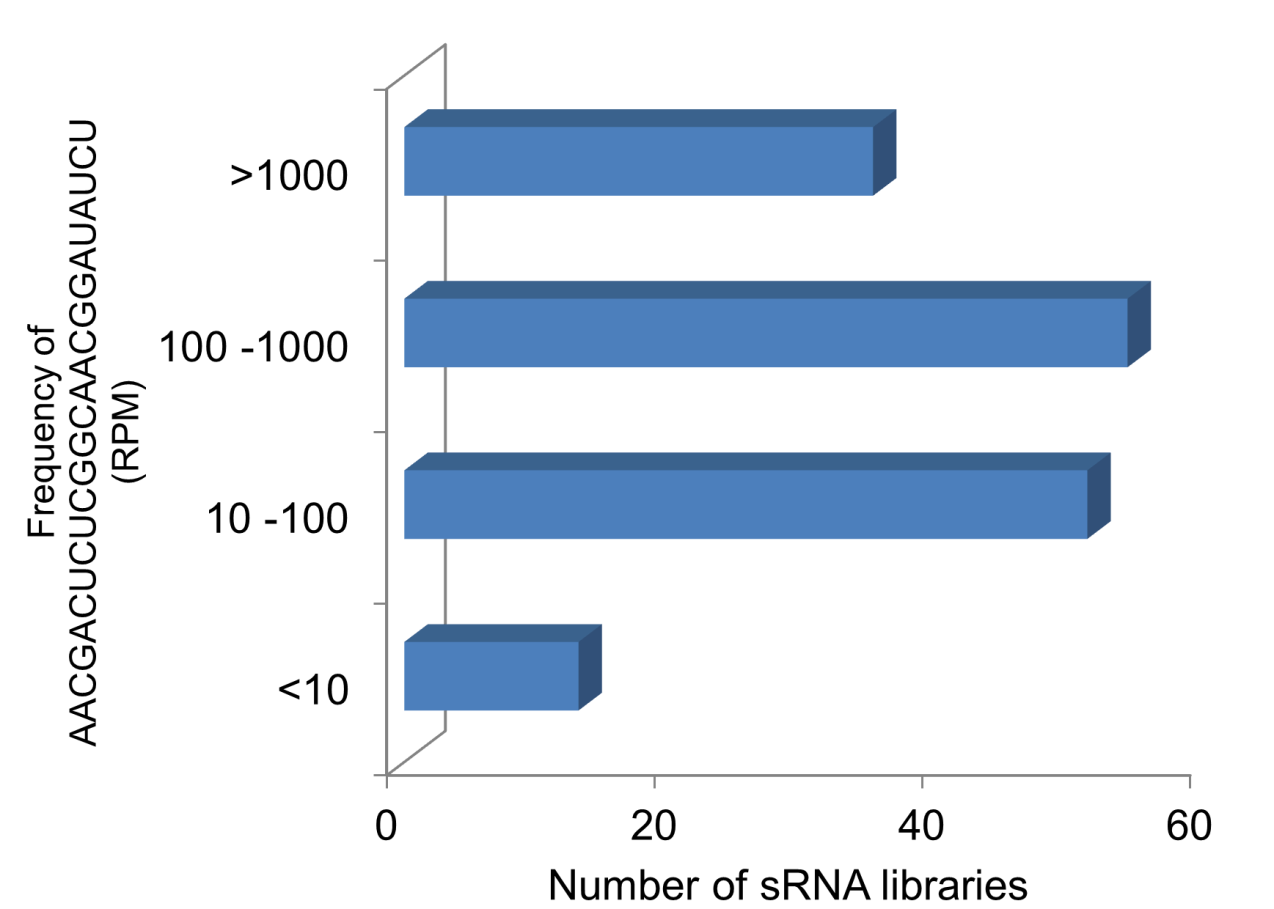


**Supplementary Figure 8: The relative occurrence of 5'5.8S rRFs among the different AGO complexed sRNA libraries.** The sRNA data (GSM707682, GSM707686, GSM304284, GSM415789 and GSM415791) from the studies of Wang et al., 2011, Montgomery et al., 2008 and Havecker et al., 2010 were analysed and the total read counts of 5**'**5.8S rRFs is represented as the normalised read count (RPM) from the corresponding sRNA library.


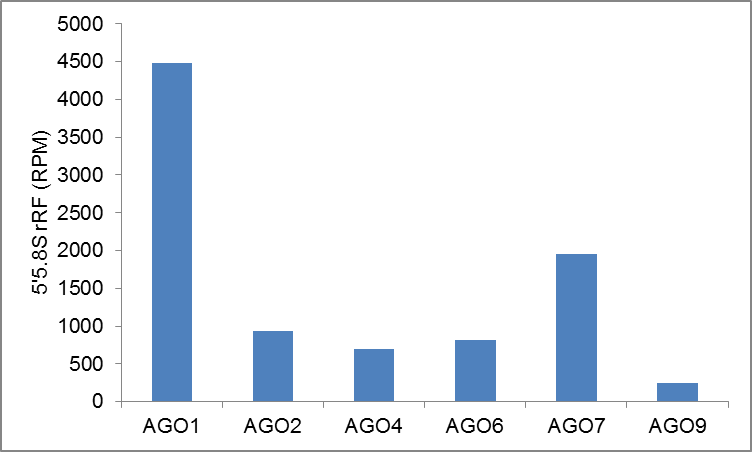
**.**

**Supplementary Figure 9: Mapping of the 5' ends of 5.8S rRNA by modified 5'RLM RACE experiments.** A) The amplified products from 5**'**RLM RACE experiments. Lane 1 represents the PCR amplified product and Lane 2 represents the 100bp ladder (NEB). Amplicons of ~150bp was eluted and sequenced B) The major cleavage site was mapped 6nt upstream of the consensus ‘GACUCUC’ in black pepper.

**
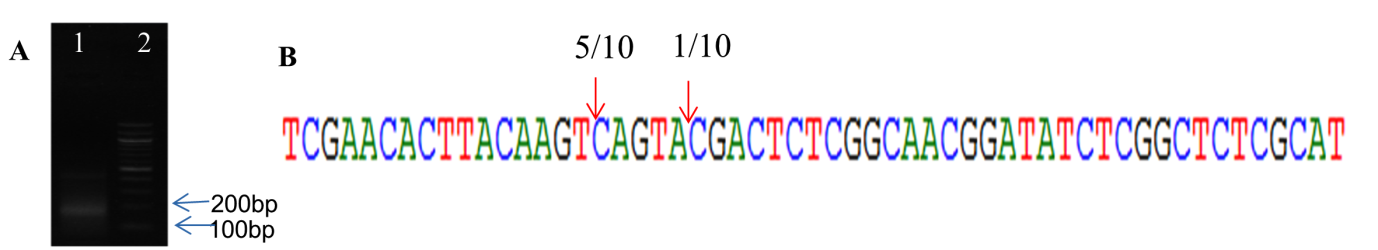
**

**Supplementary Figure 10. Non canonical hairpin like secondary structures predicted from the 5.8S rRNAs of different plants**. 5'5.8S rRFs are highlighted in red and the next abundant rRF identified was highlighted in blue.

*
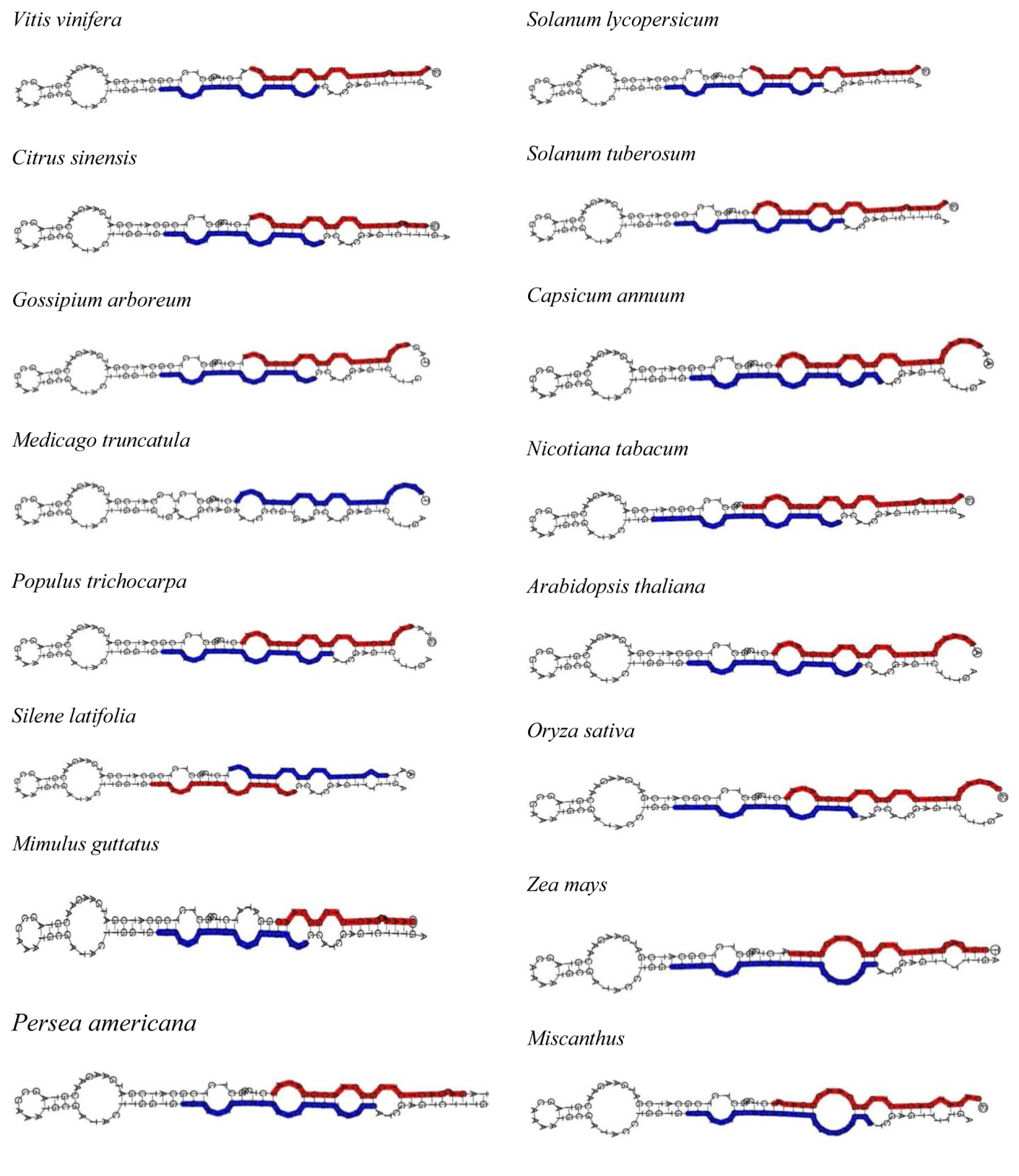
*

**Supplementary Figure 11: The expression of RPS13 mRNAs assessed from the *P. nigrum* transcriptome data.** The expression of four variants of RPS13 mRNA transcripts were represented with the FPKM values from the Pn_IL- *P. capsici* infected *P. nigrum* Leaf Transcriptome, Pn_IR- *P. capsici* infected root Transcriptome and Pn_CL- Control uninfected Leaf Transcriptome.

**Supplementary Figure 12.** The degradome RNA sequences identified from the target sites of 5’ 5.8S rRF at AT5G45775.1 (Transcript encoding ribosomal L5P family protein)

**Supplementary Figure 13. The mapping of sRNA Mediated cleavage on the other predicted targets of Arabidopsis**

**Supplementary Figure 14.Alignment, star profile plots and consensus structure of 5.8S rRNA from different lineages of plants**. (A) Multiple Alignment of 5.8S_L_ rRNA is annotated with consensus secondary structure. (B) STAR (Sequence Structure based Alignment Reliability) profile plot of the 5.8S_L_ rRNA sequence alignments depicts structure reliability (Dark regions) and sequence reliability (light regions). (C) LocARNA-P predicted consensus structure of the alignment. Hue shows the sequence conservation and saturation shows the structure conservation. The consensus boundary starts from ‘GACTCTC’.
